# Prevalence and predictors of medical information avoidance: a systematic review and meta-analysis

**DOI:** 10.1093/abm/kaaf058

**Published:** 2025-08-10

**Authors:** Konstantin Offer, Natalia Oglanova, Lisa Oswald, Ralph Hertwig

**Affiliations:** Center for Adaptive Rationality (ARC), Max Planck Institute for Human Development, 14195 Berlin, Germany; Max Planck School of Cognition, 04103 Leipzig, Germany; Department of Psychology, Humboldt-Universität zu Berlin, 10099 Berlin, Germany; Department of Psychology, The University of Potsdam, 14476 Potsdam, Germany; Center for Adaptive Rationality (ARC), Max Planck Institute for Human Development, 14195 Berlin, Germany; Center for Adaptive Rationality (ARC), Max Planck Institute for Human Development, 14195 Berlin, Germany

**Keywords:** medical information avoidance, prevalence, predictors, systematic review, meta-analysis, health behavior

## Abstract

**Background:**

Medical information avoidance—the prevention or delay of acquiring health-related information—is a growing concern for physicians, healthcare professionals, and policymakers. Yet, its prevalence and predictors have remained poorly understood.

**Purpose:**

We conducted a systematic review and meta-analysis to clarify the prevalence and predictors of medical information avoidance, offering key insights into the worldwide empirical evidence.

**Methods:**

We performed a systematic search, preregistered on the OSF and in accordance with PRISMA and MOOSE reporting guidelines. Additional individual participant datasets were obtained from the National Institutes of Health (NIH). Data analysis was performed using random-­effects and mixed-effects models.

**Results:**

A total of 92 studies and 6 individual participant datasets (564 497 unique participants, 25 countries) were analyzed. We found that almost 1 in 3 participants avoided or were likely to avoid information. Specifically, we estimated prevalence rates of 24% for diabetes, 29% for cancer, 32% for HIV, 40% for Huntington’s disease, and 41% for Alzheimer’s disease. We did not find any reliable association with gender or with race and ethnicity. Instead, we identified 16 significant predictors across cognitive, health-related, and sociodemographic domains. The strongest predictors were all cognitive: information overload (*r *= 0.26), perceived stigma (*r *= 0.36), self-efficacy (*r *= −0.28), and trust in the medical system (*r *= −0.25).

**Conclusions:**

Nearly 1 in 3 participants avoided or were likely to avoid medical information. The highest prevalence rates were found for Huntington’s disease and Alzheimer’s disease, 2 incurable neurodegenerative diseases. Key cognitive predictors suggest entry points for policy interventions and future research.

**Lay Summary:**

Medical information is more accessible than ever, but many people choose to avoid it. How common is this behavior, and what predicts it? To find out, we analyzed data from over 90 studies involving more than half a million people across 25 countries. We found that nearly 1 in 3 people avoided or were likely to avoid medical information. Avoidance was highest for incurable neurodegenerative diseases (Alzheimer’s disease: 41%, Huntington’s disease: 40%), moderate for severe but treatable conditions (HIV: 32%, cancer: 29%), and lowest for a chronic, manageable illness (diabetes: 24%). We identified 16 key predictors of medical information avoidance. Surprisingly, gender, race, and ethnicity were not among them. Instead, the strongest predictors were cognitive and emotional: mistrust in the medical system, feeling overwhelmed, low confidence in managing one’s health, and fear of being judged. Patterns of avoidance varied across world regions, suggesting that differences in healthcare systems may influence behavior. In this study, we do not judge whether medical information avoidance is good or bad. Instead, we offer the first comprehensive review of how common it is and what predicts it. More research is needed to understand the psychological and medical consequences of avoiding medical information.

## Introduction

James Watson, one of the discoverers of the DNA double helix, chose to have his genome sequenced under the condition that information about apolipoprotein E be redacted based on concerns about its association with Alzheimer’s disease.^1^ Late-onset Alzheimer’s disease (LOAD) is heritable and currently incurable.^2^ Why would a scientific truth-seeker turn away from LOAD-specific information^3^?

Medical information has become increasingly accessible since the rise of the information age.^4^ Individuals can take genetic tests, receive diagnoses, track their medical risks, and share health-related information faster than ever. Yet, people sometimes prefer not to receive medical information.^5-9^ This includes refusing cancer screenings, avoiding physicians (ie, delaying or evading care from medical professionals), and failing to return for human immunodeficiency virus (HIV) test results. We define information avoidance as “any behavior designed to prevent or delay the acquisition of available but potentially unwanted information”.^10^ Global prevalence estimates have been lacking, despite their critical role in policymaking (see Howell et al.^11^ for a qualitative summary), and aggregated predictor estimates have been limited (see Ai et al.^12^ and Li^13^ for notable exceptions). To equip physicians, healthcare professionals, policymakers, and researchers with a structured summary of the worldwide empirical evidence, we conducted a systematic review and meta-analysis on medical information avoidance, estimating key predictors and prevalence rates across 5 conditions: cancer, HIV, Huntington’s disease, diabetes, and Alzheimer’s disease.

Our systematic review and meta-analysis complements reviews on online health information-seeking behavior^14^ in relation to the quality^15^ and credibility^16^ of information, dependencies on health literacy,^17^ and implications for patient–physician relationships.^18^ It also adds to meta-analyses on cancer information seeking,^19^ risk information avoidance,^20^ cyberchondria,^21^ and antecedents of online health information seeking.^22^ This study estimates the prevalence and predictors of medical information avoidance, identifying key entry points for policy interventions and future research.

## Methods

This systematic review and meta-analysis followed the Preferred Reporting Items for Systematic Reviews and Meta-Analyses (PRISMA) and Meta-Analysis of Observational Studies in Epidemiology (MOOSE) reporting guidelines. It was preregistered prior to data collection on the Open Science Framework (OSF).

### Data source and study selection

**Figure 1.**
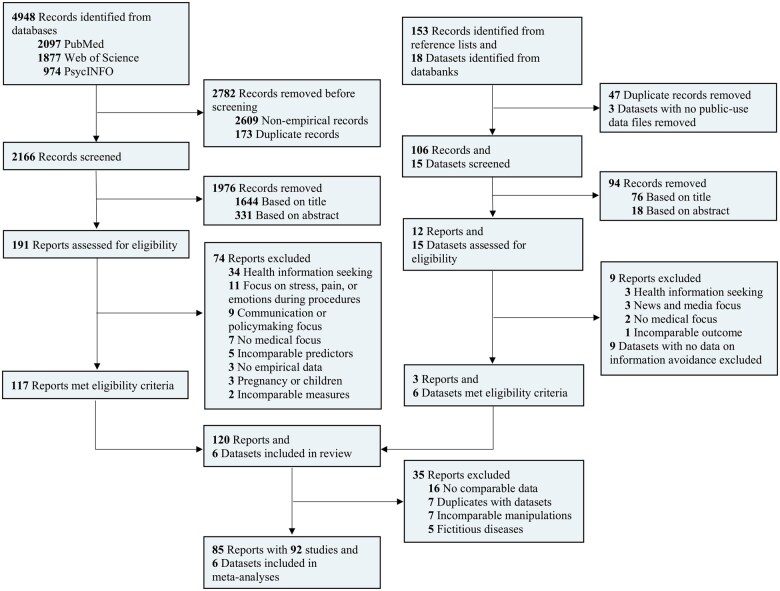
PRISMA flowchart of the study selection process, detailing the number of reports and datasets identified, screened, assessed for eligibility, and included in the meta-analysis, with reasons for exclusions at each stage.

We conducted a systematic search via PubMed, Web of Science, and PsycINFO (Figure 1). The search queries were developed in consultation with a trained librarian and are detailed in the preregistered protocol on the OSF. To strengthen our meta-analysis with primary data, we analyzed data from the Health Information National Trends Survey (HINTS). HINTS collects nationally representative data on Americans’ knowledge, attitudes, and behaviors related to cancer and health information. To identify other potentially relevant studies, we issued calls through mailing lists of medical and psychological societies and reviewed the reference lists of relevant reviews on medical information avoidance.^11-13^

To be eligible, studies needed to be in English, empirical, and contain prevalence or predictor data on medical information avoidance. No restrictions were placed on geographical region or publication year. The preregistered search was performed on March 28, 2024, and the data collection continued through November 2024. All identified records were imported into Zotero. HINTS data were sourced from the National Cancer Institute, an NIH division. Following duplicate removal and the exclusion of non-empirical texts, we screened all remaining records by title and abstract. Full-text versions of all records that passed the initial screening were retrieved. Two independent raters assessed all retrieved reports for eligibility. Studies that contained quantitatively comparable data for real diseases were included in the meta-analysis. Disagreements were resolved through discussion.

### Data extraction

Two raters extracted 4 types of data: risk of bias data, descriptive data, prevalence data, and predictor data. First, for risk of bias, we extracted data on preregistrations, open-data practices, sampling strategies, sample sizes, and reported conflicts of interest to assess possible variations in results across these dimensions. Second, we extracted data on sample types, study types, countries, and diseases to characterize the included studies. Following our preregistration, we also extracted text data on the normative stances towards medical information avoidance, coding reports as neutral, normatively favorable, or adverse, for a separate manuscript (see Additional Information). Third, we extracted prevalence rates and corresponding subsample sizes from all studies containing prevalence data to analyze the pervasiveness of medical information avoidance. Finally, we extracted effect sizes, corresponding subsample sizes, predictor variables, and outcome measures from all studies containing predictor data to conduct meta-analyses on these relationships.

For missing data, we contacted the authors and asked them for zero-order correlation coefficients. If authors did not reply within 3-5 weeks, we sent a reminder, which was typically followed by a second reminder after another 3-5 weeks. In instances where estimates were available from both HINTS data and reports based on that data, we prioritized the primary data and excluded the secondary data. The extracted data were compared for consistency, and any disagreements between raters were resolved through discussion. We present the 92 studies and 6 datasets included in our meta-analysis in Table 1, and have made all data publicly available on the OSF.

**Table 1.** Overview of the 92 studies and 6 datasets included in the meta-analysis, with descriptive data on the number of prevalence and predictor estimates, along with sample sizes, conditions, study types, and countries.

Source	Sample size	Prevalence estimates	Predictor estimates	Condition	Study type	Country
Babul et al.^54^	250	1	0	Huntington’s	Observational	Canada
Barbour et al., Study 1^48^	507	1	0	Other	Observational	United States
Barbour et al., Study 2^48^	418	1	0	Other	Observational	United States
Bernhardt et al.^55^	478	1	0	Huntington’s	Observational	Germany
Best et al.^56^	1299	1	0	Cancer	Observational	Australia
Boyd et al.^57^	525	1	2	Cancer	Observational	United States
Cannella et al.^58^	165	1	0	Huntington’s	Observational	Italy
Chae, Study 2^59^	1130	0	2	Cancer	Observational	South Korea
Chae, Study 1^60^	384	0	3	Cancer	Observational	United States
Chae, Study 2^60^	1130	0	3	Cancer	Panel study	South Korea
Chae et al.^61^	796	1	11	Cancer	Panel study	United States
Chen et al.^62^	561	0	2	COVID-19	Observational	United States
Cheong et al.^63^	223	1	0	HIV	Observational	United States
Dahl et al.^64^	432	1	3	HIV	Observational	Uganda
De Rooij et al.^65^	395	0	3	Cancer	Quasi-experiment	The Netherlands
DeGraft-Johnson et al.^66^	1516	1	0	HIV	Observational	Malawi
Dinh et al.^67^	500	1	3	HIV	Observational	Vietnam
Donders et al.^68^	478	1	0	Other	Observational	Belgium
Dong et al.^69^	1000	0	2	Other	Observational	China
Dwyer et al.^70^	257	1	3	Cancer	Observational	United States
Fernandez-Balbuena et al.^71^	6293	1	1	HIV	Observational	Spain
Flatau et al.^72^	518	2	0	Cancer	Observational	Germany
Gilmore et al.^73^	240	1	0	Other	Lab experiment	United States
Gustafson et al.^74^	1223	0	1	COVID-19	Observational	United States
Gutierrez et al.^75^	729	1	3	HIV	Observational	Mexico
HINTS 3^40^	7674	1	17	Cancer	Observational	United States
HINTS 3 Puerto Rico^41^	639	1	16	Cancer	Observational	United States
HINTS 4 Cycle 2^42^	3630	1	16	Cancer	Observational	United States
HINTS 4 Cycle 4^43^	3677	1	17	Cancer	Observational	United States
HINTS 5 Cycle 1^44^	3285	1	17	Cancer	Observational	United States
HINTS 5 Cycle 3^45^	5438	1	15	Cancer	Observational	United States
Henrikson et al.^76^	2400	1	0	Other	Observational	United States
Hightow et al.^77^	512	1	4	HIV	Observational	United States
Hooper et al.^78^	34	1	0	Alzheimer’s	Observational	United States
Howell et al., Study 1^79^	140	0	1	Other	Observational	United States
Howell et al., Study 3^79^	2119	0	1	Other	Observational	United States
Hua and Howell, Study 2^80^	326	1	0	Diabetes	Lab experiment	United States
Hua, Study 1^81^	1651	4	0	Cancer/Diabetes	Survey experiment	United States
Hua, Study 2^81^	266	3	0	Cancer/Diabetes	Survey experiment	United States
Hvidberg et al.^82^	1285	1	7	Cancer	Observational	Denmark
Ivanova and Kvalem^83^	270	0	5	Cancer	Observational	Norway
Jung, 2014^84^	502	1	7	Cancer	Observational	United States
Laanani et al.^85^	710	1	3	HIV	Observational	France
Ladner et al.^86^	1233	1	1	HIV	Observational	Rwanda
Lee^87^	800	0	5	COVID-19	Observational	Japan
Lent et al.^88^	700	1	0	Other	Observational	United States
Liddicoat et al.^89^	9129	1	3	HIV	Observational	United States
Lipsey and Shepperd, Study 2^90^	335	1	4	Other	Observational	United States
Lipsey and Shepperd, Study 1 ^91^	338	0	3	Other	Survey experiment	United States
Lipsey and Shepperd, Study 2^91^	393	0	3	Other	Survey experiment	United States
Liu et al.^92^	1031	0	2	COVID-19	Observational	China
Liu et al.^93^	362	0	3	COVID-19	Observational	China
Loiselle^94^	2142	1	2	Cancer	Observational	Canada
Lu et al., Study 2^95^	3080	0	5	Cancer	Observational	China
Lyter et al.^96^	2047	1	0	HIV	Observational	United States
Marlow et al.^97^	3113	1	4	Cancer	Observational	United Kingdom
McCloud et al.^98^	519	1	6	Cancer	Observational	United States
McQueen et al.^99^	287	0	7	Cancer	Observational	United States
Meissen and Berchek^100^	56	1	0	Huntington’s	Observational	United States
Melnyk, Study 1^101^	102	1	1	Cancer	Survey experiment	United States
Miles et al.^102^	1442	0	7	Cancer	Observational	United Kingdom
Mmbaga et al.^103^	1528	1	3	HIV	Observational	Tanzania
Molitor et al.^104^	366280	1	2	HIV	Observational	United States
Moreira Vasconcelos et al.^105^	775	1	4	Cancer	Observational	Brazil
Morris et al.^106^	1013	1	0	Cancer	Observational	United States
Msuya et al.^107^	2654	1	3	HIV	Observational	Tanzania
Nelissen et al.^108^	2008	1	6	Cancer	Observational	Belgium
Nolte et al.^109^	500	0	7	COVID-19	Observational	United States
Orom et al., Study 1^110^	1005	2	0	Cancer/Diabetes	Panel study	United States
Orom et al., Study 2^110^	600	2	0	Cancer/Diabetes	Panel study	United States
Orom et al.^111^	200	0	2	Cancer	Observational	United States
Peltzer et al.^112^	930	1	0	HIV	Observational	South Africa
Peng et al.^113^	681	0	3	Cancer	Observational	United States
Pinner and Bouman^114^	50	1	0	Alzheimer’s	Observational	United Kingdom
Pisculli et al.^115^	1959	1	7	HIV	Observational	United States
Price et al.^116^	491	0	2	HIV	Observational	United States
Rao et al.^117^	1880	1	2	HIV	Observational	South Africa
Rolland et al.^118^	214	1	3	HIV	Observational	France
Schoonen et al.^119^	510	1	0	Other	Observational	Netherlands
Schupmann et al.^120^	231	1	0	Other	Observational	United States
Sesay and Chien^121^	1755	1	5	HIV	Observational	The Gambia
Shepperd et al.^122^	315	0	2	Cancer	Observational	United States
Sherr et al., Study 1^123^	2710	1	0	Other	Observational	United Kingdom
Siebenhaar et al.^124^	1059	0	5	COVID-19	Observational	Germany
Simon et al.^125^	20125	1	3	HIV	Observational	United States
Simon et al.^126^	483	0	5	HIV	Observational	United States
Stuart, Study 1^127^	129	1	0	Cancer	Lab experiment	United States
Suan^128^	430	1	1	Diabetes	Observational	Malaysia
Sun et al.^129^	804	1	0	Cancer	Observational	South Korea
Tucker et al.^130^	223	1	2	HIV	Observational	United States
Van de Wal et al.^131^	307	1	4	Cancer	Observational	Netherlands
Van der Steenstraten et al.^132^	104	1	0	Huntington’s	Observational	Netherlands
Vandormael et al.^133^	65473	1	0	HIV	Observational	South Africa
Vrinten et al.^134^	1258	1	6	Cancer	Observational	United Kingdom
Welkenhuysen et al.^135^	167	1	0	Alzheimer’s	Observational	Belgium
Wolff and Walter^136^	29	1	0	Huntington’s	Observational	Germany
Zhang et al.^137^	557	0	3	COVID-19	Observational	China
Zhao and Liu^138^	1946	0	3	COVID-19	Observational	China

### Statistical analysis

Data analysis was performed on 92 studies from 85 reports and 6 individual participant datasets using random-effects and mixed-effects models. A pooled prevalence rate and meta-analytic predictor estimates were derived across all observed conditions, including neurodegenerative disorders (eg, Alzheimer’s disease) and infectious diseases (eg, COVID-19). Meta-analytic prevalence rates were further specified for 5 individual conditions: cancer, HIV, Huntington’s disease, diabetes, and Alzheimer’s disease.

We used Pearson’s product-moment correlation coefficient (*r*) as a standardized measure of effect size for meta-analytic predictors. In instances where effects were provided in different metrics, we converted them into *r* using appropriate conversion formulas.^23-25^ To ensure robustness, we also derived all meta-analytic estimates after applying Fisher’s *z* transformations.^26^ To prevent any single study from contributing multiple times to the same meta-analytic estimate, we aggregated dependent and independent subsamples while accounting for varying subsample sizes.^27^ We provide an overview of all transformation and aggregation formulas used in Appendix S1.

We conducted all analyses in R (version 4.4.1; R Project for Statistical Computing) using the metafor^28^ and metaBMA^29^ packages. For frequentist analyses, we fitted random-effects and mixed-effects models with restricted maximum likelihood estimators.^30^^,^^31^ We used 2-sided tests with a significance level of a=.05, and report corresponding 95% confidence intervals (CIs). For Bayesian analyses, we fitted random-effects models, using beta priors (a=2, ݼ=2), rescaled to the interval [−1, 1] for correlation coefficients.^32^^,^^33^ We completed a heterogeneity analysis, using τ^2^ and *I*^2^ statistics^34^ and Cochran’s *Q* test.^35^ We present the results of our risk of bias assessment in Appendix S2 and the results of our heterogeneity analysis in Appendix S3. An analysis of publication bias was completed using Rosenthal’s fail-safe *N,*^36^ Egger’s test,^37^ and Duval and Tweedie’s trim and fill method.^38^^,^^39^ We present these results in Appendix S4. We have made all analysis pipelines publicly available on the OSF.

## Results

The systematic search yielded 4948 records. We identified another 153 records from reference lists and 18 HINTS datasets. After removing duplicates and non-empirical texts, 2272 records remained for screening. We retrieved and assessed 203 reports for eligibility, and 120 were included in the review. 6 HINTS datasets met our eligibility criteria.^40-45^ We based our meta-analysis on 85 reports consisting of 92 studies and 6 datasets. The included studies provided 350 predictors and 84 prevalence estimates.

### Description of the studies

**Figure 2.**
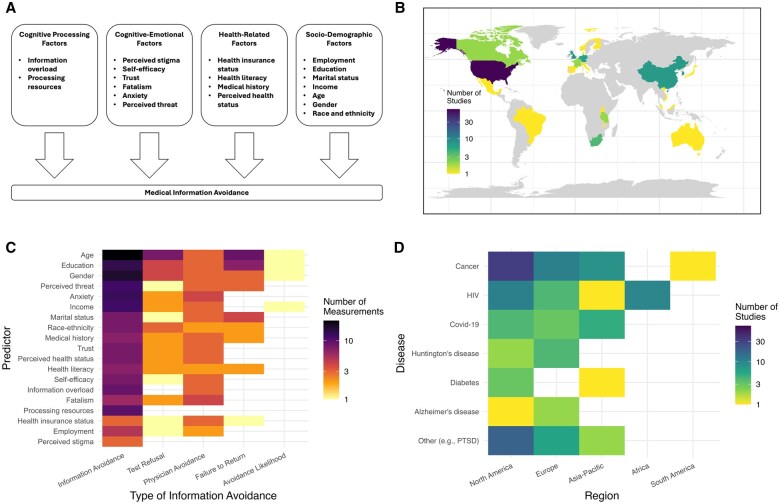
Conceptual model for the predictors of medical information avoidance (A) and description of the data: (B) geographic distribution, (C) predictors per information avoidance type, and (D) diseases per region.

The studies included in our meta-analysis were conducted in 25 countries, spanning across 5 world regions: North America, Europe, Asia-Pacific, Africa, and South America. We obtained predictor estimates across 19 dimensions (Figure 2). We grouped the predictors into 4 domains: cognitive processing, cognitive-emotional, health-related, and sociodemographic, building on earlier studies.^12^^,^^22^ We provide specifications for each predictor, along with exemplary question labels, in Appendix S5. We included 5 subtypes of medical information avoidance: information avoidance, avoidance likelihood, physician avoidance, test refusal, and failure to return. A total of 564 497 participants were included. The included studies ranged from 1987 to 2024. Cancer was the most studied disease (40%), and the United States was the most studied country (51%). Empirical evidence from South America and Africa was limited. The samples were drawn from various populations, including student (9%), clinical (17%), high-risk (26%), and general (46%) populations, as well as combinations of these groups (2%). Examples of these populations include undergraduates, patients, carriers of the Huntington’s disease gene, and non-student adults. Information regarding socioeconomic status (SES) was not consistently reported. Preregistrations were listed for 3% of studies, while 12% provided open data. Twenty percent of the reports were purely descriptive. The remainder adopted normative stances on medical information avoidance, typically evaluating the behavior negatively. No conflicts of interest were reported in 46% of the cases. The remainder did not provide any details. Most studies were observational (87%). The sampling was non-random in 82% of cases and reported to be random in 18%.

### Prevalence of medical information avoidance

**Figure 3.**
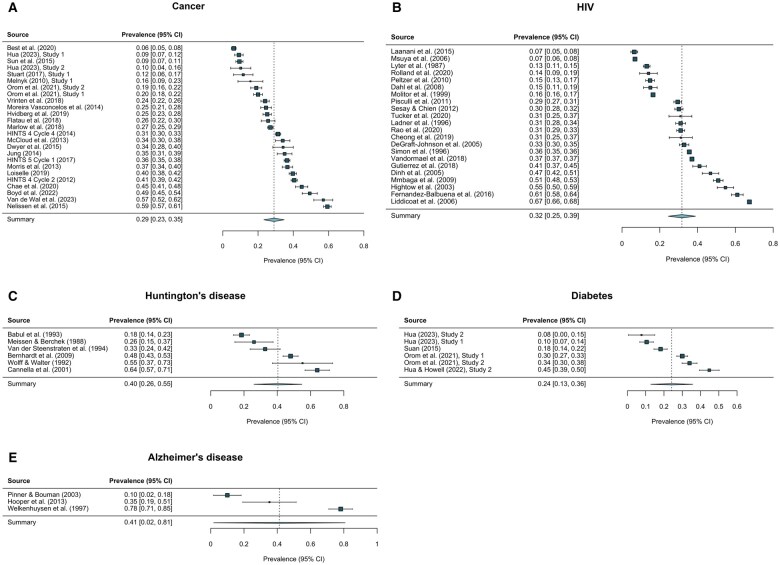
Prevalence of medical information avoidance with 95% confidence intervals (CIs) for cancer (A), HIV (B), Huntington’s disease (C), diabetes (D), and Alzheimer’s disease (E). Box sizes represent weights in meta-analytic estimates.

We estimated the prevalence of medical information avoidance using 5 inverse-variance weighted random-effects models for cancer, HIV, Huntington’s disease, diabetes, and Alzheimer’s disease. Results are presented in forest plots (Figure 3). The available data ranged from 3 studies for Alzheimer’s disease to 25 studies for cancer. The prevalence estimates were 24.3% for diabetes (*P *< .001; 95% CI, 12.8%–35.7%), 29.1% for cancer (*P *< .001; 95% CI, 23.3%–34.8%), 31.7% for HIV (*P *< .001; 95% CI, 24.6%–38.9%), 40.4% for Huntington’s disease (*P *< .001; 95% CI, 26.0%–54.8%), and 41.3% for Alzheimer’s disease (*P *= .041; 95% CI, 1.8%–80.7%). The pooled prevalence across all observations was 30.3% (*P *< .001; 95% CI, 26.5%–34.1%).

### Predictors of medical information avoidance

We estimated the predictors of medical information avoidance using 19 inverse-variance weighted random-effects models for cognitive processing, cognitive-emotional, health-related, and sociodemographic factors (Table 2). Robustness checks, performed after applying Fisher’s *z* transformations, are provided in Appendix S6.

**Table 2.** Predictors of medical information avoidance, based on Pearson’s *r*, with 95% confidence intervals (CIs).

							95% CI		Heterogeneity		
Predictor	*k*	*N*	*r̄*	*r* (range)	ES	SE	Low	High	*Q*	*I* ^2^	fsn
Cognitive processing factors											
Information overload	12	26 573	0.26	[0.11, 0.48]	0.26***	0.03	0.19	0.32	303.79	96.82	110
Processing resources	10	6350	−0.20	[−0.40, −0.06]	−0.20***	0.04	−0.28	−0.12	110.57	89.79	34
Cognitive-emotional factors											
Perceived stigma	3	1782	0.36	[0.22, 0.45]	0.36***	0.07	0.23	0.49	12.33	89.07	4
Self-efficacy	12	28 099	−0.28	[−0.47, −0.10]	−0.28***	0.04	−0.36	−0.21	542.49	97.82	107
Trust	13	26 978	−0.25	[−0.73, 0.01]	−0.25***	0.06	−0.36	−0.14	676.28	98.92	41
Fatalism	12	28 221	0.20	[0.03, 0.32]	0.20***	0.03	0.15	0.26	232.94	95.87	93
Anxiety	19	31 784	0.14	[−0.25, 0.67]	0.14**	0.05	0.04	0.23	1461.27	98.62	20
Perceived threat	19	32 510	0.00	[−0.42, 0.28]	0.00	0.03	−0.07	0.07	382.04	97.17	0
Health-related factors											
Health insurance status	8	24 428	−0.13	[−0.34, 0.09]	−0.13*	0.05	−0.23	−0.02	481.52	98.51	4
Health literacy	13	18 572	−0.12	[−0.26, 0.20]	−0.12***	0.03	−0.19	−0.06	146.07	94.45	25
Medical history	14	30 701	−0.12	[−0.27, 0.13]	−0.12***	0.03	−0.17	−0.07	206.38	95.04	47
Perceived health status	13	27 431	−0.11	[−0.23, 0.02]	−0.12***	0.02	−0.15	−0.08	127.81	88.72	63
Sociodemographic factors											
Employment	8	11 011	−0.16	[−0.37, −0.04]	−0.15***	0.03	−0.21	−0.09	38.37	87.22	26
Education	29	51 340	−0.10	[−0.47, 0.22]	−0.10**	0.03	−0.16	−0.03	1317.89	98.18	33
Marital status	16	26 680	−0.10	[−0.40, 0.06]	−0.09***	0.03	−0.15	−0.04	209.58	94.88	25
Income	18	31 768	−0.09	[−0.35, 0.22]	−0.09***	0.03	−0.15	−0.04	282.68	95.62	28
Age	43	448 996	−0.05	[−0.40, 0.27]	−0.05*	0.02	−0.10	−0.01	3084.30	98.70	19
Gender	28	64 007	0.00	[−0.20, 0.25]	0.00	0.02	−0.03	0.04	419.08	95.25	0
Race and ethnicity	15	421 061	0.00	[−0.24, 0.25]	0.00	0.03	−0.07	0.07	2511.45	99.23	0

Abbreviations: ES, estimated effect size; fsn, Rosenthal’s fail-safe *N*; *k*, Number of effects; *N*, aggregate sample size; *Q*, Cochran’s Q; *r̄*, mean effect size; SE, standard error of estimated effect size.

* *P *< .05;

** *P *< .01;

*** *P *< .001.

We found 16 significant predictors. We present the results in descending order of absolute estimated effect size, first at the group level and then for predictors within each group. In terms of cognitive processing factors, information overload (*r *= 0.26, *P *< .001) and processing resources (*r *= −0.20, *P *< .001) significantly predicted medical information avoidance. Perceived stigma (*r *= 0.36, *P *< .001), self-efficacy (*r *= −0.28, *P *< .001), trust in the medical system (*r *= −0.25, *P *< .001), fatalism (*r *= 0.20, *P *< .001), and anxiety (*r *= 0.14, *P *< .01) were significant cognitive-emotional factors. Significant health-related factors included having health insurance (*r *= −0.13, *P *< .05), being health literate (*r *= −0.12, *P *< .001), having a medical history (*r *= −0.12, *P *< .001), and a perceived “good” health status (*r *= −0.12, *P *< .001). Employment (*r *= −0.15, *P *< .001), education (*r *= −0.10, *P *< .01) marital status (*r *= −0.09, *P *< .001), high income (*r *= −0.09, *P *< .001), and age (*r *= −0.05, *P *< .05) were significant sociodemographic factors. Perceived threat (*r *= 0.00, *P *= .981), gender (*r *= 0.00, *P *= .804), and race and ethnicity (*r *= 0.00, *P *= .936) did not predict medical information avoidance. We present one forest plot per predictor in Appendix S7 (Figures S1–S19), and one funnel plot per predictor in Appendix S8 (Figures S20–S38).

We used 3 Bayesian random-effects models to evaluate the evidence for zero associations ($H_{0}:r=0)$ versus non-zero associations ($H_{1}:r?0)$ between medical information avoidance and race and ethnicity, gender, and perceived threat. Bayes factor analyses suggested null effects for race and ethnicity (${BF}_{1}$= 15, *r *= 0.00, 95% credible interval [CrI]; −0.08 to 0.07), gender (${BF}_{2}$= 25.7, *r *= 0.01; 95% CrI, −0.04 to 0.04), and perceived threat (${BF}_{3}$ = 15.6, *r *= 0.00, 95% CrI; −0.07 to 0.07). These findings provide strong evidence that medical information avoidance is not reliably related to race and ethnicity, gender, and perceived threat.^46^

## Discussion

We conducted a systematic review and meta-analysis to examine the prevalence and predictors of medical information avoidance. Our objective was to provide physicians, healthcare professionals, policymakers, and researchers with a comprehensive overview of the worldwide empirical evidence on medical information avoidance. Our analysis of 92 studies and 6 individual participant datasets (564 497 unique participants) revealed that nearly 1 in 3 participants avoided or were likely to avoid medical information. We observed a lower prevalence of medical information avoidance for diabetes (24%), a manageable, chronic disease, compared to 2 progressive, incurable neurodegenerative diseases with limited treatment options (Huntington’s disease: 40%; Alzheimer’s disease: 41%). The prevalence estimates for cancer (29%) and HIV (32%)—2 severe medical conditions that are often incurable but have treatments capable of extending life expectancy and improving quality of life—were intermediate.

We found 16 significant predictors of medical information avoidance across cognitive, health-related, and sociodemographic domains. Information overload (*r *= 0.26) and perceived stigma (*r *= 0.36), such as from a positive HIV test, showed the largest positive associations with medical information avoidance. Trust in the medical system (*r *= −0.25) and self-efficacy (*r *= −0.28) yielded the largest negative ones. We found strong evidence for null associations between medical information avoidance and race and ethnicity (${BF}_{1}$ = 15) and gender (${BF}_{2}$ = 25.7), contrary to single studies reporting both positive and negative associations (Figures S18 and S19). We did not find any evidence of publication bias that would significantly alter results (Appendix S4).

Our findings extend existing research on medical information avoidance. Building on qualitative summaries of avoidance rates for HIV^10^ and cancer,^11^ we derived meta-analytic estimates across 5 major health conditions based on a systematic literature review. This provides an empirical foundation for previous speculations about the question: “How pervasive is information avoidance?”.^10^ Our pooled prevalence rate of 30%, based on 84 estimates worldwide, shows that information avoidance is widespread,^9^ far from an “anomaly in human behavior”.^47^ Rather, medical information avoidance is both prevalent and predictable by 16 factors, enhancing theoretical understanding. In contrast to online health information seeking, which is most strongly linked to instrumental factors (eg, information quality, trustworthiness, and utility),^22^ medical information avoidance is first and foremost cognitive. It is most strongly associated with emotional and cognitive processing factors—including information overload, perceived stigma, and self-efficacy—in line with risk information avoidance^20^ and echoing earlier explanations of resisting overexposure, maintaining boundaries, and accepting limits of action.^48^

Our heterogeneity analysis revealed differences between world regions that are relevant to the generalizability of our main effects and to the design of health policies worldwide (Appendix S3). Specifically, the absolute effect size for medical history was smaller in Africa (*r *= 0.03) than in North America (*r *= −0.15), whereas the absolute effect size for trust was larger in Africa (*r *= −0.73) than in North America (*r *= −0.20). These findings suggest stronger associations between information avoidance and beliefs about the reliability of medical systems, and weaker associations with records of medical information, in African countries relative to those in North America. More broadly, these findings point to structural differences in the affordability of healthcare and the availability of health infrastructure across regions. Depending on a country’s level of economic development, information avoidance may be more or less strongly linked to either trust in medical systems or reliance on medical records. Health policies should therefore consider both the nature of policy instruments and their likely efficacy within the broader economic context in which they are implemented.

We identified 3 ways to strengthen future research. First, preregistrations were only present in 3% of cases. By outlining research questions, methods, and possible limitations in advance, transparency and credibility can be enhanced. Second, open data was provided in just 12% of cases. Making data openly available whenever possible would support reproducibility, encouraging collaborations and meta-analyses. Finally, conflict of interest statements were present in only 46% of cases. Including such statements in all research could foster transparency, integrity, and trust in science.

This research informs normative debates on policy and intervention design by highlighting the strong influence of social and informational environments on medical information avoidance. Only 20% of analyzed reports were purely descriptive. Examining predictors, we found that 2 of the 3 strongest predictors were cognitive factors related to social and informational environments (ie, $r_{\mathrm{stigma}}$ = 0.36, $r_{\mathrm{overload}}$ = 0.26). We caution against fundamental attribution errors: Cognitive biases in which observers overestimate the influence of internal factors (eg, personality or character traits) and underestimate the impact of external factors (eg, situational or environmental influences).^49^^,^^50^ Medical information avoidance is not just a function of a person’s attitudes (eg, self-efficacy) and beliefs (eg, fatalism), but also closely related to their social and informational environments (eg, perceived stigma or information overload).

Medical information avoidance has important implications for policymakers around the world. Cognitive factors yielded the strongest effects, and higher levels of trust were associated with lower levels of information avoidance ($r_{\mathrm{trust}}$ = −0.25). Trust encompasses confidence in the medical system, including trust in doctors and medical information (Appendix S5). Trust in the US medical system, for instance, has declined sharply during the past half-century.^51^ While 73% of Americans had confidence in their medical leaders in 1966, only 34% expressed this view in 2012.^52^ Adults from low-income families were less trusting, while older Americans were more likely than younger Americans to agree that physicians can be trusted. Our evidence suggests that declines in trust are associated with increases in information avoidance and that restoring trust in both the US and international medical systems may be linked to greater engagement with medical information.

### Limitations

Our research has 5 important limitations.

First, as anticipated in our preregistration, the studies analyzed are not representative of the world population. Although we analyzed data from 25 countries, most evidence came from North America, Europe, and the Asia-Pacific. Data from South America and Africa were limited, and no data from the Middle East were available. This limits the generalizability of our findings.

Second, the included studies were highly heterogeneous in both methodologies (eg, nationally representative surveys vs. survey experiments) and populations (eg, high-risk vs. general populations). This variability is reflected in high heterogeneity statistics, which we were able to partially explain through subgroup analyses (Appendix S3). We hope that future research will further examine heterogeneity, contributing to more comprehensive theories and reliable guidance for policymakers.^53^

Third, evidence across medical conditions was unevenly distributed. For example, the prevalence estimate for Alzheimer’s disease was based on only 3 studies—drawn from clinical, student, and high-risk populations—compared to 25 studies for cancer (Table 1), contributing to a wide confidence interval for a less-studied condition such as Alzheimer’s disease (Figure 3). We hope that future research will generate more precise prevalence estimates for understudied conditions.

Fourth, our analysis of race and ethnicity was based on 15 studies (Figure S19 and Appendix S7), all conducted in the United States and providing comparisons between non-White and White individuals. While a Bayes factor analysis (${\mathrm{BF}}_{1}$= 15) suggested no reliable association between information avoidance and race and ethnicity, subgroup differences (eg, Black vs. Hispanic individuals), and differences in non-US contexts may still exist. This limits the generalizability of our race and ethnicity null finding.

Finally, we preregistered a third meta-analysis on medical information avoidance outcomes (eg, psychological well-being, life satisfaction, mortality) on the OSF. However, we were unable to complete it due to insufficient outcome data. We hope that future research will address this gap by systematically examining the psychological and medical implications of medical information avoidance.

### Unanswered questions

Our systematic review and meta-analysis highlights several important directions for future research. We outline key questions across 5 dimensions.

First, how do the prevalence and predictors of medical information avoidance vary across countries and cultures? Comparing world regions, we found significant differences in trust and medical history between Africa and North America. However, due to limited data, we could not assess country-specific variations reliably. We expect that both structural (eg, healthcare affordability) and cultural factors (eg, social norms) further moderate worldwide effects. Cross-cultural comparisons are promising for future research.

Second, which factors best explain intra- and inter-individual differences? Our heterogeneity analysis examined 7 factors and identified 20 moderations (Appendix S3). Nonetheless, little is known about how medical information avoidance varies within individuals over time, and beyond population-based differences.

Third, how prevalent is medical information avoidance beyond the 5 medical conditions included in this meta-analysis, and what factors drive differences across conditions? Synthesizing 84 observations, prevalence rates ranged from 24% for diabetes to 41% for Alzheimer’s disease. Yet, data on other conditions remain sparse. We expect that individual interpretations of curability and treatability play key roles, and view research into the reasons behind variations across prevalence rates as a promising direction.

Fourth, when, for whom, and under what circumstances is medical information avoidance desirable, and when is it harmful? Our focus in this systematic review and meta-analysis is descriptive and analytical. In a separate manuscript, we will analyze the normative implications discussed in the literature (see also Additional Information).

Finally, what are the consequences of medical information avoidance for individuals and societies? Many questions remain, and we are only beginning to understand medical information avoidance systematically.

## Conclusion

This systematic review and meta-analysis of 92 studies and 6 individual participant datasets found that almost 1 in 3 participants avoided or were likely to avoid medical information. This tendency was particularly pronounced for 2 neurodegenerative diseases, Huntington’s disease and Alzheimer’s disease, which currently cannot be cured. Contrary to past findings, sociodemographic factors—such as gender or race and ethnicity—did not predict medical information avoidance. The strongest predictors were all cognitive: information overload, perceived stigma, self-efficacy, and trust in the medical system, suggesting key entry points for policy interventions and future research, both individually and in combination.

## Supplementary Material

kaaf058_Supplementary_Data

## Data Availability

All data not directly obtained from the National Institutes of Health (NIH) have been made publicly available on the OSF: https://osf.io/9e8hd/. Data from the NIH can be obtained from the Health Information National Trends Survey: https://hints.cancer.gov.

## Additional information

Hertwig and Engel (2016, 2021) have proposed the construct of deliberate ignorance, defined as the conscious choice—by individuals or institutions—not to seek or use information, even when the marginal acquisition costs are negligible and the potential benefits promise to be high. Under these circumstances, the failure to obtain information may seem irrational at first sight. The literature on medical information avoidance often leaves it open to what extent the choice not to know is a deliberate one. We therefore speak of information avoidance in this study. Our focus is entirely descriptive and analytical. In a separate manuscript, we will be concerned with the literature’s discussions of the normative implications of medical information avoidance.
