# Supplementary material for: Prevalence and predictors of medical information avoidance: a systematic review and meta-analysis
Source: Ann Behav Med. 2025 Aug 10;59(1):kaaf058. doi: 10.1093/abm/kaaf058 (PMC12342947; doi:10.1093/abm/kaaf058)
Supplement: kaaf058_Supplementary_Data [file kaaf058_supplementary_data.docx]

**Supplementary Material**Prevalence and predictors of medical information avoidance:
A systematic review and meta-analysis

Konstantin Offer,^[[1]](#footnote-2)^ Natalia Oglanova,
Lisa Oswald, Ralph Hertwig

June 10, 2025

**eAppendix 1. Aggregations and transformations of effect sizes**

Included effect sizes were presented in various metrics (e.g., odds ratio, Cohen’s *d*, regression coefficient). We converted them into Pearson’s product-moment correlation coefficient (*r*) based on the R script by Thielmann et al.,^1^ 2020.^[[2]](#footnote-3)^ Here, we present the formulas we used for aggregations and transformations.

**Aggregation of multiple effects**

***Aggregation of effects for multiple independent criteria***

To aggregate effect sizes for multiple independent criteria, we computed the weighted mean correlation coefficient:

$$r_{mean.indep}= \frac{\Sigma(r_{k}\cdot n_{k})}{\Sigma n_{k}}$$

where $r_{k}$​​ is the correlation coefficient for each independent sample, and $n_{k}$​​ ​the sample size associated with each correlation. This method allows for an estimation of the correlation coefficient across independent samples, accounting for varying subsample sizes.

**A*ggregation of effects for multiple dependent criteria***

For multiple dependent criteria, we calculated the aggregated correlation coefficient using the mean of the correlations, the mean intercorrelation, and the number of criteria:

$$r_{mean.dep}= \frac{\Sigma r_{k}}{\sqrt{k+k \cdot\left( k-1 \right)\cdot r_{yy}}}$$

where $r_{k}$ are the individual correlation coefficients, $r_{yy}$​ the mean intercorrelation among the dependent criteria, and *k* the number of criteria being aggregated. This method accounts for the dependence between criteria, adjusting overall effect size estimates.

**Calculation of standard deviations**

***From standard error to standard deviation***

In cases where standard deviations (SDs) were not reported, we derived them from standard errors (SEs):

$$SD=SE \cdot\sqrt{n}$$

where *n* is the sample size. We used SDs for further transformations to *r*.

**Linear regression analyses**

***From unstandardized to standardized linear regression coefficients***

The following function converts unstandardized regression coefficients (*b*) into standardized coefficients ($\beta$), supporting comparisons across observations:

$$\beta=b\cdot\frac{{SD}_{x}}{{SD}_{y}}$$

where ${SD}_{x}$​ and ${SD}_{y}$​ are the standard deviations of the predictor and outcome variables, respectively.

***From t-values to r***

To convert t-values from linear regression to correlation coefficients, we used:

$$r= \sqrt{\frac{t^{2}}{t^{2}+df}}$$

where *df* is the degrees of freedom.

**Logistic regression analyses**

***From unstandardized to standardized logistic regression coefficients***

To transform unstandardized to standardized logistic regression coefficients, we used:

$$OR= e^{b}$$

where *b* is the unstandardized regression coefficient and *e* is Euler’s number.

**Transformations from other effect sizes to correlation coefficient**

***From z-test to r***

We transformed *z*-scores into *r*:

$$r= \frac{z}{\sqrt{n}}$$

where *n* is the sample size. This transformation allows for a consistent and comparable use of effect size estimates on the correlational scale.

***From Odds Ratio to r***

We used the following function to convert an odds ratio (OR) into *r*:

$$r= \frac{{OR}^{3/4}-1}{{OR}^{3/4}+1}$$

This transformation allows for interpretations of ORs in terms of correlations, allowing for comparisons with other effect size estimates.

***From Cohen’s d to r***

To convert Cohen's *d* into correlation coefficients, we used:

$$r= \frac{d}{\sqrt{d^{2}+ \frac{1}{\left( \frac{n_{1}}{n} \right)\cdot\left( \frac{n_{2}}{n} \right)}}}$$

where $n_{1}$​ and $n_{2}$ are the sample sizes of two groups, and *n* is the total sample size.

***From chi-square to r***

To convert chi-square statistics into correlation coefficients, we used:

$$r= \sqrt{\frac{\chi^{2}}{n}}$$

where *n* is the sample size. This transformation allows for interpretation of chi-square values on the correlational scale.

***From t-tests to r***

The following function converts *t*-test statistics into correlation coefficients, accommodating different scenarios:

$$r= \left\{ \begin{aligned} \frac{t}{\sqrt{t^{2}+df}} \\ \sqrt{\frac{t^{2}}{t^{2}+ \left( n-2 \right)}} \end{aligned} \right.$$

where *df* is the degrees of freedom. The above condition is valid for cases where multiple groups are present, and the below condition is valid otherwise. The separation accounts for differences in multiple group comparisons compared to single group analyses.

***From Spearman to Pearson***

To transform Spearman's rank correlation coefficients to Pearson's correlation coefficients, we used the following function based on Winter et al.,^2^ 2016:

$$R_{p}=2\cdot\sin\left( \frac{R_{s}\cdot\pi}{6} \right)$$

where $R_{s}$ is the Spearman's rank correlation coefficient, $R_{p}$ Pearson's correlation coefficients, and $\pi$ Archimedes’ constant.

**Calculation of Cohen's d**

To calculate Cohen's *d*, we used the following function, computing the effect size based on the means and standard deviations of two groups:

$$d= \frac{M_{1}- M_{2}}{{SD}_{\mathrm{pooled}}}$$

where $M_{1}$ and $M_{2}$ are the means of two groups and ${SD}_{\mathrm{pooled}}$ is the pooled standard deviation:

${SD}_{\mathrm{pooled}}= \sqrt{\frac{\left( {SD}_{1}^{2} \cdot\left( n_{1}-1) \right)+ \left( {SD}_{2}^{2} \cdot\left( n_{2}-1 \right) \right) \right)}{n}}$

where $n_{1}$ and $n_{2}$ are the sample sizes, ${SD}_{1}$ and ${SD}_{2}$ the standard deviations, and n is the total sample size.

**Transformations from Pearson’s r to Fisher’s z**

Finally, to transform Pearson’s *r* to Fisher’s *z*, we used:

$$z=0.5\cdot\ln\left( \frac{1+ r}{1- r} \right)$$

The results for the robustness tests based on Fisher’s *z* transformations are provided in eAppendix 6.

**eAppendix 2. Risk of bias assessment**

To assess risk of bias, and in line with our preregistration, we examined preregistrations, open-data practices, sampling strategies, sample sizes, and stated conflicts of interest as moderators using mixed-effects models. An additional analysis of publication bias is presented in eAppendix 4. The funnel plots supporting this analysis are presented in eAppendix 8. We caution against overinterpreting moderation effects in our risk of bias assessment, as they may be based on a small number of studies.

We found four types of effects:

# Preregistrations: We found that ten non-preregistered studies showed a positive effect for anxiety, whereas a single preregistered study showed a negative one (*β* = 0.46, *SE* = 0.18, *p* < 0.05; 95% *CI*, 0.82 to 0.10).

# Sampling strategies: We found larger absolute effects in cases of random sampling (compared to non-random sampling) for medical history (*β* = −0.10, *SE* = 0.05, *p* < 0.05; 95% *CI*, −0.20 to −0.01) and perceived health status (*β* = −0.08, *SE* = 0.04, *p* < 0.05; 95% *CI*, −0.15 to −0.02) and a smaller absolute effect for information overload (*β* = −0.12, *SE* = 0.06, *p* < 0.05; 95% *CI*, −0.24 to −0.00).

# Sample sizes: We found larger absolute effects by sample size, rescaled to changes per 1,000 subjects for interpretability, for information overload (*β* = −0.03, *SE* = 0.01, *p* < 0.05; 95% *CI*, −0.06 to −0.00) and perceived health status (*β* = −0.02, *SE* = 0.01, *p* < 0.05; 95% *CI*, −0.03 to −0.00).

# Conflicts of interest: We found that studies explicitly stating no conflict of interest showed a larger absolute effect for education (*β* = −0.21, *SE* = 0.07, *p* < 0.01; 95% *CI*, −0.35 to −0.07) and marital status (*β* = −0.13, *SE* = 0.06, *p* < 0.05; 95% *CI*, −0.26 to −0.00) compared to studies that provided no details on conflicts of interest.

# We cannot exclude two types of biases based on these findings, our study descriptions, and assumptions of more reliable results under preregistrations, random sampling, larger samples, and the inclusion of conflict-of-interest statements.

# Overestimations are possible for our anxiety and information overload estimates, based on preregistrations and sampling strategies, respectively.

# Underestimations are possible for our medical history and perceived health status estimates (based on sampling strategies), our information overload and, again, perceived health status estimates (based on sample sizes), and our education and marital status estimates (based on practices for stating conflicts of interest).

**eAppendix 3. Heterogeneity analysis**

We quantified between-study heterogeneity using τ^2^ and I^2^ statistics.^3^ Significance was assessed using Cochran’s Q test.^4^ The Q statistic tests if observed variation exceeds chance. The I² statistic quantifies the proportion of variability from heterogeneity rather than sampling error. We identified significant heterogeneity across all five conditions. The heterogeneity estimates were significant for diabetes (*τ*^2^ = 0.02, *I*^2^ = 97.84%, *Q*(5) = 174.00, *p* < 0.001), cancer (*τ*^2^ = 0.02, *I*^2^ = 99.27%, *Q*(24) = 3378.97, *p* < 0.001), HIV (*τ*^2^ = 0.03, *I*^2^ = 99.93%, *Q*(21) = 26263.37, *p* < 0.001), Huntington’s disease (*τ*^2^ = 0.03, *I*^2^ = 95.74%, *Q*(5) = 139.46, *p* < 0.001), and Alzheimer’s disease (*τ*^2^ = 0.12, *I*^2^ = 98.03%, *Q*(2) = 150.41, *p* < 0.001). The heterogeneity estimates for the meta-analytic predictors are presented in Table 2. To explain heterogeneity, we examined two conceptual, two methodological, and three descriptive moderators using mixed-effects models. We caution against overinterpreting moderation effects in our heterogeneity analysis, as some analyses are based on a small number of studies, which may limit the generalizability of the results.

1. Conceptual moderator

We examined medical information avoidance subtypes (i.e., information avoidance, avoidance likelihood, physician avoidance, test refusal, and failure to return) and sample types (i.e., student, clinical, high-risk, general, and other) as two conceptual moderators. Compared to information avoidance, physician avoidance yielded larger absolute effects for perceived health status (*β* = −0.10, *SE* = 0.04, *p* < 0.01; 95% *CI*, −0.17 to −0.03), health insurance status (*β* = −0.19, *SE* = 0.09, *p* < 0.05; 95% *CI*, −0.37 to −0.01), medical history (*β* = −0.10, *SE* = 0.04, *p* < 0.05; 95% *CI*, −0.19 to −0.02), employment (*β* = −0.14, *SE* = 0.05, *p* < 0.01; 95% *CI*, −0.24 to −0.04), and age (*β* = −0.22, *SE* = 0.08, *p* < 0.01; 95% *CI*, −0.38 to −0.06), and a smaller absolute effect for fatalism (*β* = −0.12, *SE* = 0.03, *p* < 0.001; 95% *CI*, −0.18 to −0.06). Similarly, test refusal yielded a smaller absolute effect for fatalism (*β* = −0.24, *SE* = 0.04, *p* < 0.001; 95% *CI*, −0.32 to −0.15) and income (*β* = 0.19, *SE* = 0.08, *p* < 0.05; 95% *CI*, 0.03 to 0.35). Failure to return yielded a smaller absolute effect for medical history (*β* = 0.13, *SE* = 0.05, *p* < 0.05; 95% *CI*, 0.02 to 0.23) and a larger absolute effect for non-whites compared to whites (*β* = 0.25, *SE* = 0.09, *p* < 0.01; 95% *CI*, 0.08 to 0.42). For sample types, we found a positive effect for high-risk samples (compared to a negative effect for general samples) for medical history (*β* = 0.27, *SE* = 0.07, *p* < 0.001; 95% *CI*, 0.13 to 0.42).

1. Methodological moderators

We assessed study types and data sources as two methodological moderators. We found a higher prevalence of medical information avoidance in observational studies, which made up the majority of all studies, compared to more controlled, non-observational studies (*β* = 0.10, *SE* = 0.05, *p* < 0.05; 95% *CI*, 0.01 to 0.19). In terms of predictors, we found a smaller absolute effect in cases of observational studies (compared to non-observational studies) for marital status (*β* = 0.17, *SE* = 0.08, *p* < 0.05; 95% *CI*, 0.02 to 0.33). For data sources, we found that HINTS data had a smaller absolute effect compared to non-HINTS data for information overload (*β* = −0.12, *SE* = 0.06, *p* < 0.05; 95% *CI*, −0.24 to −0.00).

1. Descriptive moderators

Finally, we analyzed diseases, years of publication, and world regions as three descriptive moderators. We found a smaller absolute medical history effect for HIV compared to cancer (*β* = 0.17, *SE* = 0.06, *p* < 0.01; 95% *CI*, 0.06 to 0.29) and a larger absolute information overload effect for non-cancer (e.g., COVID-19) compared to cancer (*β* = 0.02, *SE* = 0.01, *p* < 0.05; 95% *CI*, 0.00 to 0.04). We found larger absolute effects of perceived stigma (*β* = 0.04, *SE* = 0.01, *p* < 0.01; 95% *CI*, 0.01 to 0.06) and employment (*β* = 0.01, *SE* = 0.00, *p* < 0.05; 95% *CI*, 0.00 to 0.02) and a smaller absolute effect of medical history (*β* = −0.01, *SE* = 0.00, *p* < 0.01; 95% *CI*, −0.02 to −0.00) over time. In terms of regions, we found a larger absolute effect of trust (*β* = −0.53, *SE* = 0.15, *p* < 0.001; 95% *CI*, −0.83 to −0.23) and a smaller absolute effect of medical history (*β* = 0.17, *SE* = 0.07, *p* < 0.05; 95% *CI*, 0.04 to 0.31) for Africa compared to North America.

**eAppendix 4. Analysis of publication bias**

We analyzed publication bias using three methods: Rosenthal’s fail-safe N to address file drawer problems,^5^ Egger’s test to assess funnel plot asymmetry,^6^ and Duval and Tweedie’s trim and fill method to estimate the outcomes of missing studies.^7,8^

1. Rosenthal’s fail-safe N

Rosenthal’s fail-safe N is the minimum number of studies with null results needed to render meta-analytic results statistically non-significant (*p* > 0.05).^5^ We present the estimates in Table 2. By definition, fail-safe N is zero for non-significant predictors. The positive estimates range from four studies for perceived stigma and health insurance status to 110 studies for information overload. The median fail-safe N is 31, and the mean is 42. In comparison, each significant predictor is based on a median of 13 and a mean of 15 studies. Thus, more than two additional non-significant studies would, on average, be needed for each existing study to render the meta-analytic estimates non-significant.

1. Egger’s test for funnel plot asymmetry

Egger’s test is a measure to quantify funnel plot asymmetry. It measures the intercept from the regression of standard deviates against the inverse of standard errors as a measure of publication bias.^6^ We detected funnel plot asymmetry for trust (*z* = 2.61, *p* < 0.01, *b* = −0.49; 95% *CI*, −0.69 to −0.29) and marital status (*z* = −2.73, *p* < 0.01, *b* = 0.06; 95% *CI*, −0.06 to 0.17). We found no evidence for funnel plot asymmetry for the 17 remaining predictors. We present one funnel plot per predictor in eAppendix 8 (eFigures 20–38).

1. Duval and Tweedie’s trim and fill method

Duval and Tweedie’s trim and fill method is a non-parametric approach to estimate and adjust for the outcomes of missing studies.^8^ It can restore funnel plot symmetry. We have included imputed results in eFigures 20–38. After imputing results, we could no longer detect funnel plot asymmetry for trust (*z* = 0.90, *p* = 0.37, *b* = −0.39; 95% *CI*, −0.63 to −0.14), but still for marital status (*z* = −2.73, *p* < 0.01, *b* = 0.06; 95% *CI*, −0.06 to 0.17). The absolute meta-analytic effect sizes slightly increased for trust (*r* = −0.29, *p* < 0.001, *SE* = 0.06; 95% *CI*, −0.39 to −0.18) and remained unchanged for marital status. We did not find any evidence for changes in significance.

**eAppendix 5. Predictor constructs**

|  |  | HINTS | | |  |
| --- | --- | --- | --- | --- | --- |
|  | Predictor | Datasets | Variables | Exemplary label (HINTS 3) | Constructs |
|  |  |  |  |  |  |
|  | **Cognitive Processing Factors** |  |  |  |  |
|  | Information overload | 3.1, 3.2, 4.2, 4.4, 5.1, 5.3 | CS14TooManyRecommendations, TooManyRecommendations | There are so many different recommendations about preventing cancer, it`s hard to know which ones to follow. Would you say you strongly agree, somewhat agree, somewhat disagree, or strongly disagree? | Information overload, cancer information overload, COVID-19 information overload, HIV information overload |
|  | Processing resources | ––––– | ––––– | ––––– | Perceived processing resources, perceived social support, perceived coping resources, perceived interpersonal resources, perceived personal resources, emotional support, lack of perceived stress |
|  | **Cognitive-Emotional Factors** |  |  |  |  |
|  | Perceived stigma | ––––– | ––––– | ––––– | Perceived stigma, HIV stigma, HIV testing stigma |
|  | Self-efficacy | 3.1, 3.2, 4.2, 4.4, 5.1, 5.3 | HS11TakeCareHealth, OwnAbilityTakeCareHealth | Overall, how confident are you about  your ability to take good care of your  health? Would you say completely confident, very confident, somewhat confident, a little confident, or not confident at all? | Self-efficacy, general self-efficacy, coping self-efficacy, efficacy, response-efficacy, health self-efficacy, information self-efficacy, empowerment, powerfulness, resilience |
|  | Trust | 3.1, 3.2, 4.2, 4.4, 5.1, 5.3 | HC07aTrustDoctor,  CancerTrustDoctor,  TrustDoctor | In general, how much would you trust information about health or medical topics... from a doctor or other health care professional? Would you say a lot, some, a little, or not at all? | Trust, medical trust, trust in medical information, trust in doctors, belief in information reliability, belief in test reliability |
|  | Fatalism | 3.1, 3.2, 4.2, 4.4, 5.1, 5.3 | CS05CancerFatal, PreventNotPossible | When I think of cancer, I automatically think of death. Would you say you strongly agree, somewhat agree, somewhat disagree, or strongly disagree? | Fatalism, general fatalism, cancer fatalism, cancer mortality salience, cancer as a death sentence, medical hopelessness |
|  | Anxiety | 3.1, 3.2, 4.4, 5.1, 5.3 | CS07FreqWorryCancer, FreqWorryCancer | How often do you worry about getting cancer? Would you say rarely or never, sometimes, often, or all the time? | Anxiety, trait anxiety, information anxiety, fear, screening fear, symptom fear, cancer fear, cancer worry, health worry |
|  | Perceived threat | 3.1, 3.2, 4.2, 4.4, 5.1, 5.3 | CS06ChanceGetCancer, ChanceGetCancer | How likely do you think it is that you will develop cancer in the future? Would you say your chance of getting cancer is very low, somewhat low, moderate, somewhat high, or very high? | Perceived threat, perceived risk, cancer risk perception, individual risk, perceived severity, perceived susceptibility, infectiousness beliefs |
|  | **Health-Related Factors** |  |  |  |  |
|  | Health insurance status | 3.1, 3.2, 4.2, 4.4, 5.1, 5.3 | HS04Insurance,  HealthInsurance,  HealthIns_InsurancePriv | Do you have any kind of health care coverage, including health insurance, prepaid plans such as HMOs, or government plans such as Medicare? | Health insurance status, having statutory health insurance, being insured |
|  | Health literacy | 3.1, 3.2, 4.2, 4.4, 5.1 | CS02MedStats, CancerTooHardUnderstand,  TooHardUnderstand | In general, how easy or hard do you find it to understand medical statistics? Would you say very easy, easy, hard, or very hard? | Health literacy, health literary scores, medical knowledge, HIV knowledge, perceived knowledge |
|  | Medical history | 3.1, 3.2, 4.2, 4.4, 5.1, 5.3 | CS17EverHadCancer, FamilyEverHadCancer,  EverHadCancer | Have you ever been diagnosed as having cancer? | Medical history, cancer history, personal cancer history, cancer diagnosis, family cancer history, positive HIV result |
|  | Perceived health status | 3.1, 3.2, 4.2, 4.4, 5.1, 5.3 | HD01GeneralHealth,  GeneralHealth | In general, would you say your health is excellent, very good, good, fair, or poor? | Perceived health status, health status, perceived health, self-rated health |
|  | **Socio-Demographic Factors** |  |  |  |  |
|  | Employment | 3.1, 3.2, 4.2, 4.4, 5.1 | HD05OccupationStatus, OccupationStatus | What is your current occupational status? Would you say employed, unemployed, homemaker, student, retired, disabled, or other? | Employment, being employed, not being unemployed |
|  | Education | 3.1, 3.2, 4.2, 4.4, 5.1, 5.3 | HD07Education, Education | What is the highest grade or level of schooling you completed? Would you say less than 8 years, 8 through 11 years, 12 years or completed high school, post-high school training other than college (vocational or technical), some college, college graduate, or postgraduate? | Education, primary school, high school graduate, college graduate, post-graduate, being higher educated |
|  | Marital status | 3.1, 3.2, 4.2, 4.4, 5.1, 5.3 | HD06MaritalStatus,  MaritalStatus | What is your marital status? Would you say married, living as married, divorced, widowed, separated, single/never been married, or not ascertained? | Marital status, being married, ever married |
|  | Income | 3.1, 3.2, 4.2, 4.4, 5.1, 5.3 | HD15IncomeRanges, hint2009$HHInc,  IncomeRanges | Thinking about members of your family living in this household, what is your combined annual income, meaning the total pre-tax income from all sources earned in the past year? $0 to $9,999, $10,000 to $14,999, $15,000 to $19,999, $20,000 to $34,999, $35,000 to $49,999, $50,000 to $74,999, $75,000 to $99,999, $100,000 to $199,999, or $200,000 or more? | Income, annual household income, high income, financial security |
|  | Age | 3.1, 3.2, 4.2, 4.4, 5.1, 5.3 | AgeGrpB | What is your age? Would you say 18-34, 35-49, 50-64, 65-74, or 75+? (RespondentAge Recode – 5 Levels) | Age, age group, age at completion of questionnaire, being old |
|  | Race and ethnicity | 3.1, 4.2, 4.4, 5.1, 5.3 | RaceEthn | Which one or more of the following would you say is your race? Are you American Indian or Alaska Native, Asian, Black or African American, Native Hawaiian or other Pacific Islander, or White? Are you Hispanic or Latino? (HD08-HD09 Recode for White (x = 0) compared to non-White (x = 1)) | Race and ethnicity, being White, being Black, being African American, being Hispanic, being Asian, being Pacific Islander, being Haitian, being Native, being Other |
|  | Gender | 3.1, 3.2, 4.2, 4.4, 5.1, 5.3 | GenderN, GenderC | Are you male or female? (GenderN Recode for Female (x = 0) compared to Male (x = 1))? | Gender, being male, being  female |
|  |  |  |  |  |  |
|  |  |  |  |  |  |
|  |  |  |  |  |  |

**eTable 1.** Meta-analytic constructs for the predictors of medical information avoidance with details on HINTS coverage. 3.1 = HINTS 2007 Dataset, updated February 2009; 3.2 = HINTS Puerto Rico 2009; 4.2 = HINTS 4, Cycle 2 (2012) dataset, updated October 2020; 4.4 = HINTS 4, Cycle 4 (2014) dataset, updated June 2021; 5.1 = HINTS 5, Cycle 1 (2017) dataset, updated May 2024; 5.3 = HINTS 5, Cycle 3 (2019) dataset, updated May 2024.

**eAppendix 6. Robustness check for meta-analytic predictors**

|  |  |  |  |  |  |  |  | 95% CI | | |  | Heterogeneity | |  |
| --- | --- | --- | --- | --- | --- | --- | --- | --- | --- | --- | --- | --- | --- | --- |
|  | Predictor | k | N | z̄ | z (range) | ES | SE |  | low | high |  | Q | I^2^ | fsn |
|  |  |  |  |  |  |  |  |  |  |  |  |  |  |  |
|  | **Cognitive Processing Factors** |  |  |  |  |  |  |  |  |  |  |  |  |  |
|  | Information overload | 12 | 26573 | 0.27 | [0.12, 0.52] | 0.27*** | 0.04 |  | 0.19 | 0.34 |  | 265.41 | 97.02 | 97 |
|  | Processing resources | 10 | 6350 | −0.21 | [−0.42, −0.06] | −0.21*** | 0.04 |  | −0.29 | −0.12 |  | 108.72 | 89.48 | 32 |
|  | **Cognitive-Emotional Factors** |  |  |  |  |  |  |  |  |  |  |  |  |  |
|  | Perceived stigma | 3 | 1782 | 0.38 | [0.22, 0.48] | 0.38*** | 0.08 |  | 0.23 | 0.53 |  | 13.05 | 88.54 | 4 |
|  | Self-efficacy | 12 | 28099 | −0.30 | [−0.51, −0.10] | −0.29*** | 0.04 |  | −0.38 | −0.21 |  | 530.89 | 97.74 | 96 |
|  | Trust | 13 | 26978 | −0.27 | [−0.94, 0.01] | −0.27*** | 0.07 |  | −0.40 | −0.14 |  | 480.94 | 99.08 | 31 |
|  | Fatalism | 12 | 28221 | 0.21 | [0.03, 0.34] | 0.21*** | 0.03 |  | 0.15 | 0.27 |  | 228.49 | 95.78 | 89 |
|  | Anxiety | 19 | 31784 | 0.15 | [−0.26, 0.81] | 0.15** | 0.05 |  | 0.04 | 0.25 |  | 1035.41 | 98.75 | 18 |
|  | Perceived threat | 19 | 32510 | 0.00 | [−0.45, 0.29] | 0.00 | 0.03 |  | −0.07 | 0.07 |  | 355.72 | 97.15 | 0 |
|  | **Health-Related Factors** |  |  |  |  |  |  |  |  |  |  |  |  |  |
|  | Health insurance status | 8 | 24428 | −0.13 | [−0.36, 0.09] | −0.13* | 0.06 |  | −0.24 | −0.02 |  | 458.70 | 98.50 | 4 |
|  | Health literacy | 13 | 18572 | −0.12 | [−0.27, 0.20] | −0.12*** | 0.03 |  | −0.19 | −0.06 |  | 140.30 | 94.27 | 25 |
|  | Medical history | 14 | 30701 | −0.12 | [−0.28, 0.13] | −0.12*** | 0.03 |  | −0.18 | −0.07 |  | 202.94 | 94.94 | 47 |
|  | Perceived health status | 13 | 27431 | −0.11 | [−0.24, 0.02] | −0.12*** | 0.02 |  | −0.16 | −0.08 |  | 126.48 | 88.63 | 62 |
|  | **Socio-Demographic Factors** |  |  |  |  |  |  |  |  |  |  |  |  |  |
|  | Employment | 8 | 11011 | −0.16 | [−0.39, −0.04] | −0.15*** | 0.03 |  | −0.21 | −0.10 |  | 36.98 | 85.74 | 27 |
|  | Education | 29 | 51340 | −0.10 | [−0.52, 0.22] | −0.10** | 0.03 |  | −0.17 | −0.03 |  | 1228.62 | 98.16 | 33 |
|  | Marital status | 16 | 26680 | −0.10 | [−0.42, 0.06] | −0.09*** | 0.03 |  | −0.15 | −0.04 |  | 200.21 | 94.76 | 25 |
|  | Income | 18 | 31768 | −0.09 | [−0.37, 0.23] | −0.09*** | 0.03 |  | −0.15 | −0.04 |  | 265.08 | 95.60 | 27 |
|  | Age | 43 | 448996 | −0.06 | [−0.43, 0.27] | −0.05* | 0.02 |  | −0.10 | −0.01 |  | 3001.01 | 98.70 | 19 |
|  | Gender | 28 | 64007 | 0.00 | [−0.20, 0.25] | 0.01 | 0.02 |  | −0.03 | 0.04 |  | 410.55 | 95.16 | 0 |
|  | Race and ethnicity | 15 | 421061 | 0.00 | [−0.25, 0.26] | 0.00 | 0.04 |  | −0.07 | 0.07 |  | 2367.02 | 99.22 | 0 |
|  |  |  |  |  |  |  |  |  |  |  |  |  |  |  |
|  |  |  |  |  |  |  |  |  |  |  |  |  |  |  |
|  |  |  |  |  |  |  |  |  |  |  |  |  |  |  |

**eTable 2.** Predictors of medical information avoidance, based on Fisher’s z transformation, with 95% confidence intervals (CIs). k = Number of effects; N = Aggregate sample size; z̄ = Mean effect size; ES = Estimated effect size; SE = Standard error of estimated effect size; Q = Cochran’s Q; fsn = Rosenthal’s fail-safe N. * *p* < 0.05; ** *p* < 0.01; *** *p* < 0.001.

**eAppendix 7. Forest plots for meta-analytic predictors**


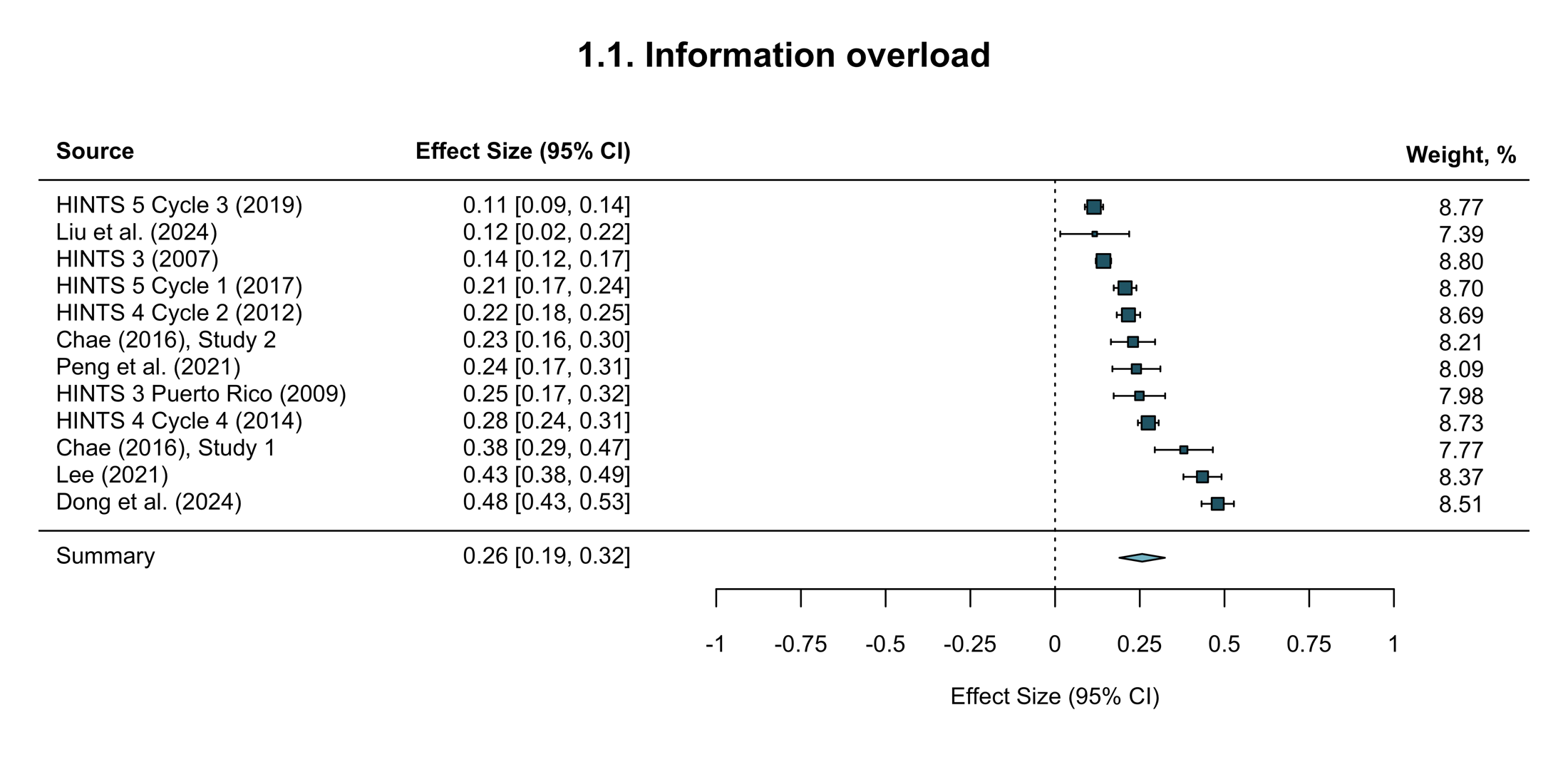


**eFigure 1.** Information overload as a predictor of medical information avoidance, based on an inverse-variance weighted random-effects model, with 95% confidence intervals (CIs).


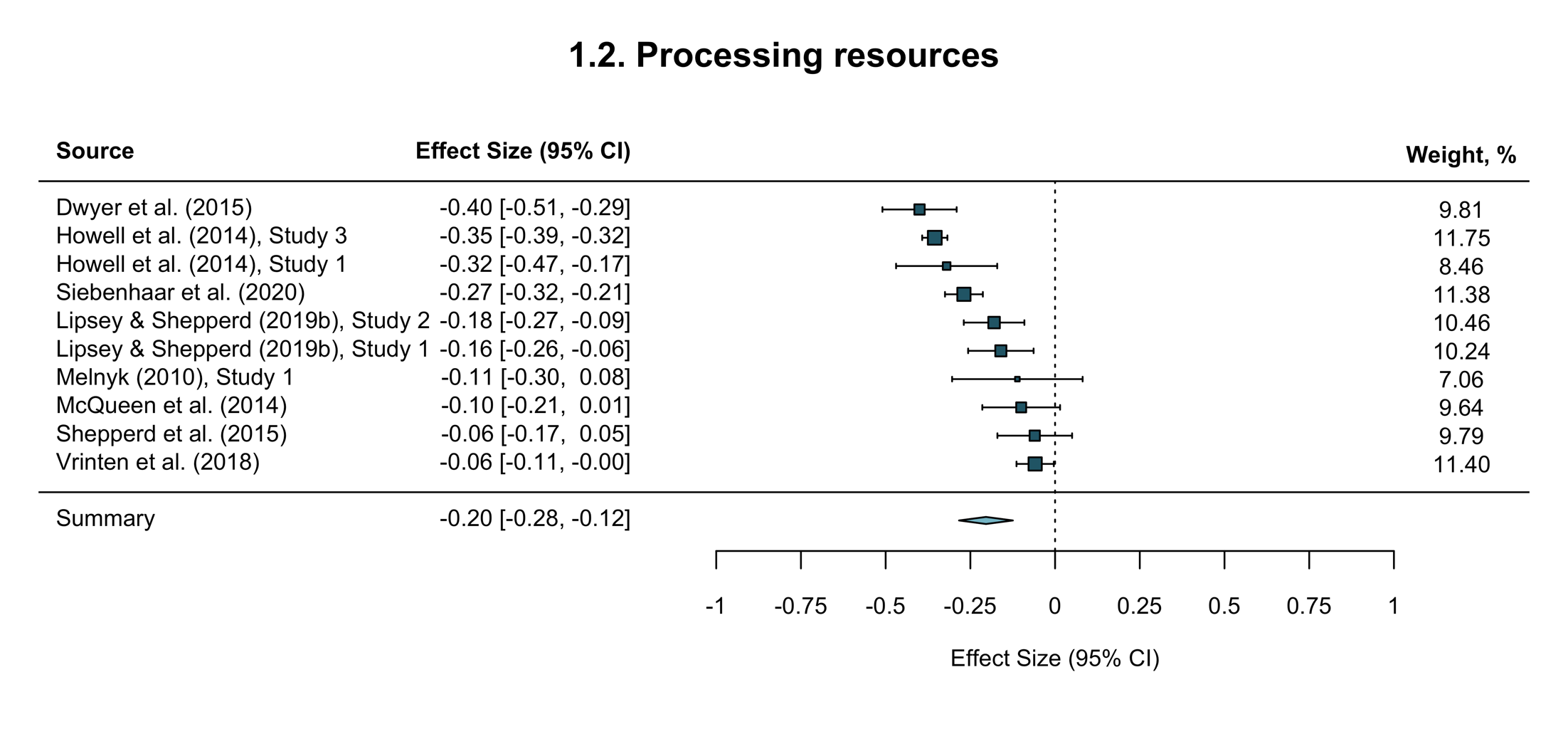


**eFigure 2.** Processing resources as a predictor of medical information avoidance, based on an inverse-variance weighted random-effects model, with 95% confidence intervals (CIs).


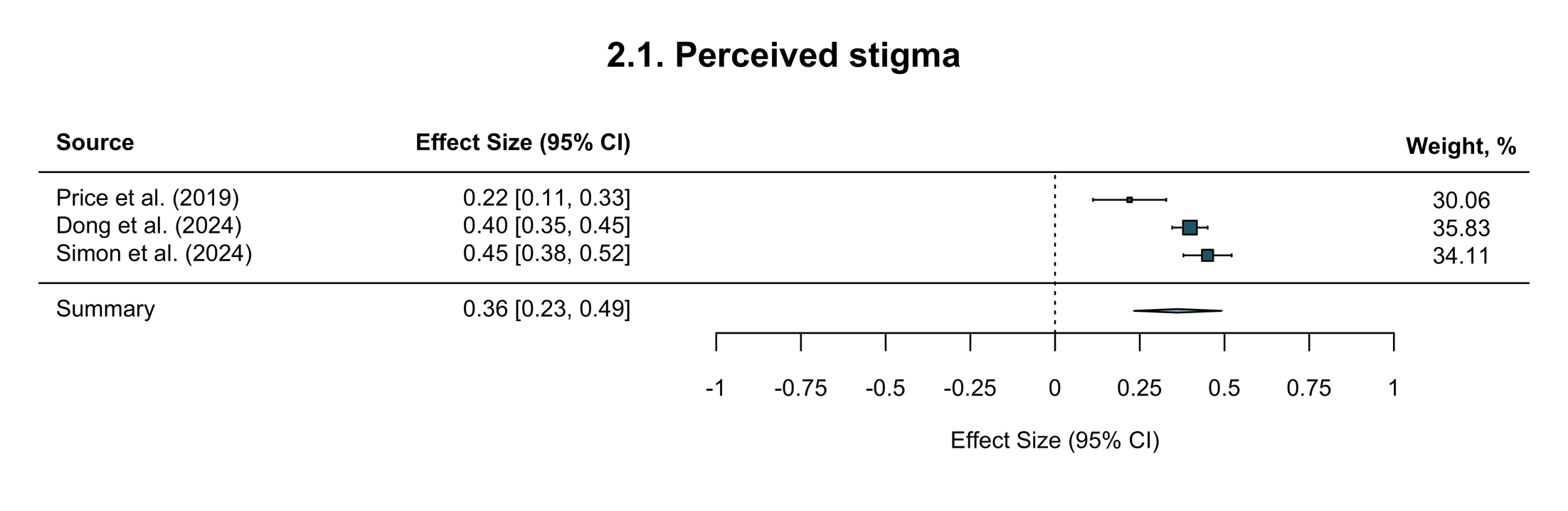


**eFigure 3.** Perceived stigma as a predictor of medical information avoidance, based on an inverse-variance weighted random-effects model, with 95% confidence intervals (CIs).


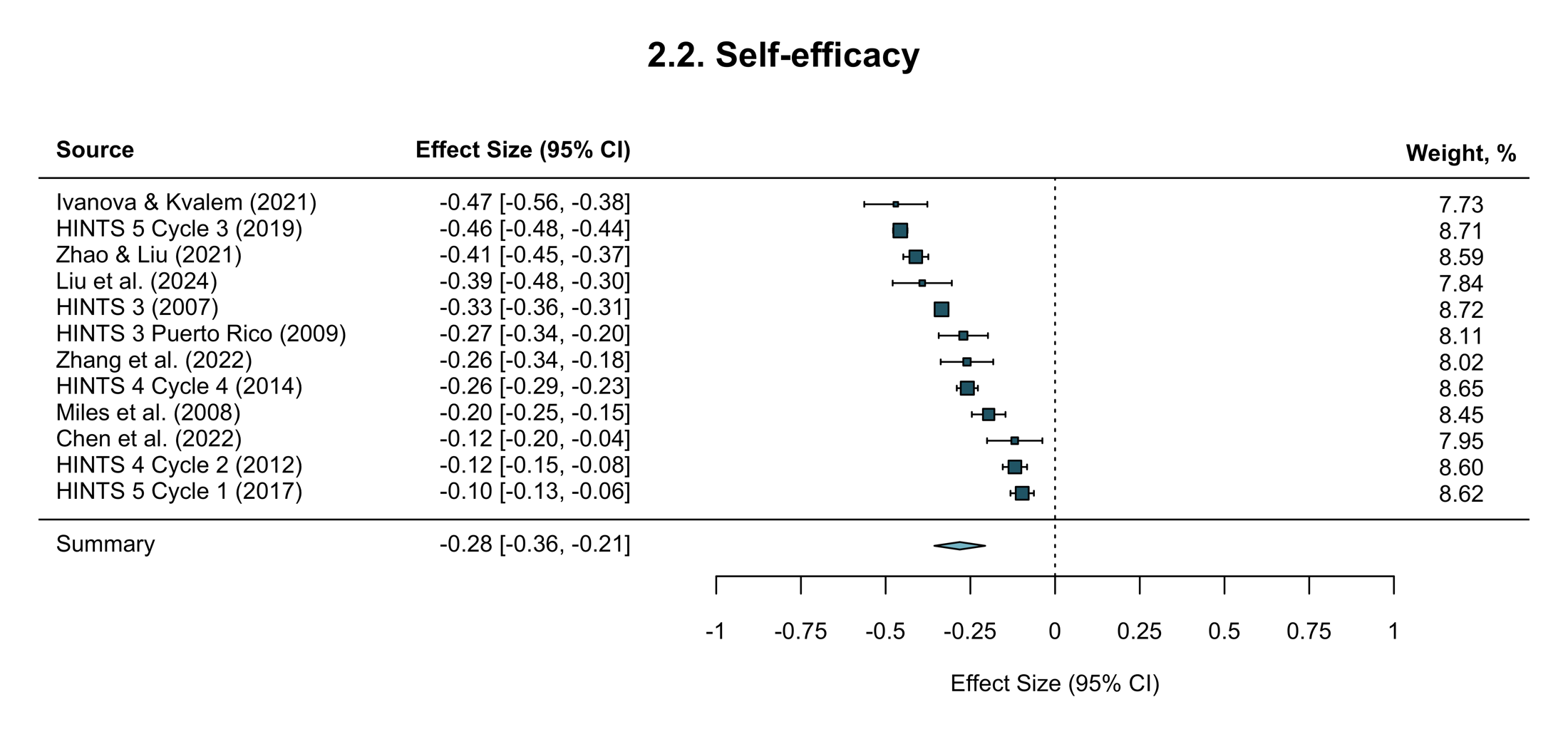


**eFigure 4.** Self-efficacy as a predictor of medical information avoidance, based on an inverse-variance weighted random-effects model, with 95% confidence intervals (CIs).


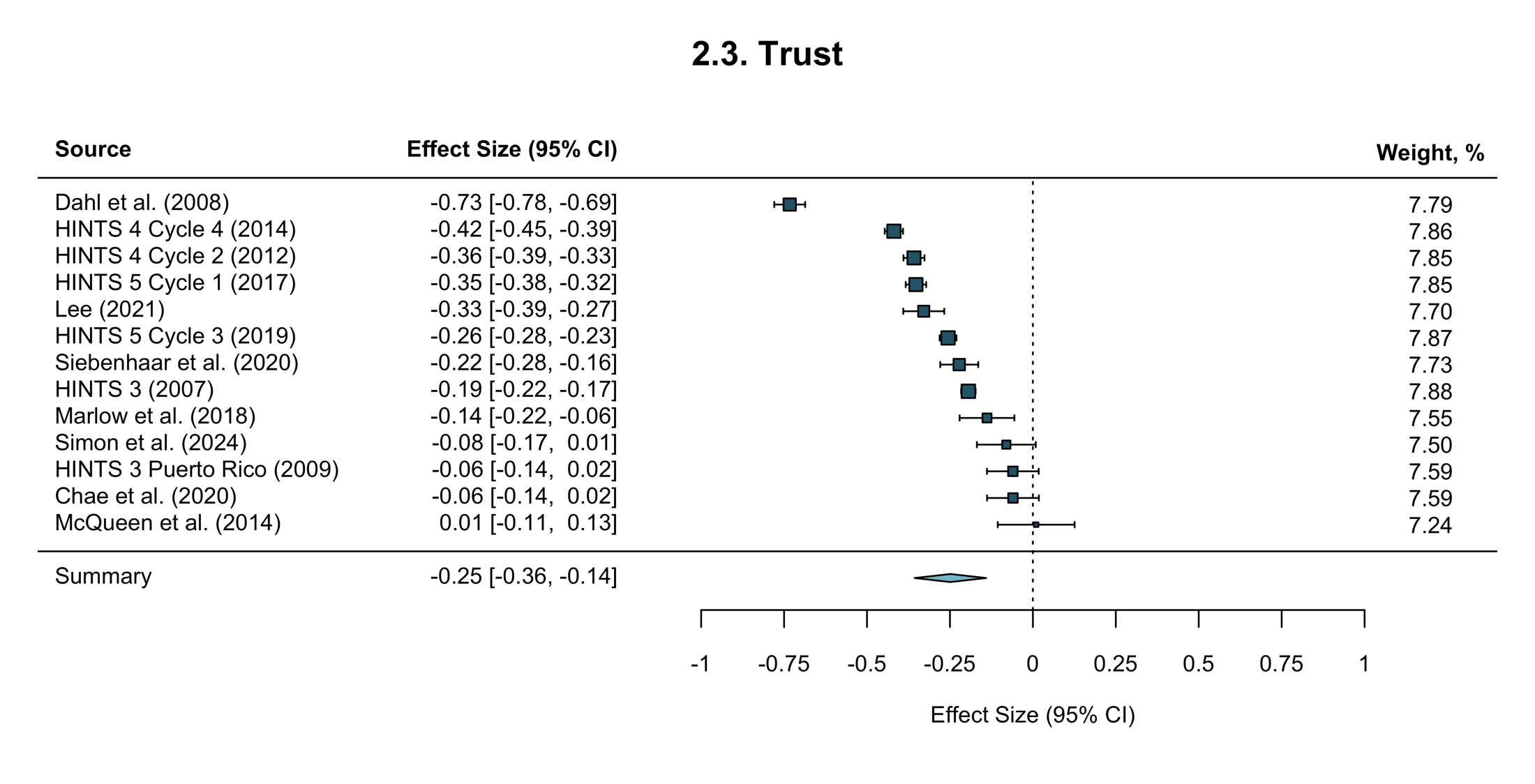


**eFigure 5.** Trust as a predictor of medical information avoidance, based on an inverse-variance weighted random-effects model, with 95% confidence intervals (*C*Is).


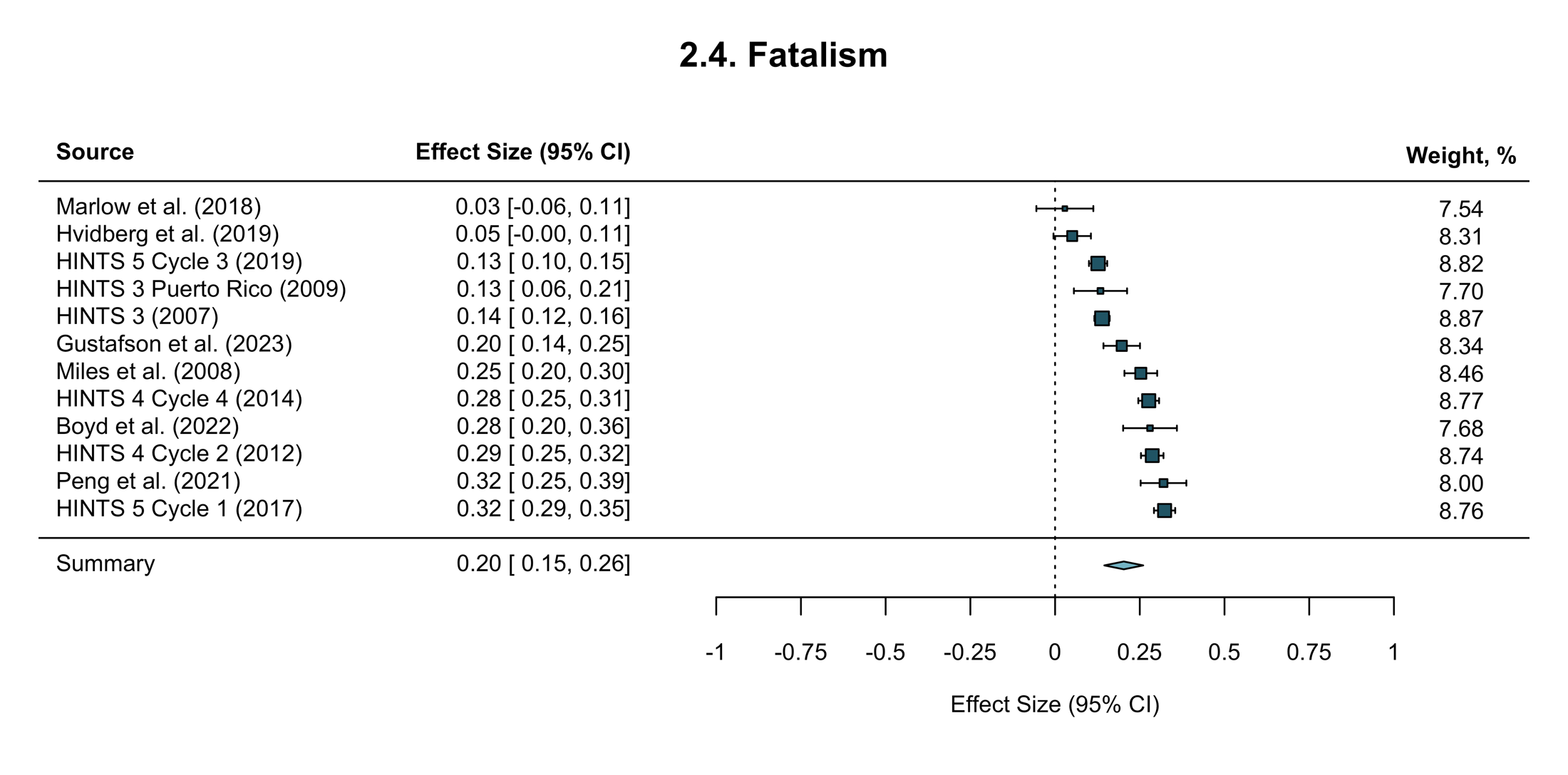


**eFigure 6.** Fatalism as a predictor of medical information avoidance, based on an inverse-variance weighted random-effects model, with 95% confidence intervals (CIs).


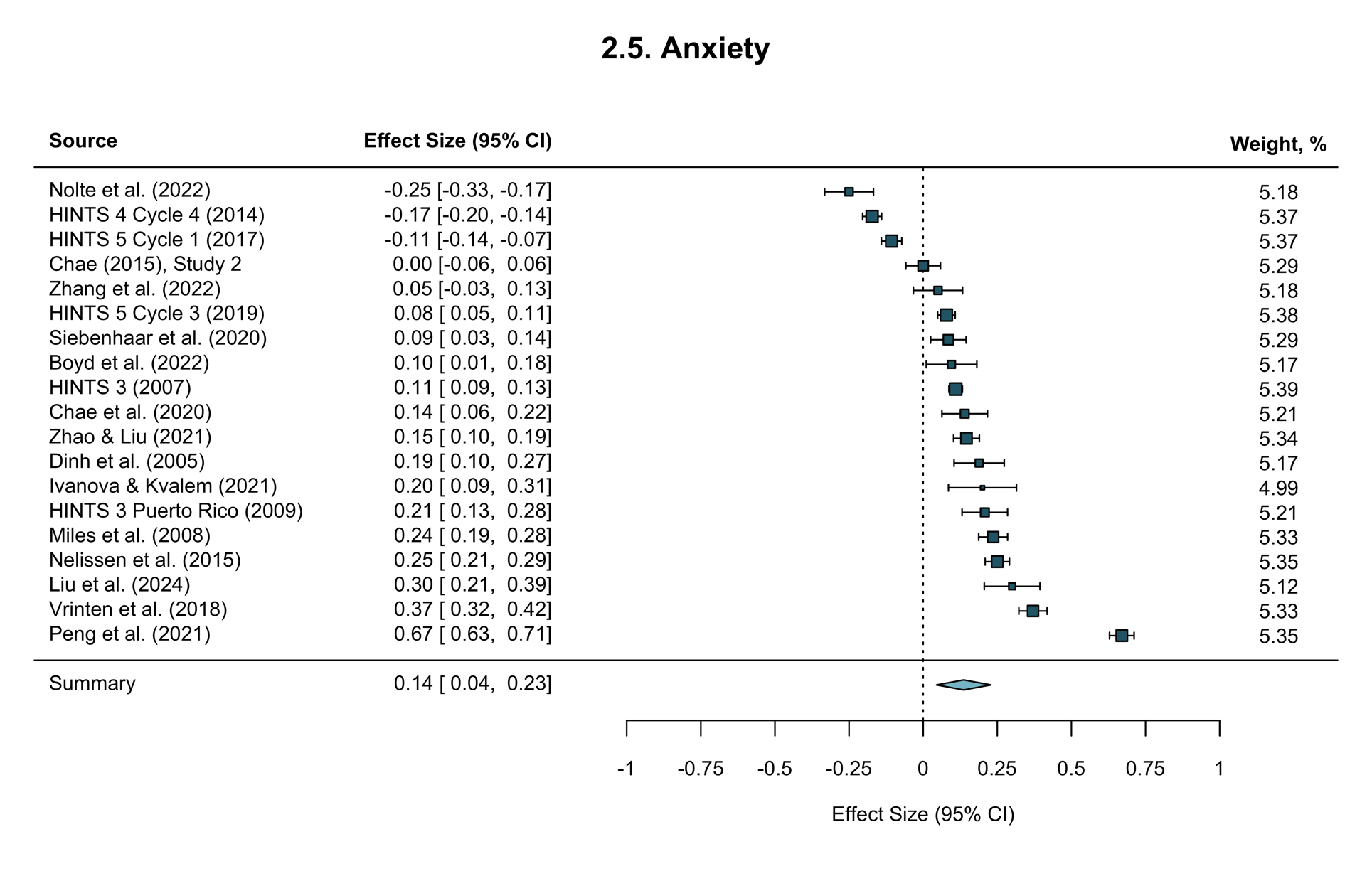


**eFigure 7.** Anxiety as a predictor of medical information avoidance, based on an inverse-variance weighted random-effects model, with 95% confidence intervals (CIs).


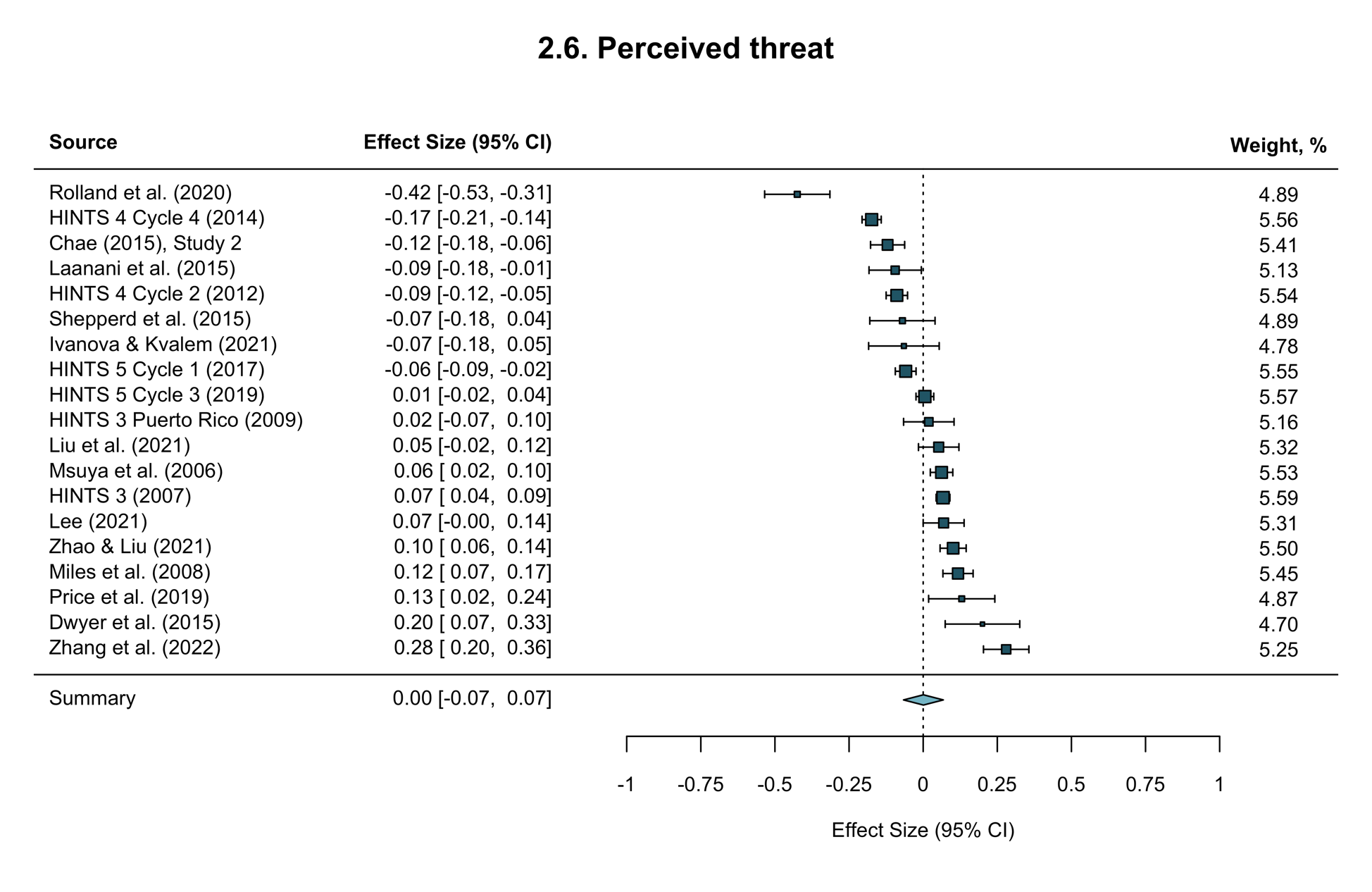


**eFigure 8.** Perceived threat as a predictor of medical information avoidance, based on an inverse-variance weighted random-effects model, with 95% confidence intervals (CIs).


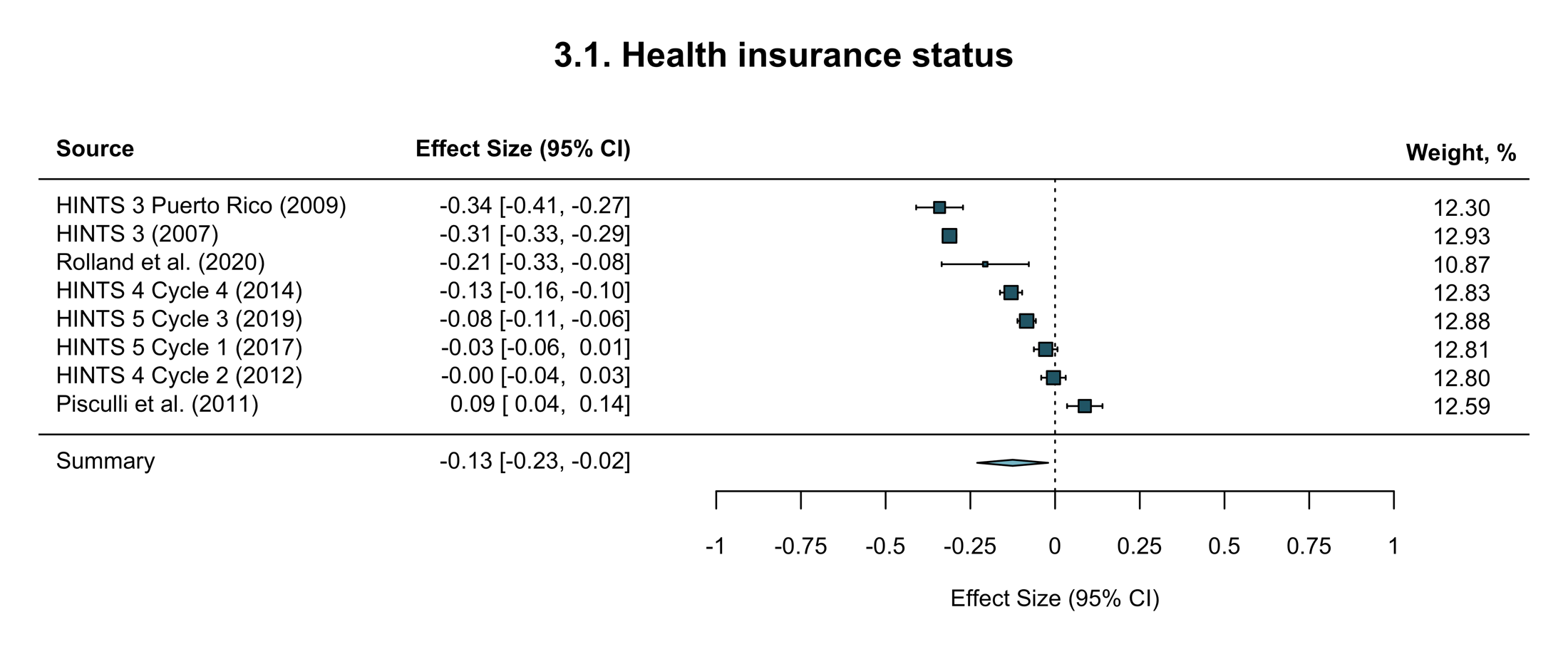


**eFigure 9.** Health insurance status as a predictor of medical information avoidance, based on an inverse-variance weighted random-effects model, with 95% confidence intervals (CIs).


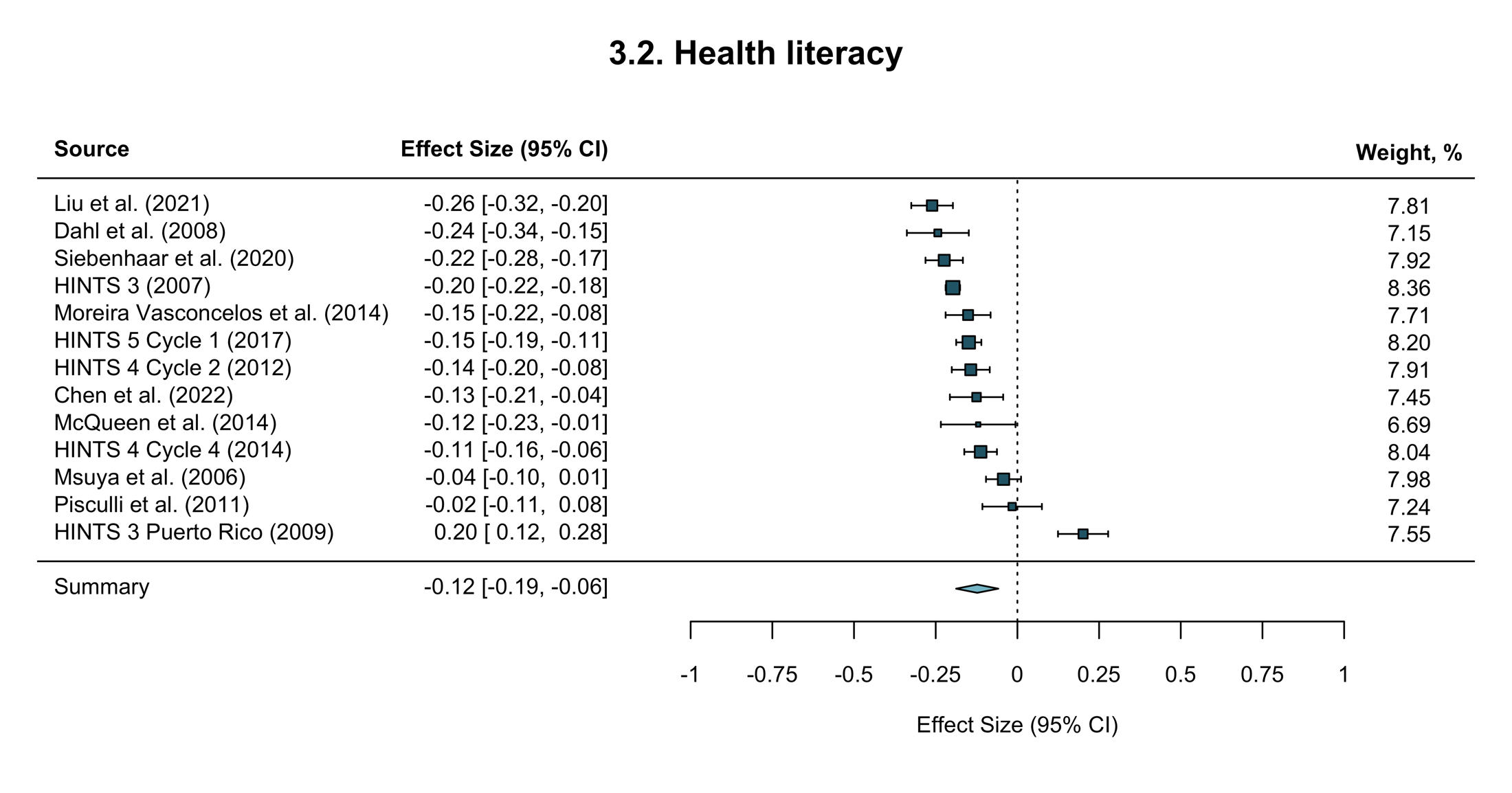


**eFigure 10.** Health literacy as a predictor of medical information avoidance, based on an inverse-variance weighted random-effects model, with 95% confidence intervals (CIs).


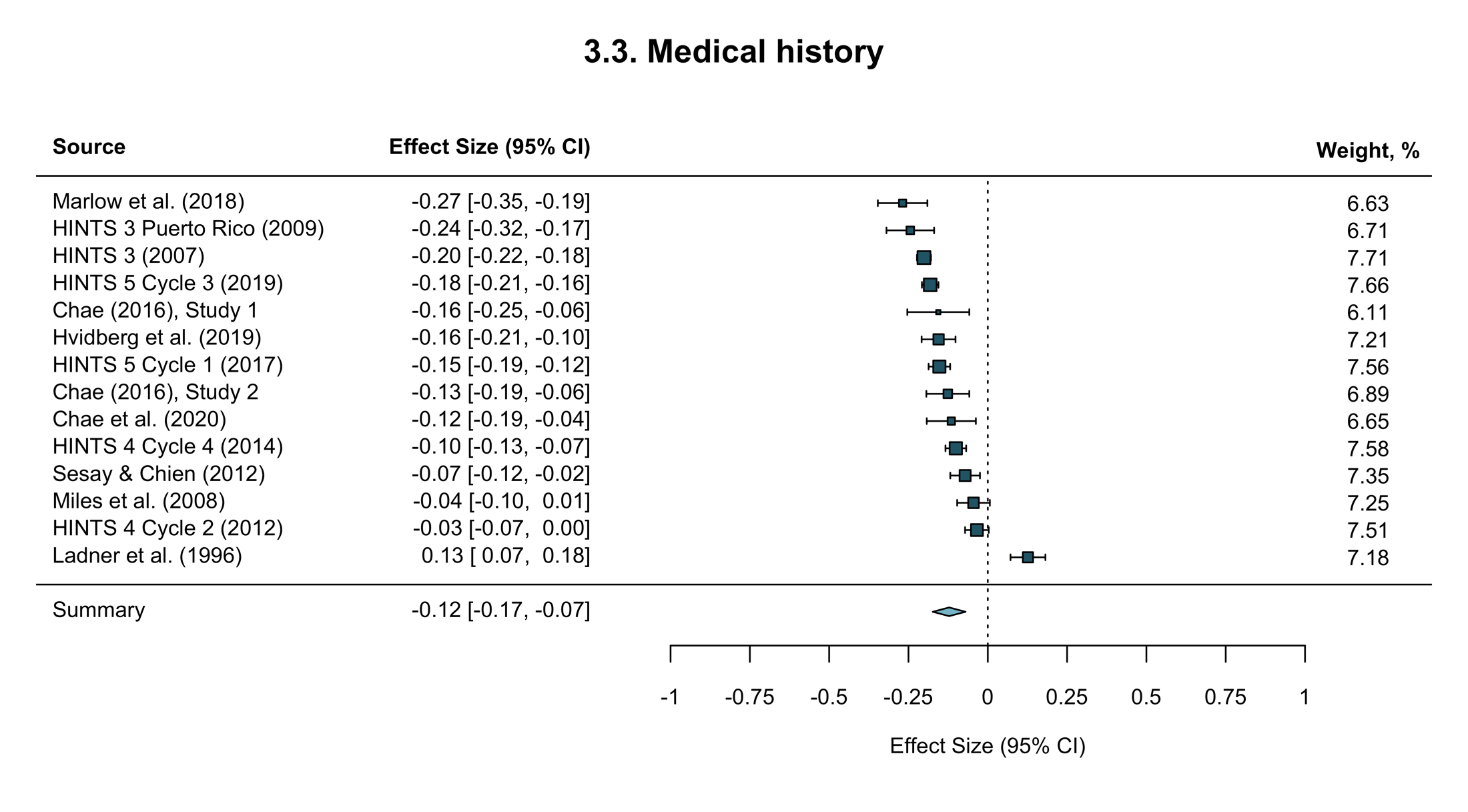


**eFigure 11.** Medical history as a predictor of medical information avoidance, based on an inverse-variance weighted random-effects model, with 95% confidence intervals (CIs).


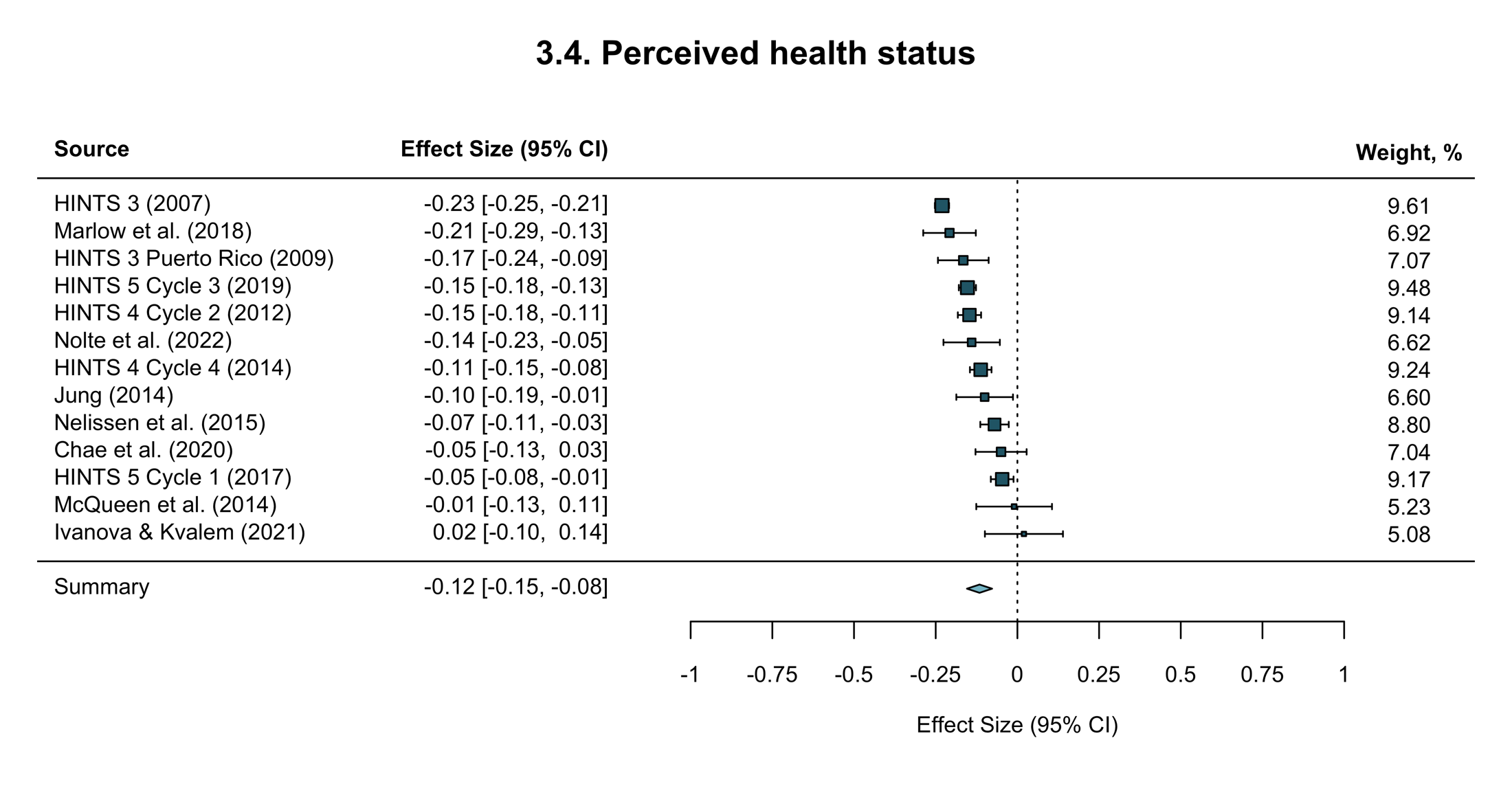


**eFigure 12.** Perceived health status as a predictor of medical information avoidance, based on an inverse-variance weighted random-effects model, with 95% confidence intervals (CIs).


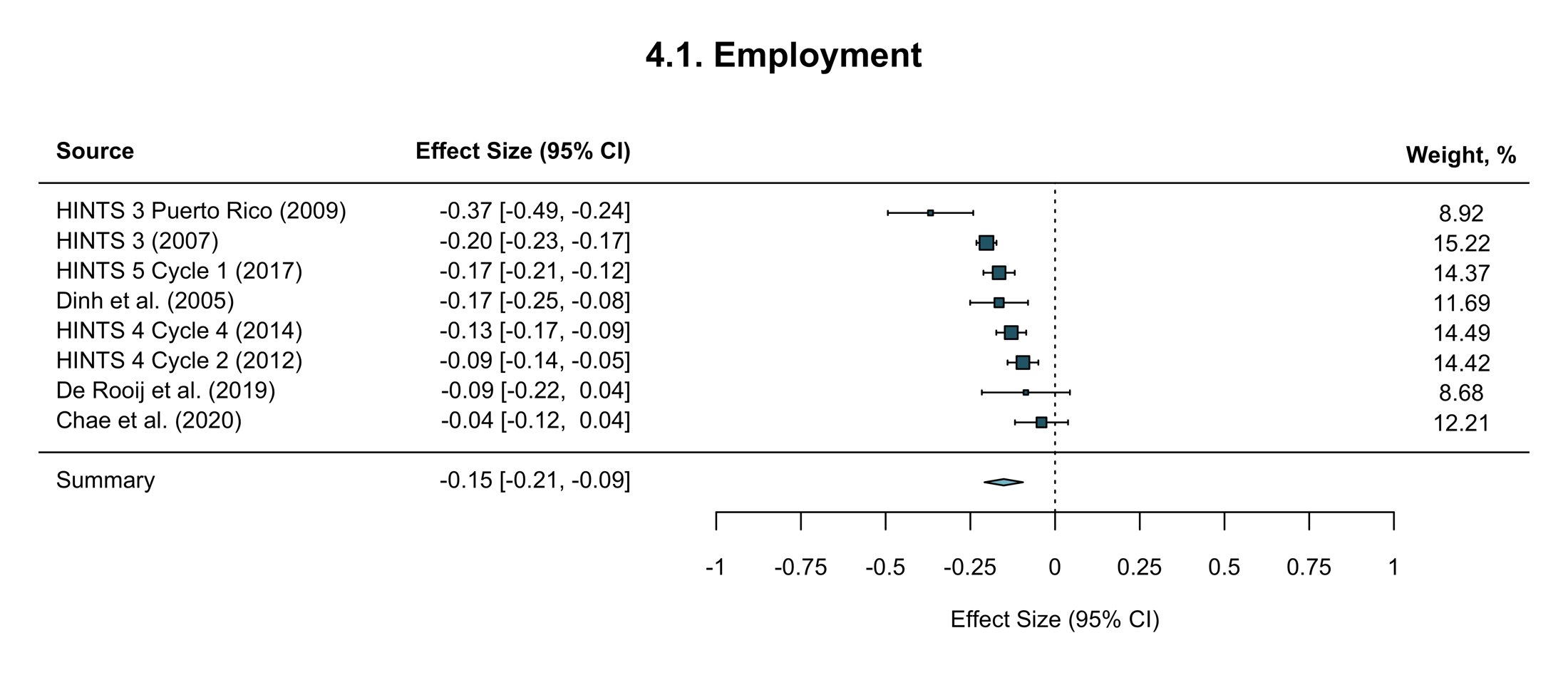


**eFigure 13.** Employment as a predictor of medical information avoidance, based on an inverse-variance weighted random-effects model, with 95% confidence intervals (CIs).


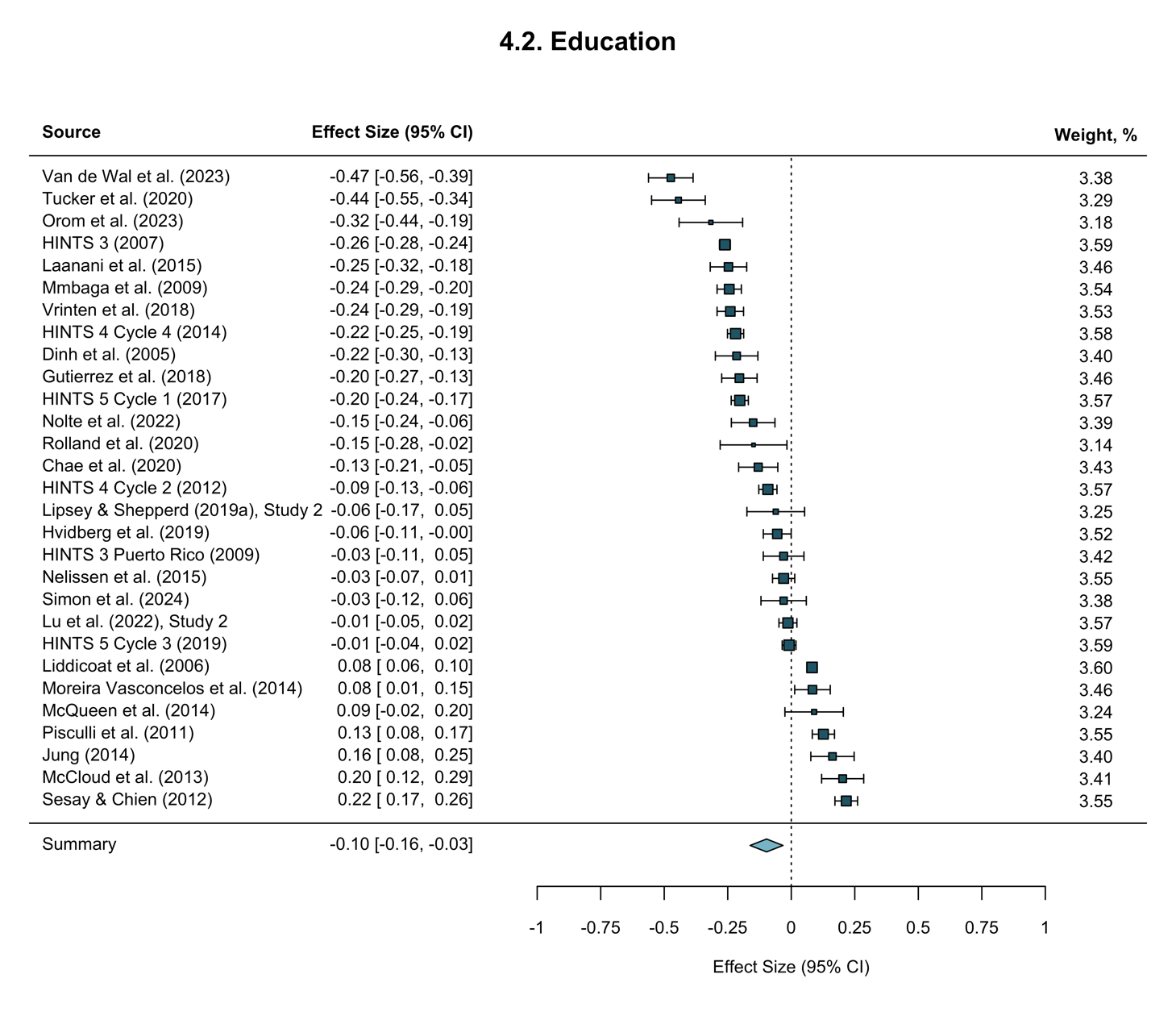


**eFigure 14.** Education as a predictor of medical information avoidance, based on an inverse-variance weighted random-effects model, with 95% confidence intervals (CIs).


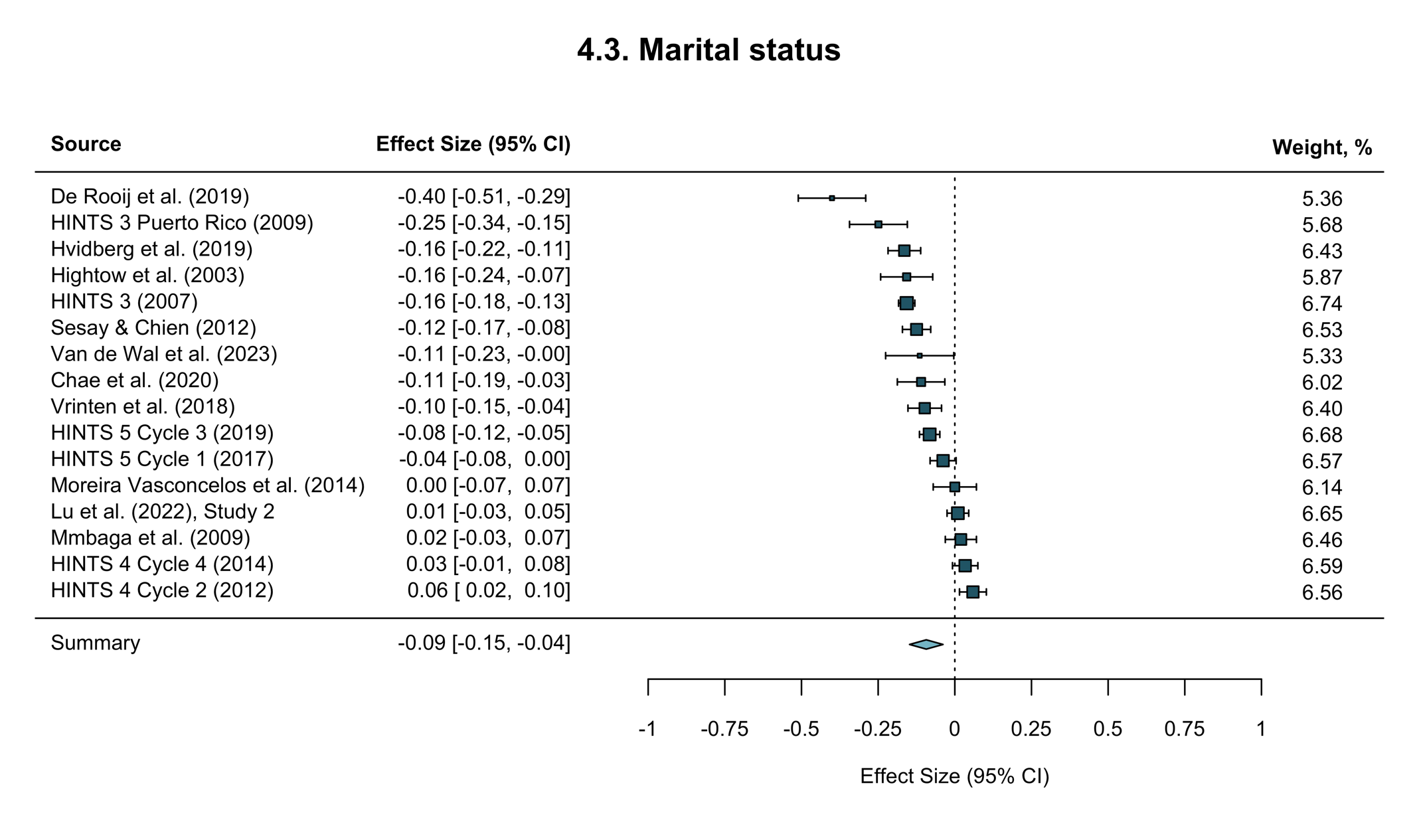


**eFigure 15.** Marital status as a predictor of medical information avoidance, based on an inverse-variance weighted random-effects model, with 95% confidence intervals (CIs).


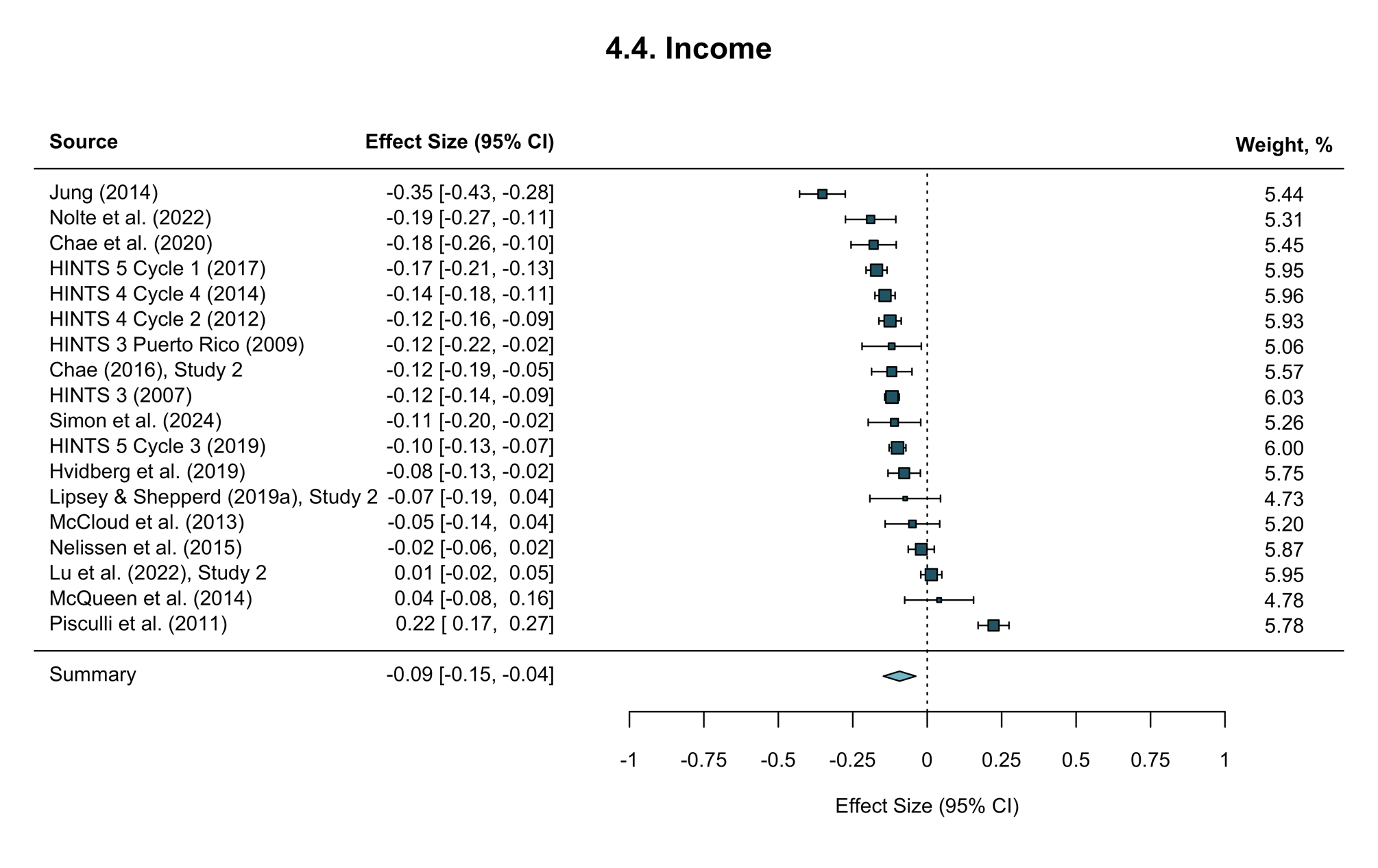


**eFigure 16.** Income as a predictor of medical information avoidance, based on an inverse-variance weighted random-effects model, with 95% confidence intervals (CIs).


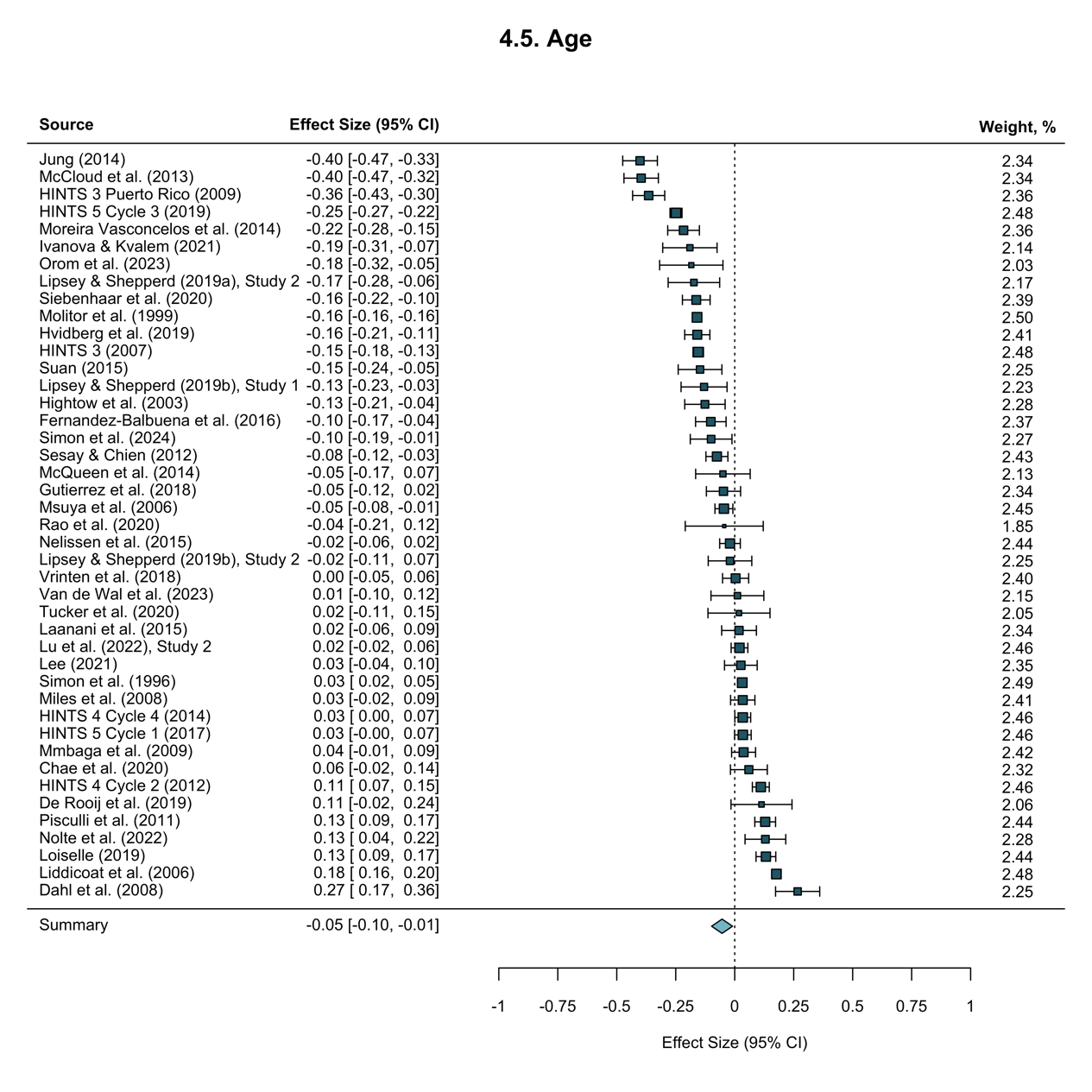


**eFigure 17.** Age as a predictor of medical information avoidance, based on an inverse-variance weighted random-effects model, with 95% confidence intervals (CIs).


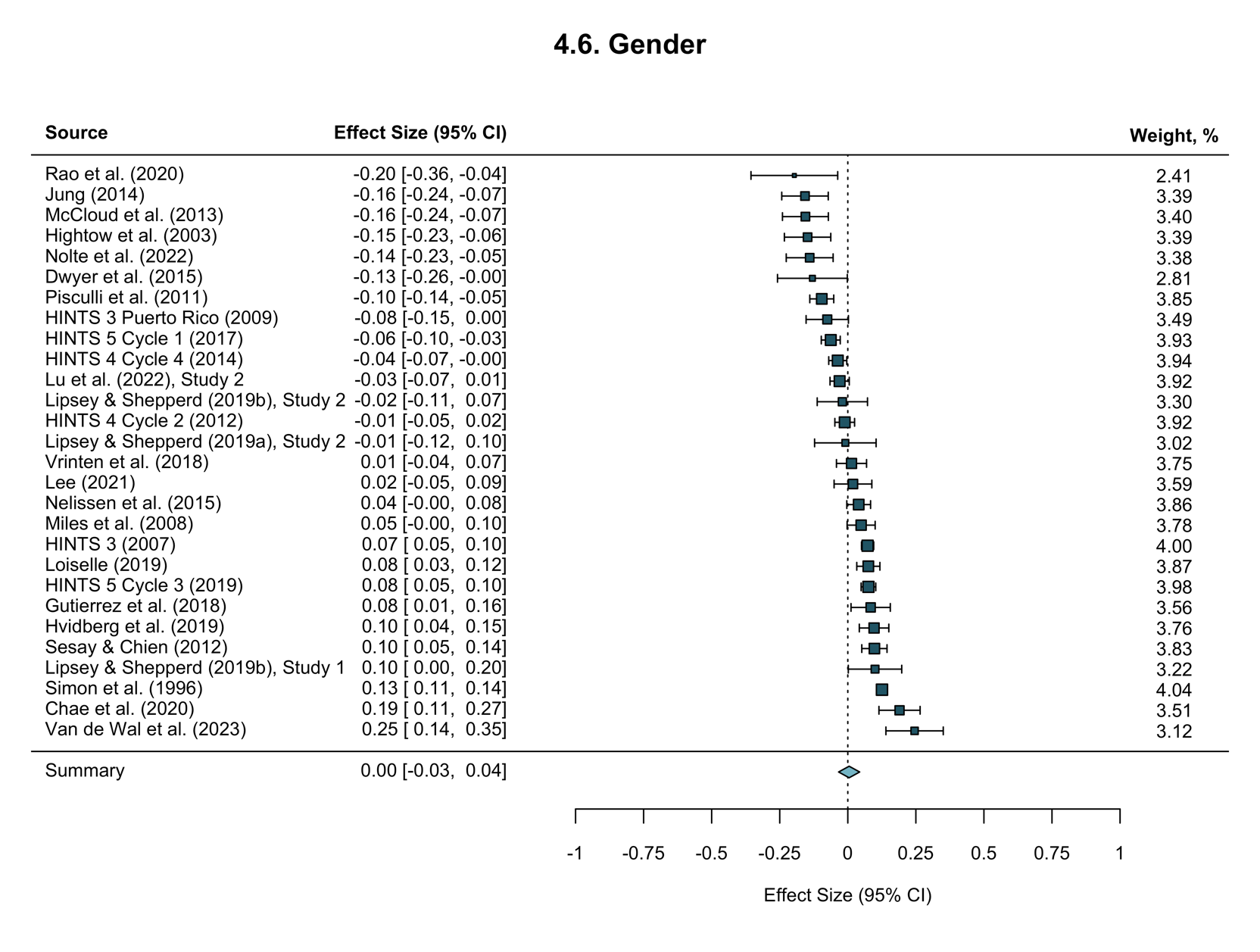


**eFigure 18.** Gender as a predictor of medical information avoidance, based on an inverse-variance weighted random-effects model, with 95% confidence intervals (CIs). To recap, gender is coded with females as x = 0 and males as x = 1, as specified in eAppendix 5.


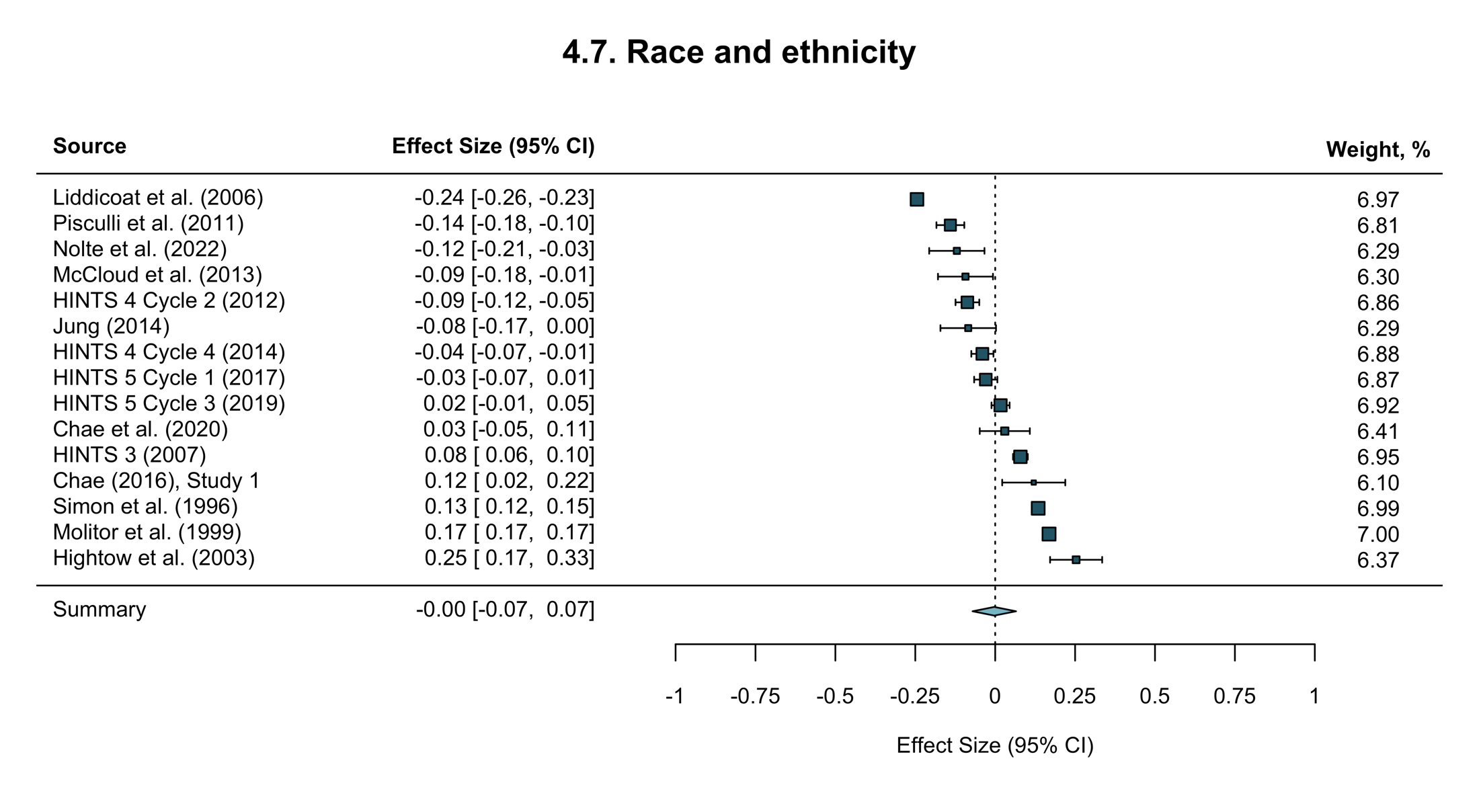


**eFigure 19.** Race and ethnicity as a predictor of medical information avoidance, based on an inverse-variance weighted random-effects model, with 95% confidence intervals (CIs). To recap, race and ethnicity are coded with Whites as x = 0 and non-Whites as x = 1, as specified in eAppendix 5.

**eAppendix 8. Funnel plots for meta-analytic predictors**


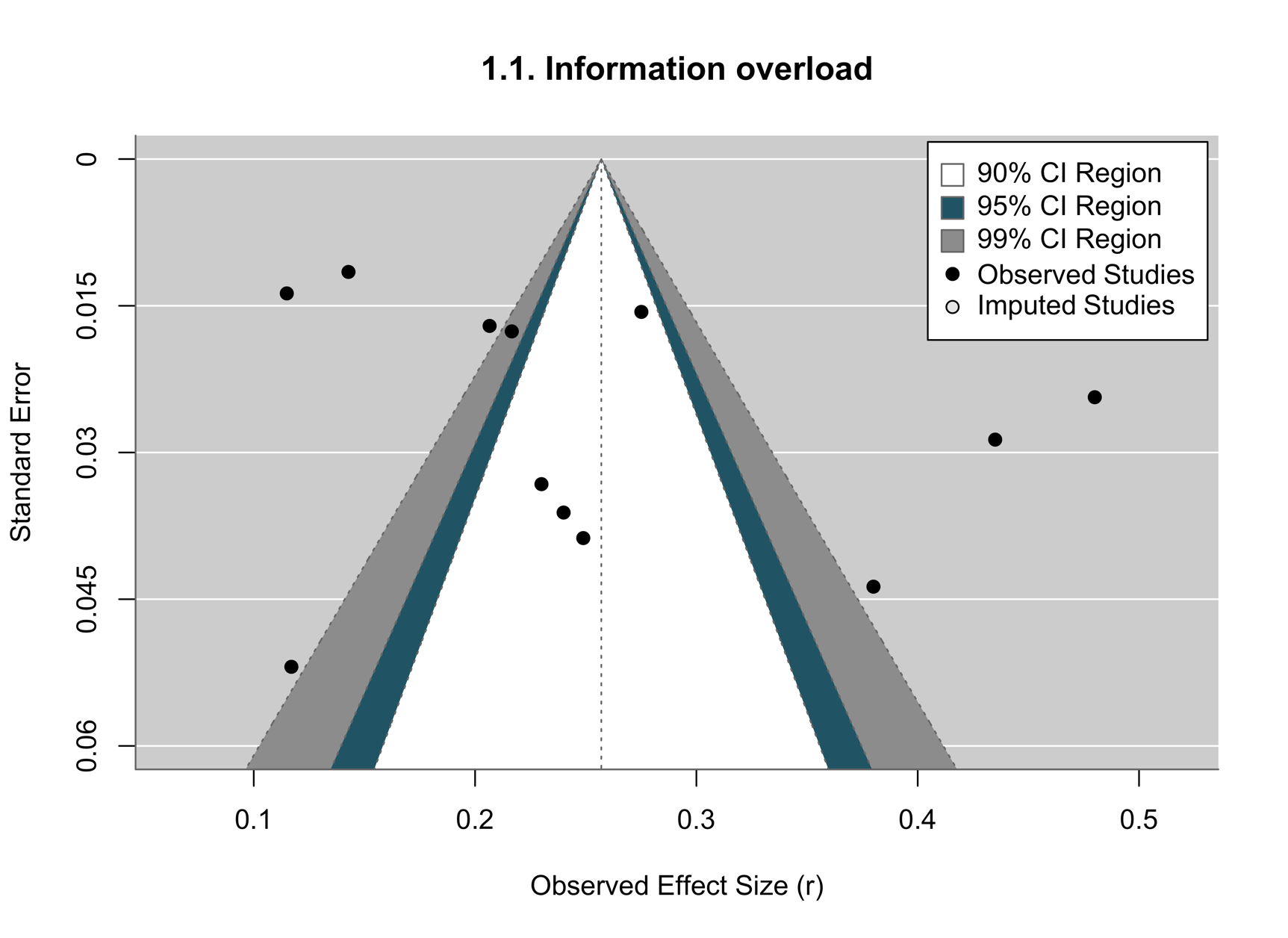


**eFigure 20.** Funnel plot for information overload as a predictor of medical information avoidance, after applying Duval and Tweedie’s trim and fill method, with 90%, 95% and 99% confidence interval (CI) regions.


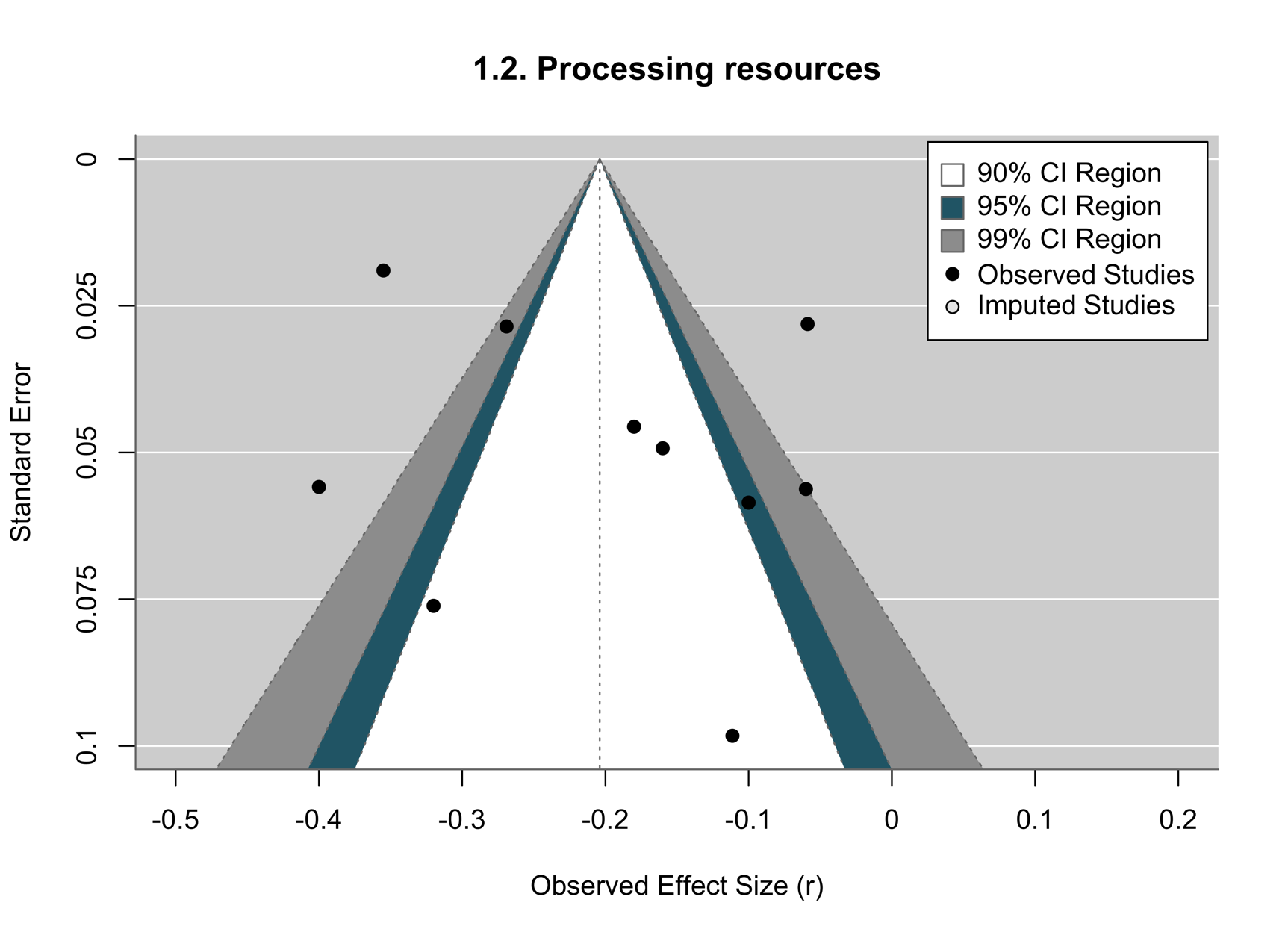


**eFigure 21.** Funnel plot for processing resources as a predictor of medical information avoidance, after applying Duval and Tweedie’s trim and fill method, with 90%, 95% and 99% confidence interval (CI) regions.


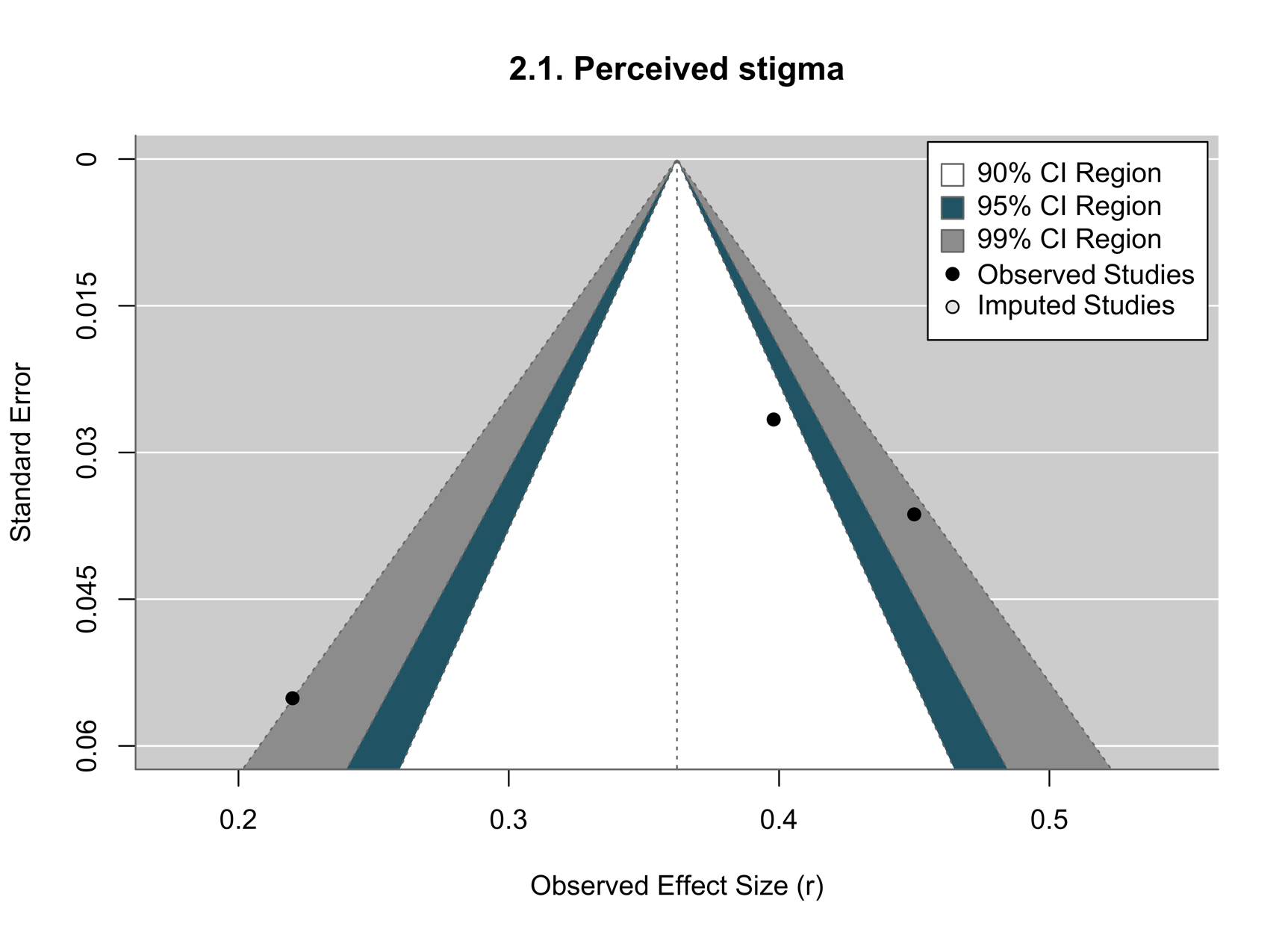


**eFigure 22.** Funnel plot for perceived stigma as a predictor of medical information avoidance, after applying Duval and Tweedie’s trim and fill method, with 90%, 95% and 99% confidence interval (CI) regions.


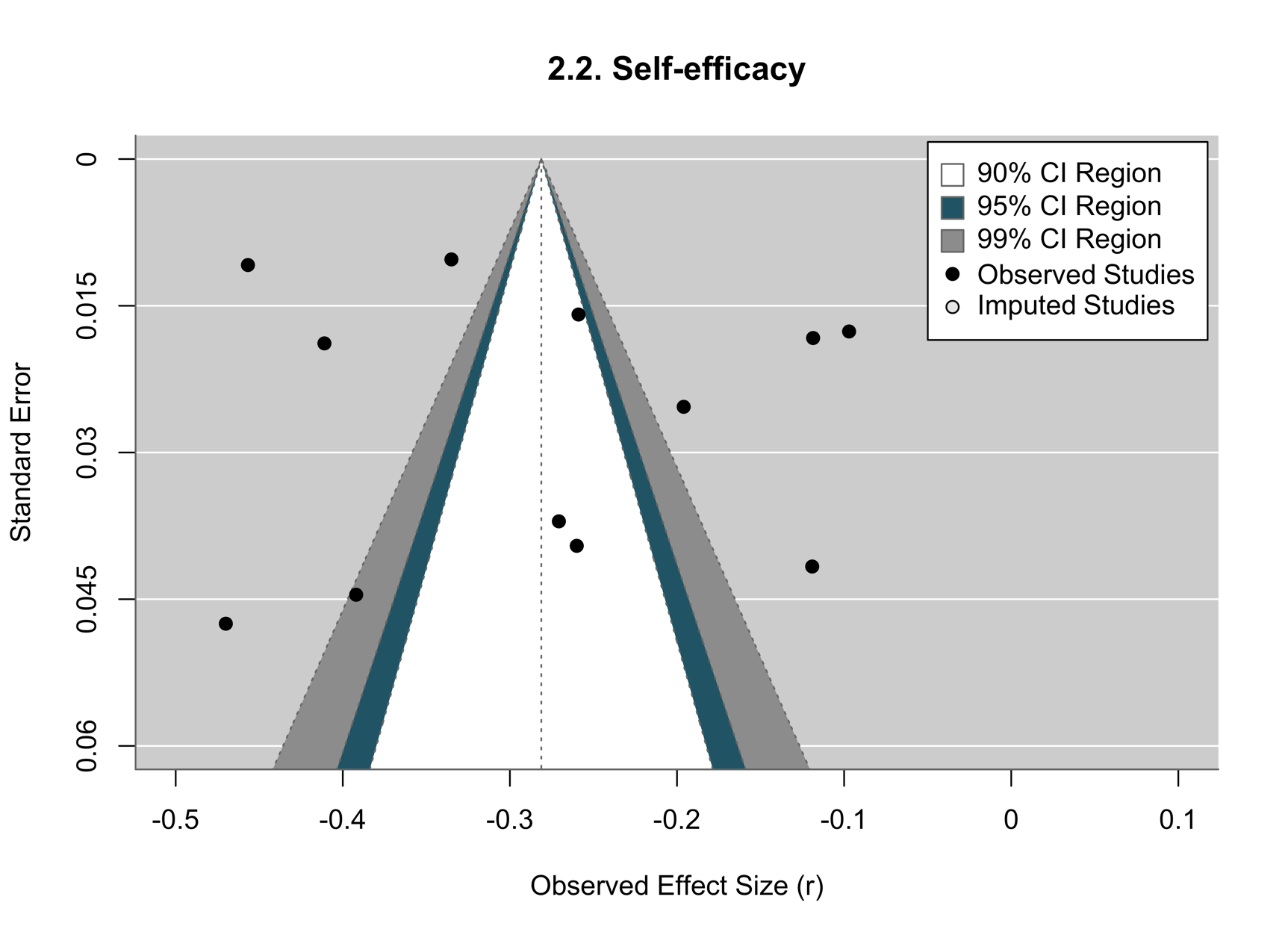


**eFigure 23.** Funnel plot for self-efficacy as a predictor of medical information avoidance, after applying Duval and Tweedie’s trim and fill method, with 90%, 95% and 99% confidence interval (CI) regions.


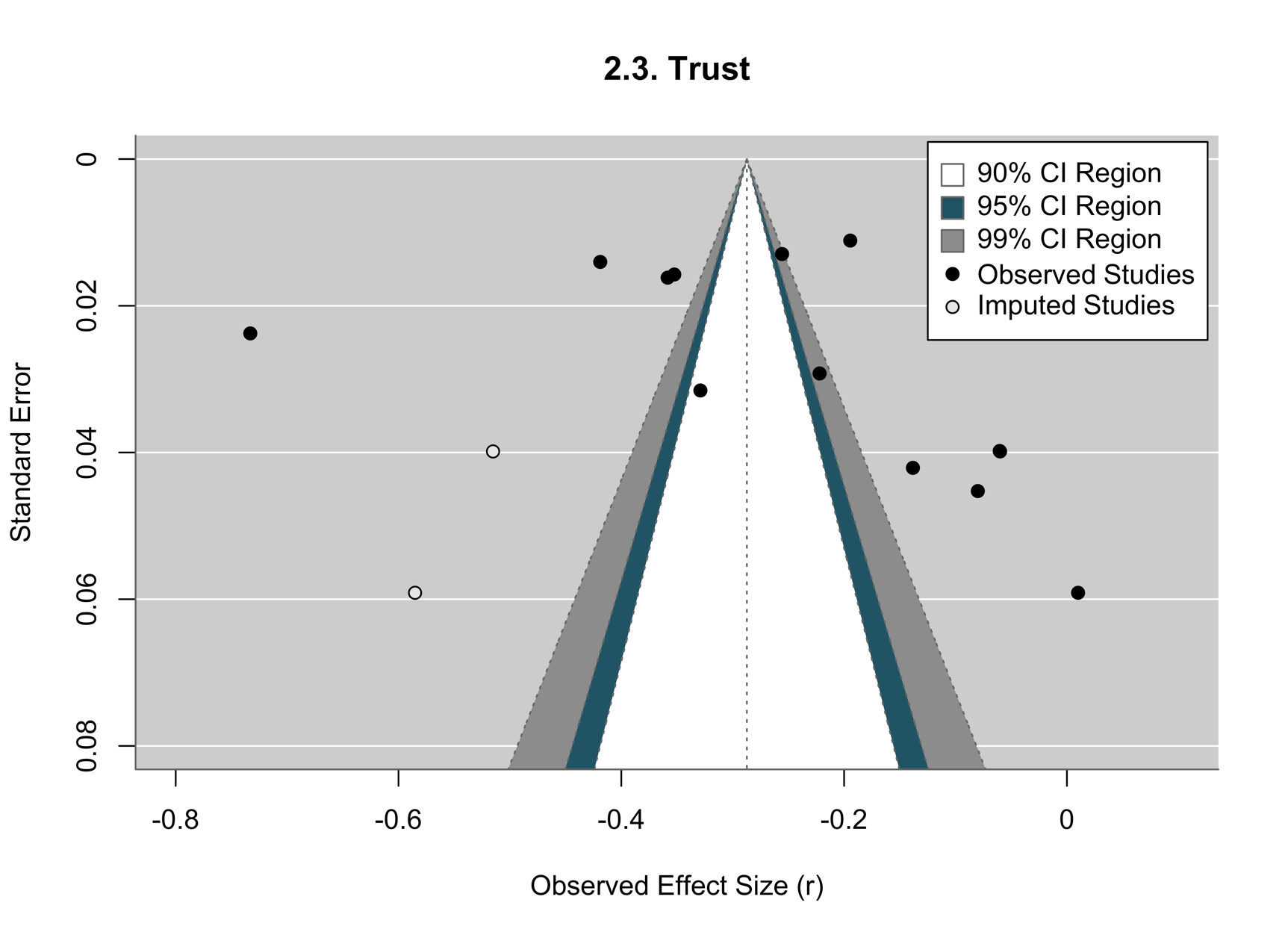


**eFigure 24.** Funnel plot for trust as a predictor of medical information avoidance, after applying Duval and Tweedie’s trim and fill method, with 90%, 95% and 99% confidence interval (CI) regions.


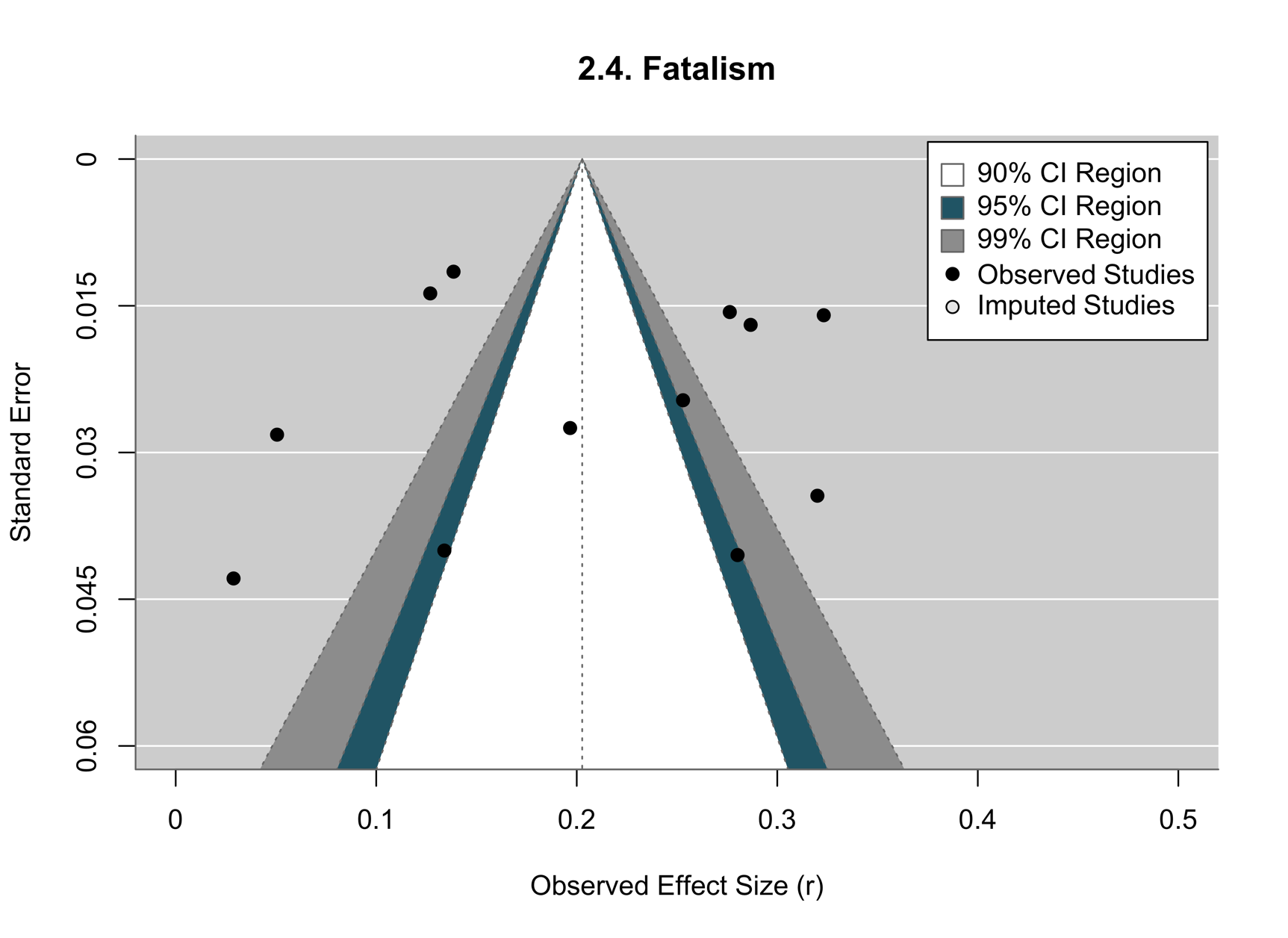


**eFigure 25.** Funnel plot for fatalism as a predictor of medical information avoidance, after applying Duval and Tweedie’s trim and fill method, with 90%, 95% and 99% confidence interval (CI) regions.


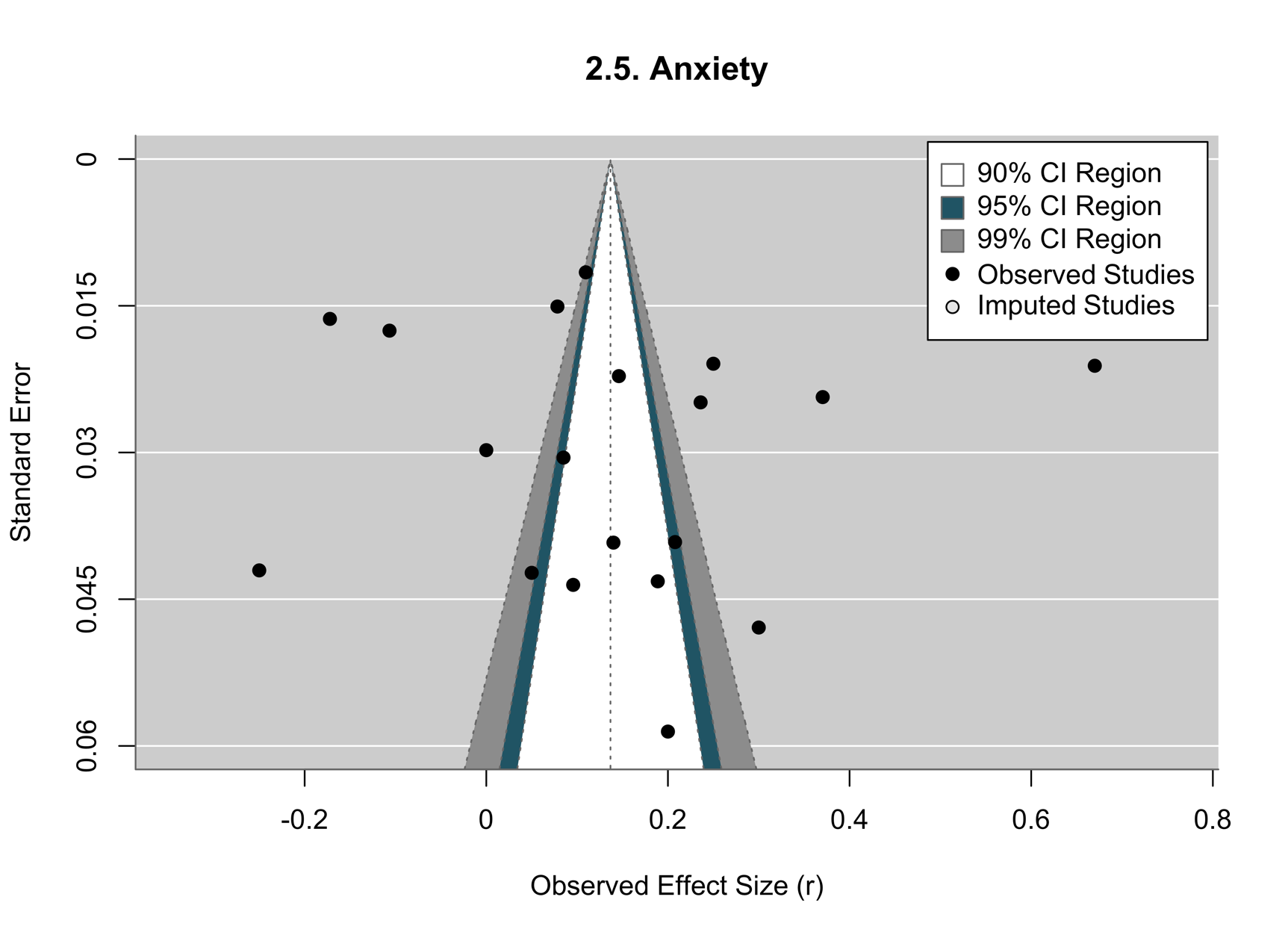


**eFigure 26.** Funnel plot for anxiety as a predictor of medical information avoidance, after applying Duval and Tweedie’s trim and fill method, with 90%, 95% and 99% confidence interval (CI) regions.


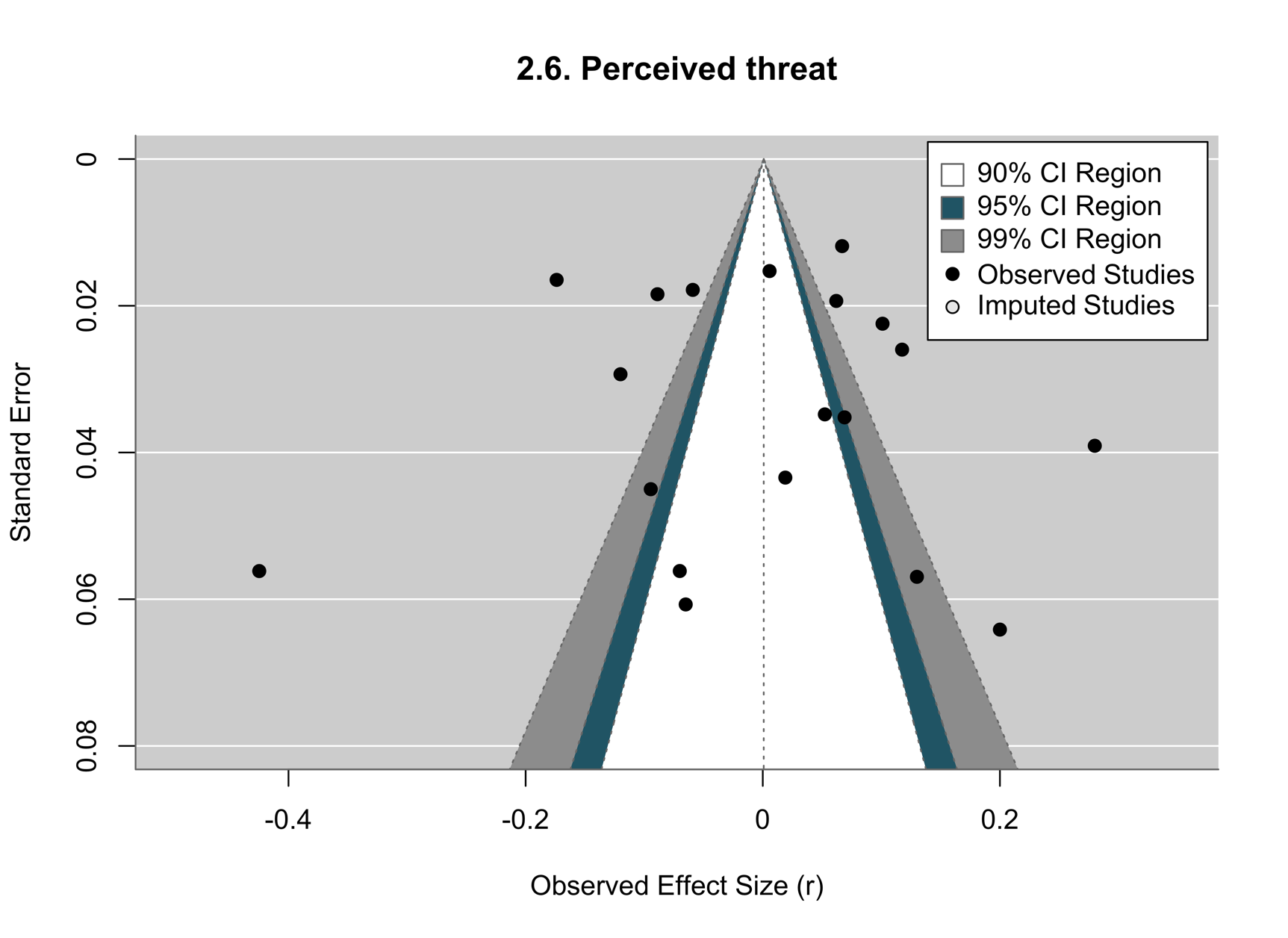


**eFigure 27.** Funnel plot for perceived threat as a predictor of medical information avoidance, after applying Duval and Tweedie’s trim and fill method, with 90%, 95% and 99% confidence interval (CI) regions.


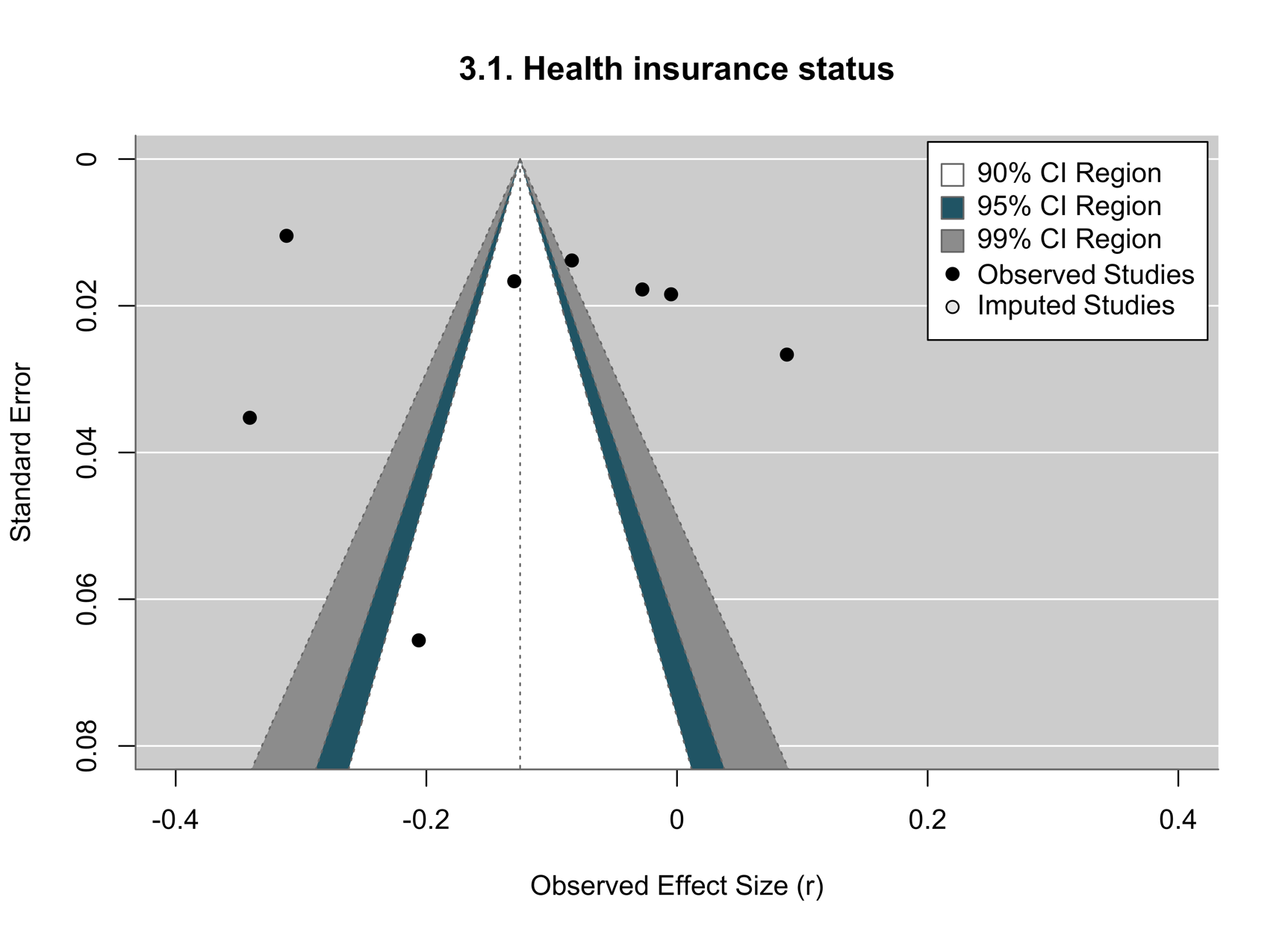


**eFigure 28.** Funnel plot for health insurance status as a predictor of medical information avoidance, after applying Duval and Tweedie’s trim and fill method, with 90%, 95% and 99% confidence interval (CI) regions.


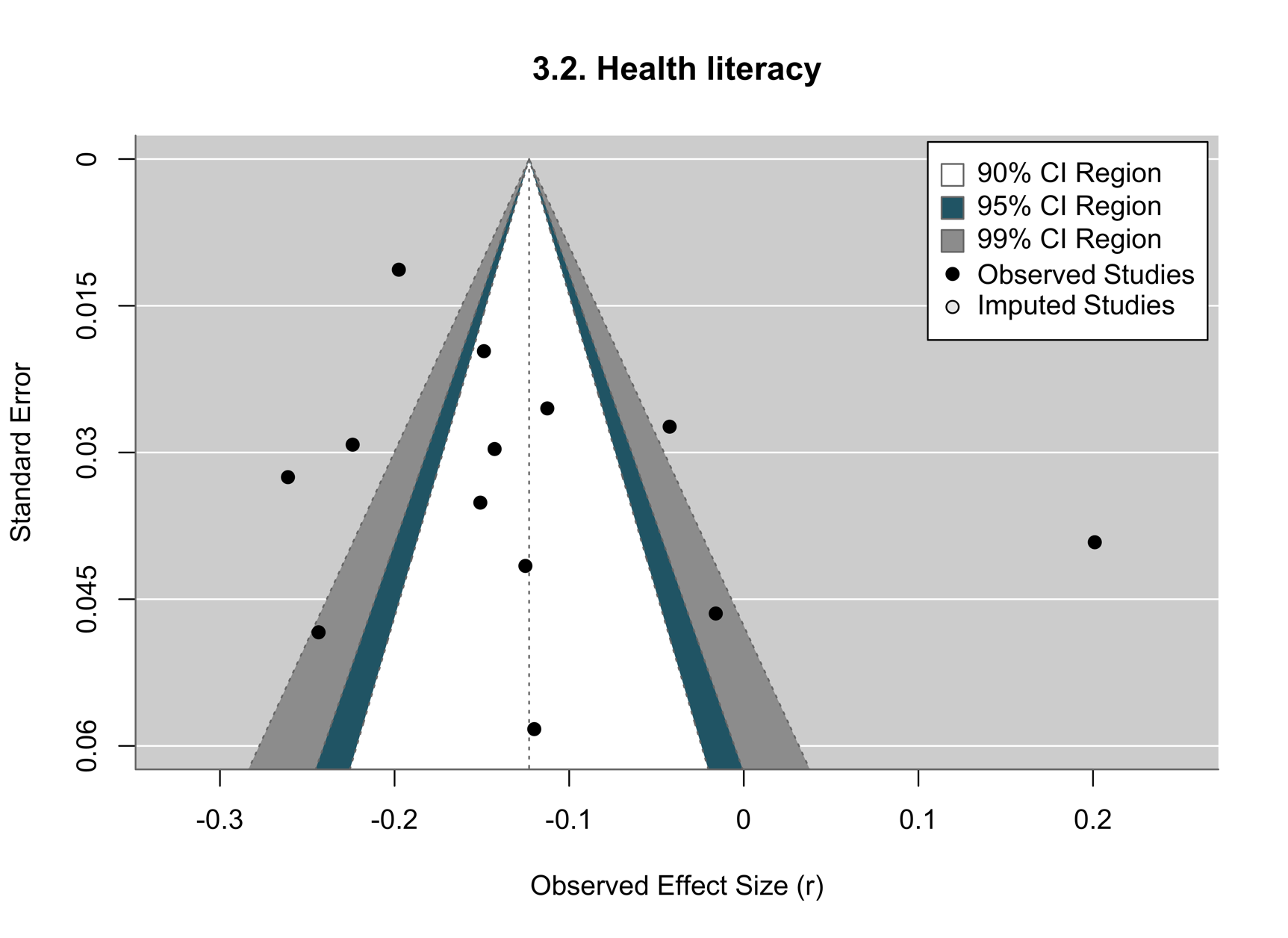


**eFigure 29.** Funnel plot for health literacy as a predictor of medical information avoidance, after applying Duval and Tweedie’s trim and fill method, with 90%, 95% and 99% confidence interval (CI) regions.


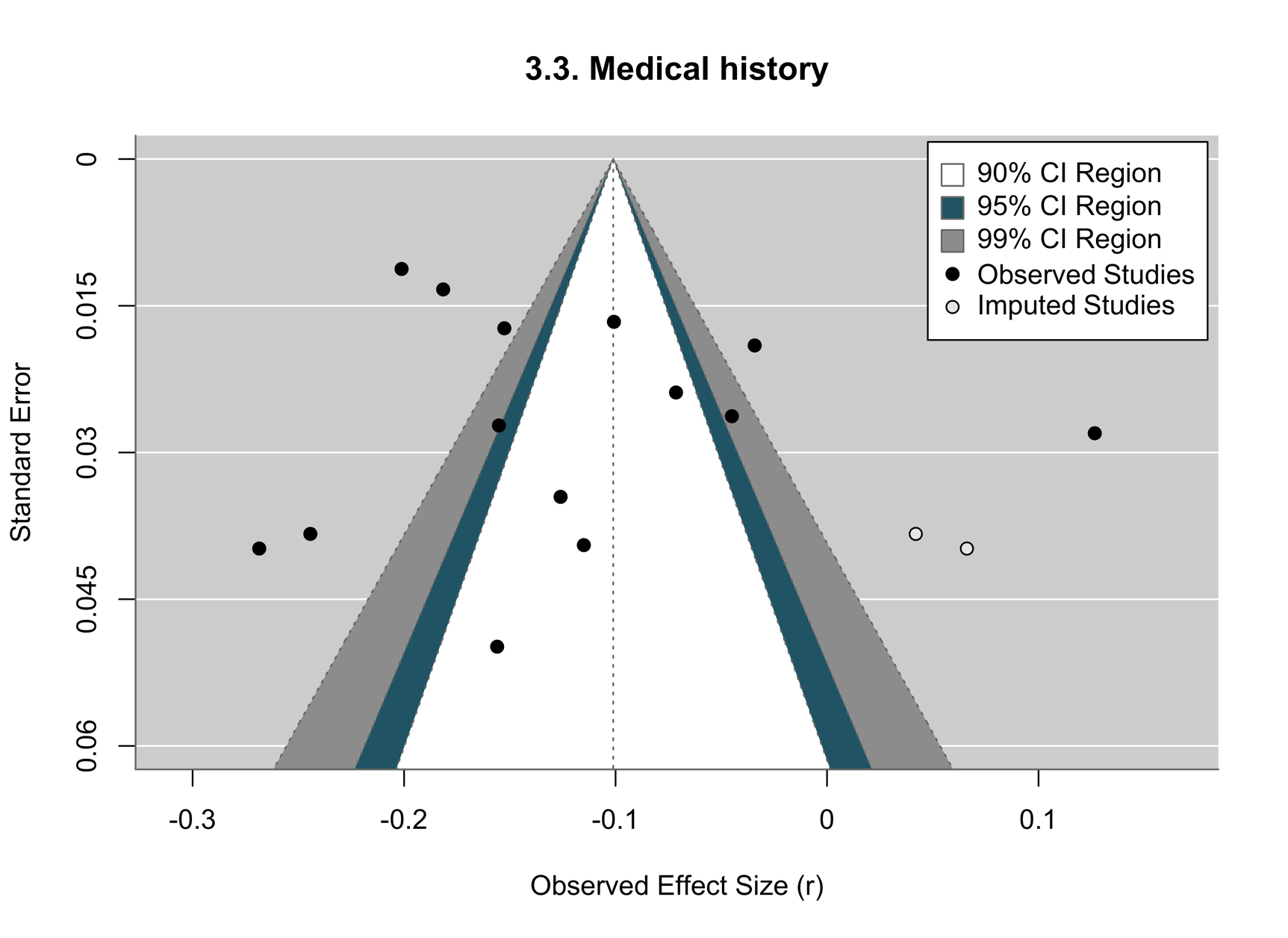


**eFigure 30.** Funnel plot for medical history as a predictor of medical information avoidance, after applying Duval and Tweedie’s trim and fill method, with 90%, 95% and 99% confidence interval (CI) regions.


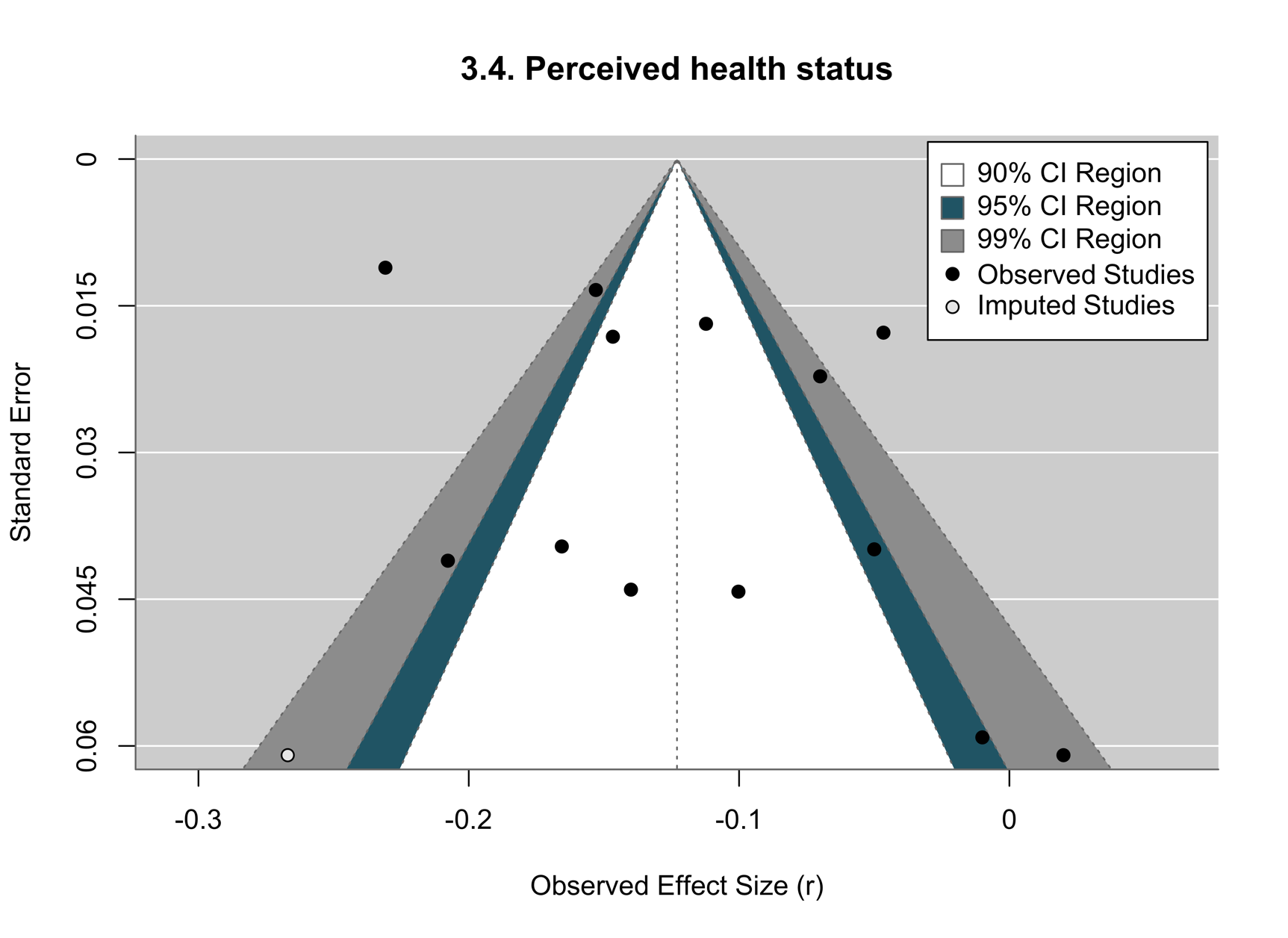


**eFigure 31.** Funnel plot for perceived health status as a predictor of medical information avoidance, after applying Duval and Tweedie’s trim and fill method, with 90%, 95% and 99% confidence interval (CI) regions.


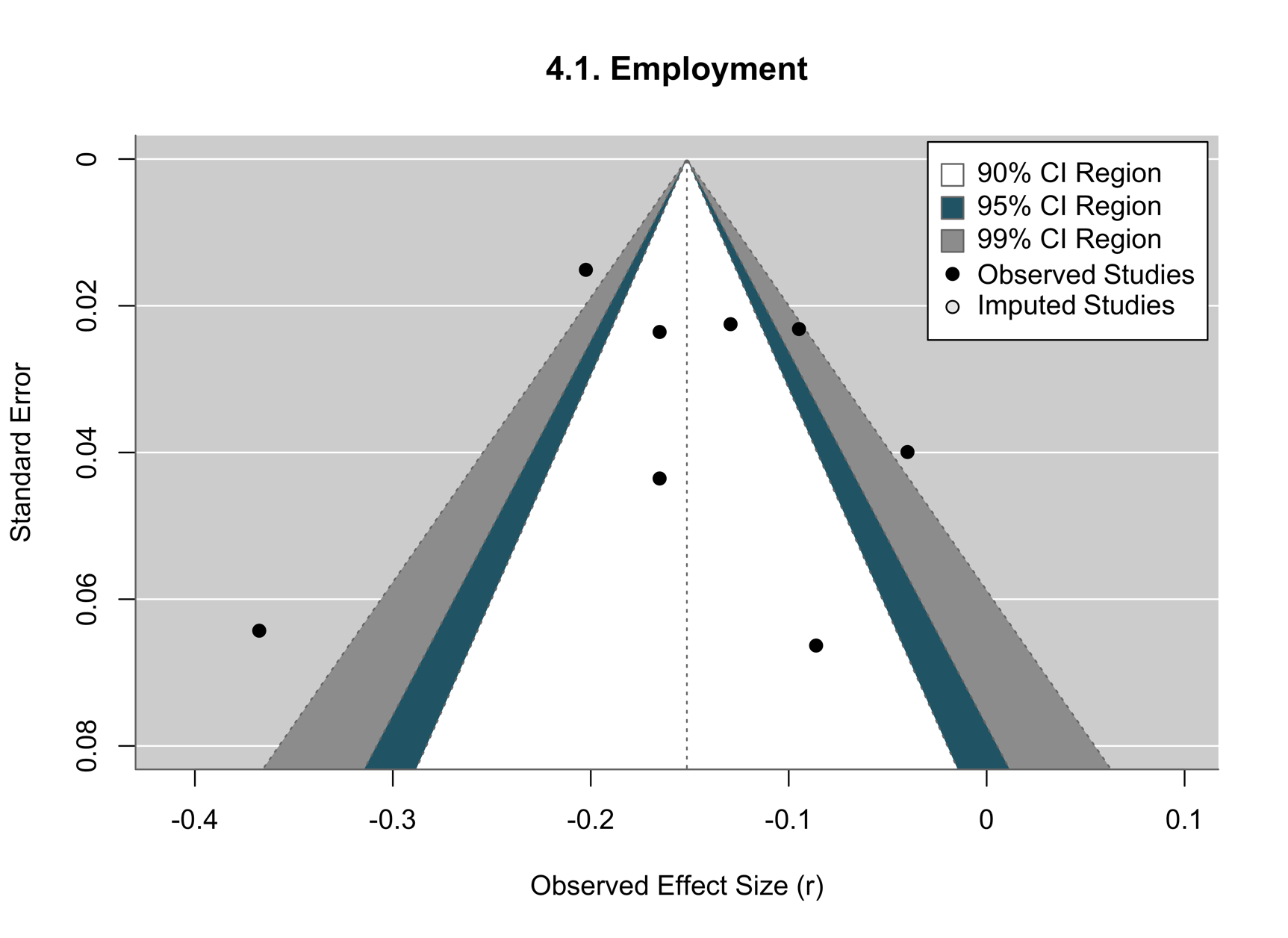


**eFigure 32.** Funnel plot for employment as a predictor of medical information avoidance, after applying Duval and Tweedie’s trim and fill method, with 90%, 95% and 99% confidence interval (CI) regions.


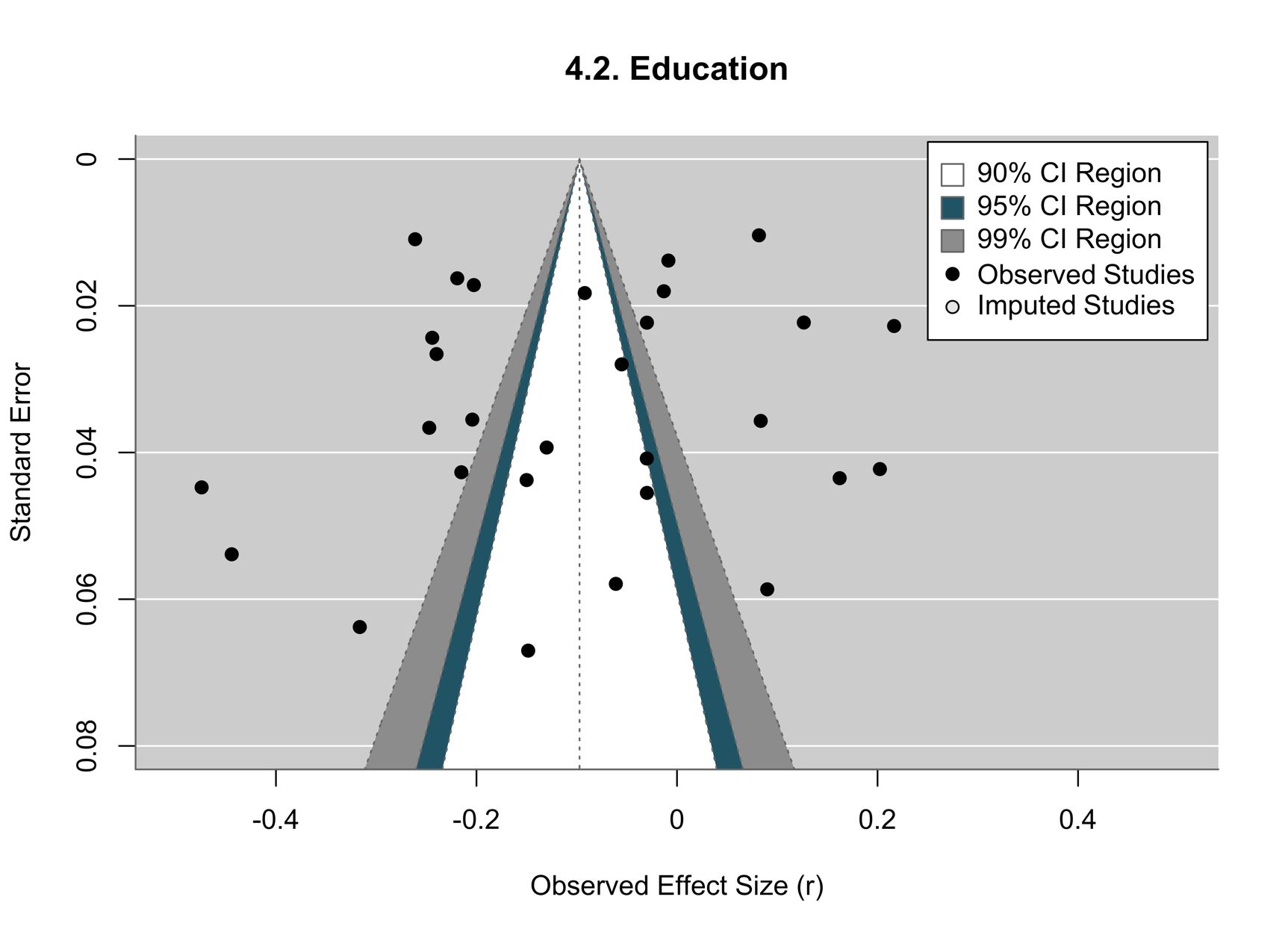


**eFigure 33.** Funnel plot for education as a predictor of medical information avoidance, after applying Duval and Tweedie’s trim and fill method, with 90%, 95% and 99% confidence interval (CI) regions.


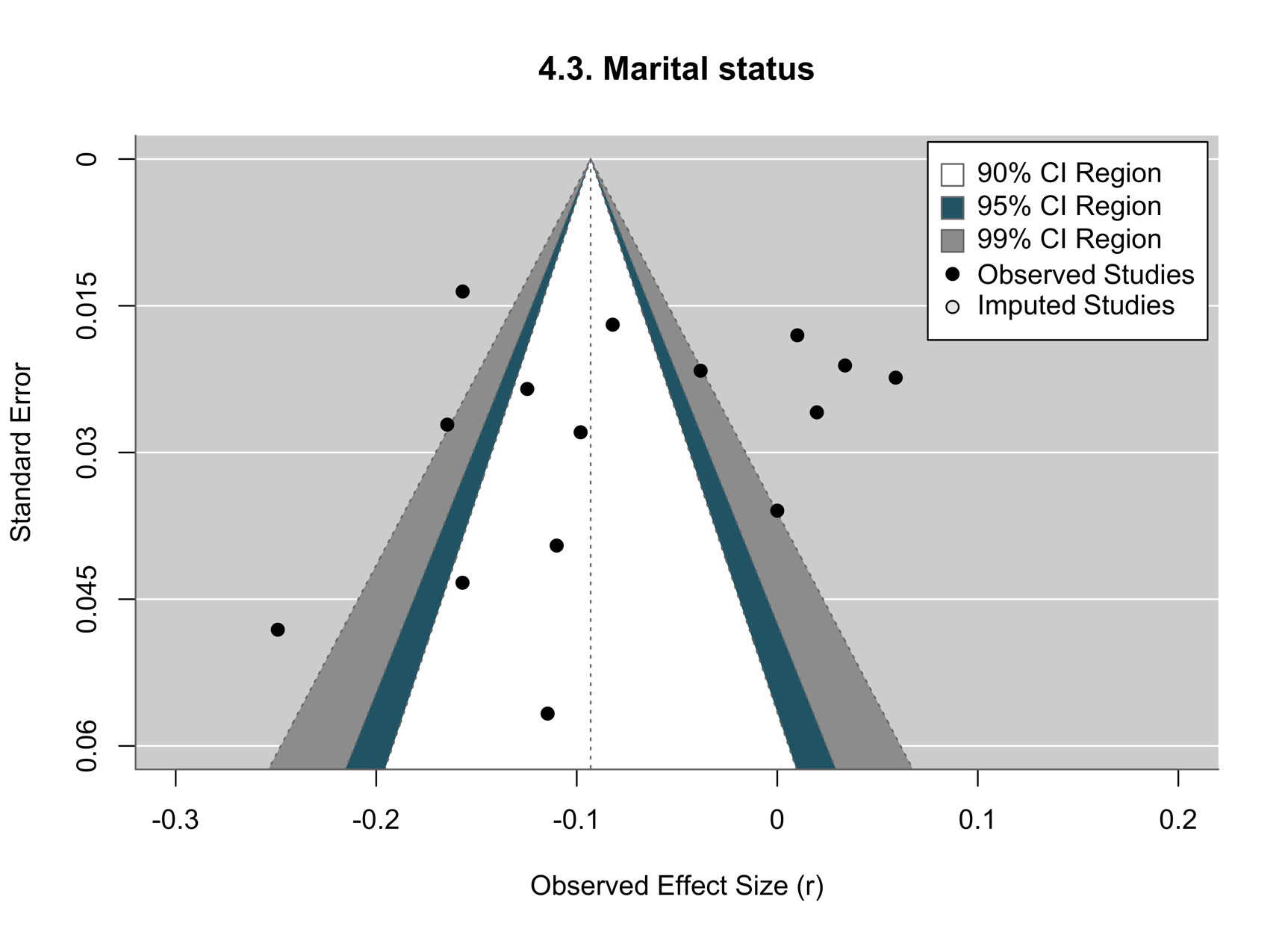


**eFigure 34.** Funnel plot for marital status as a predictor of medical information avoidance, after applying Duval and Tweedie’s trim and fill method, with 90%, 95% and 99% confidence interval (CI) regions.


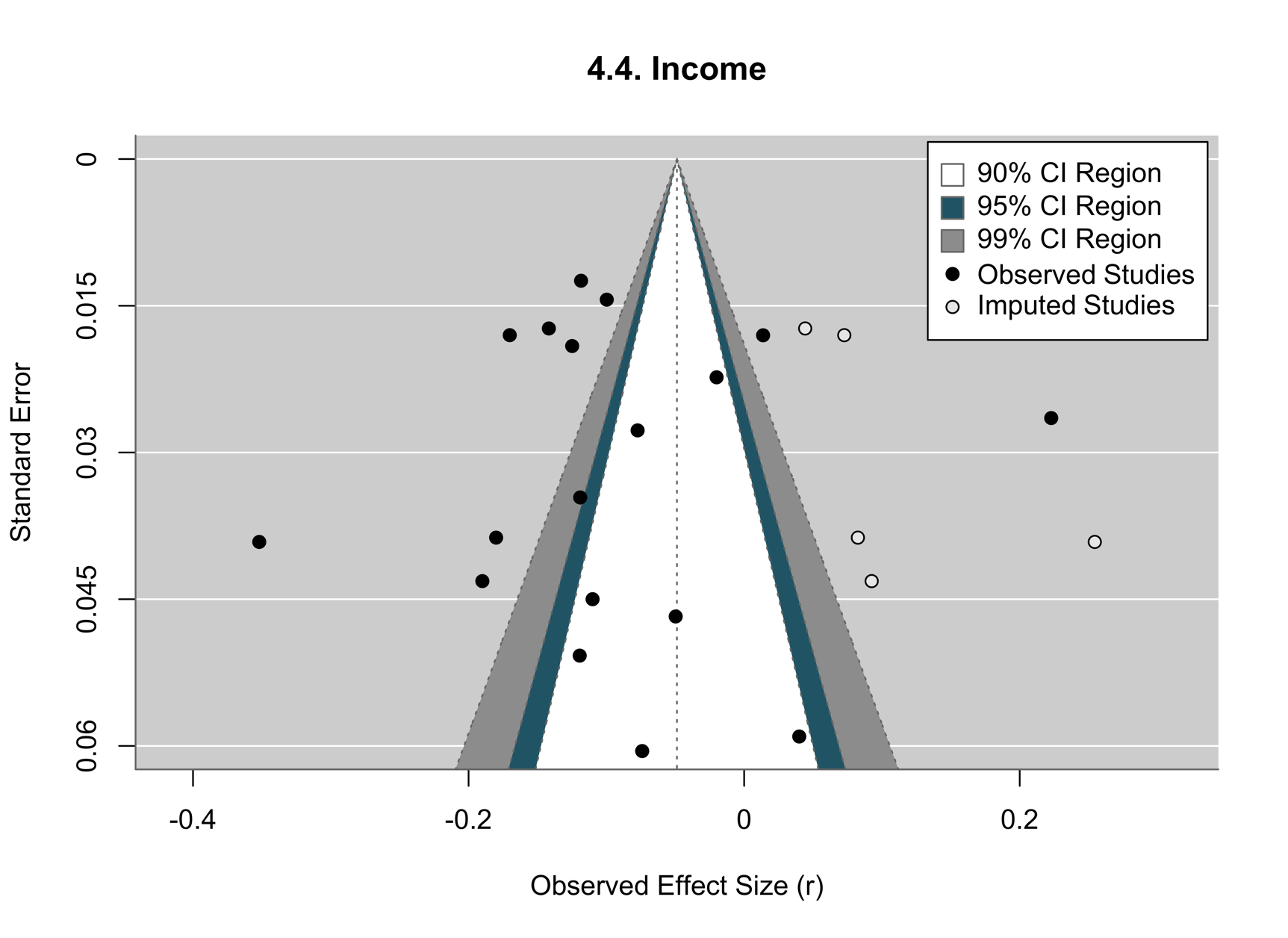


**eFigure 35.** Funnel plot for income as a predictor of medical information avoidance, after applying Duval and Tweedie’s trim and fill method, with 90%, 95% and 99% confidence interval (CI) regions.


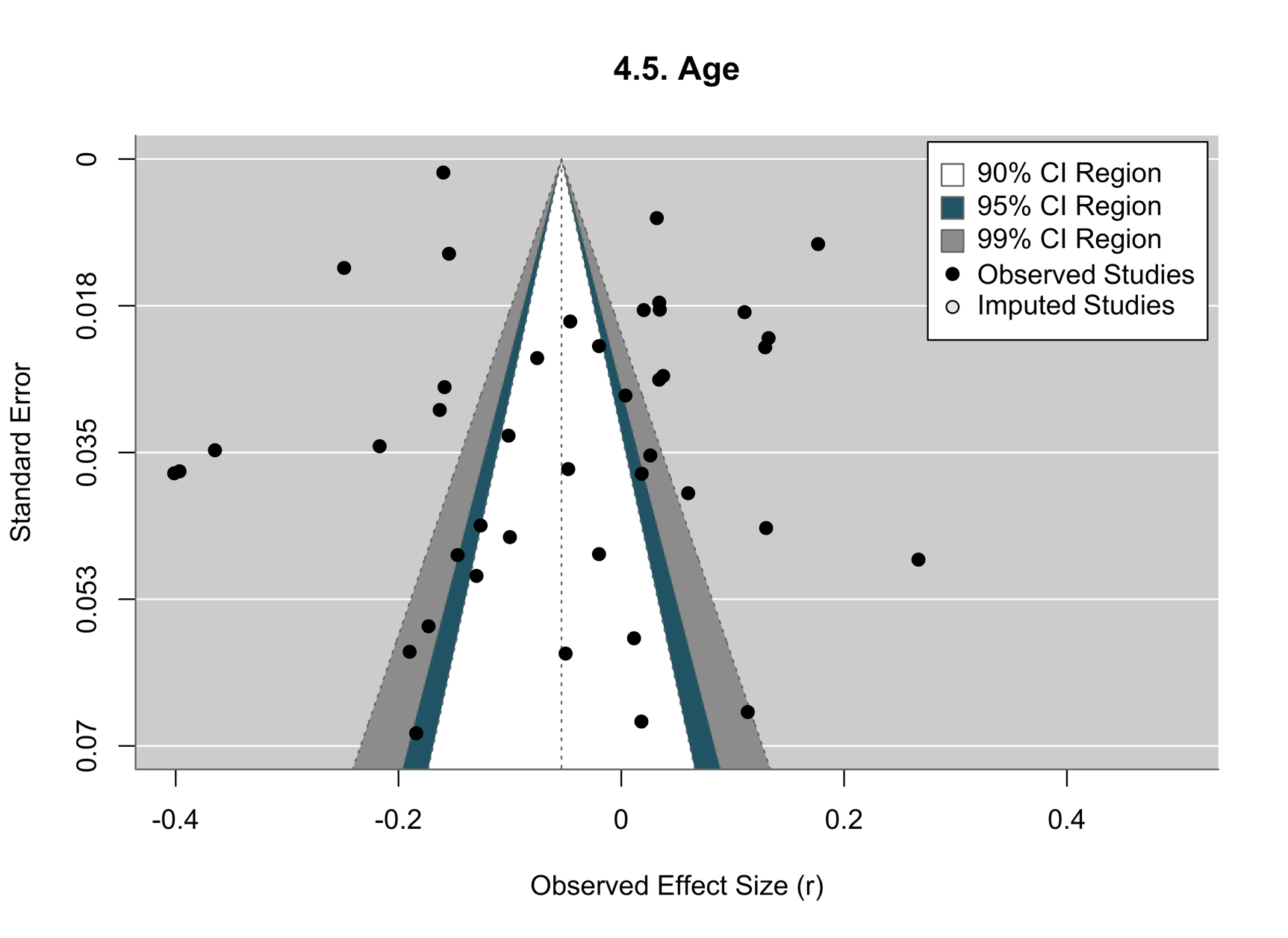


**eFigure 36.** Funnel plot for age as a predictor of medical information avoidance, after applying Duval and Tweedie’s trim and fill method, with 90%, 95% and 99% confidence interval (CI) regions.


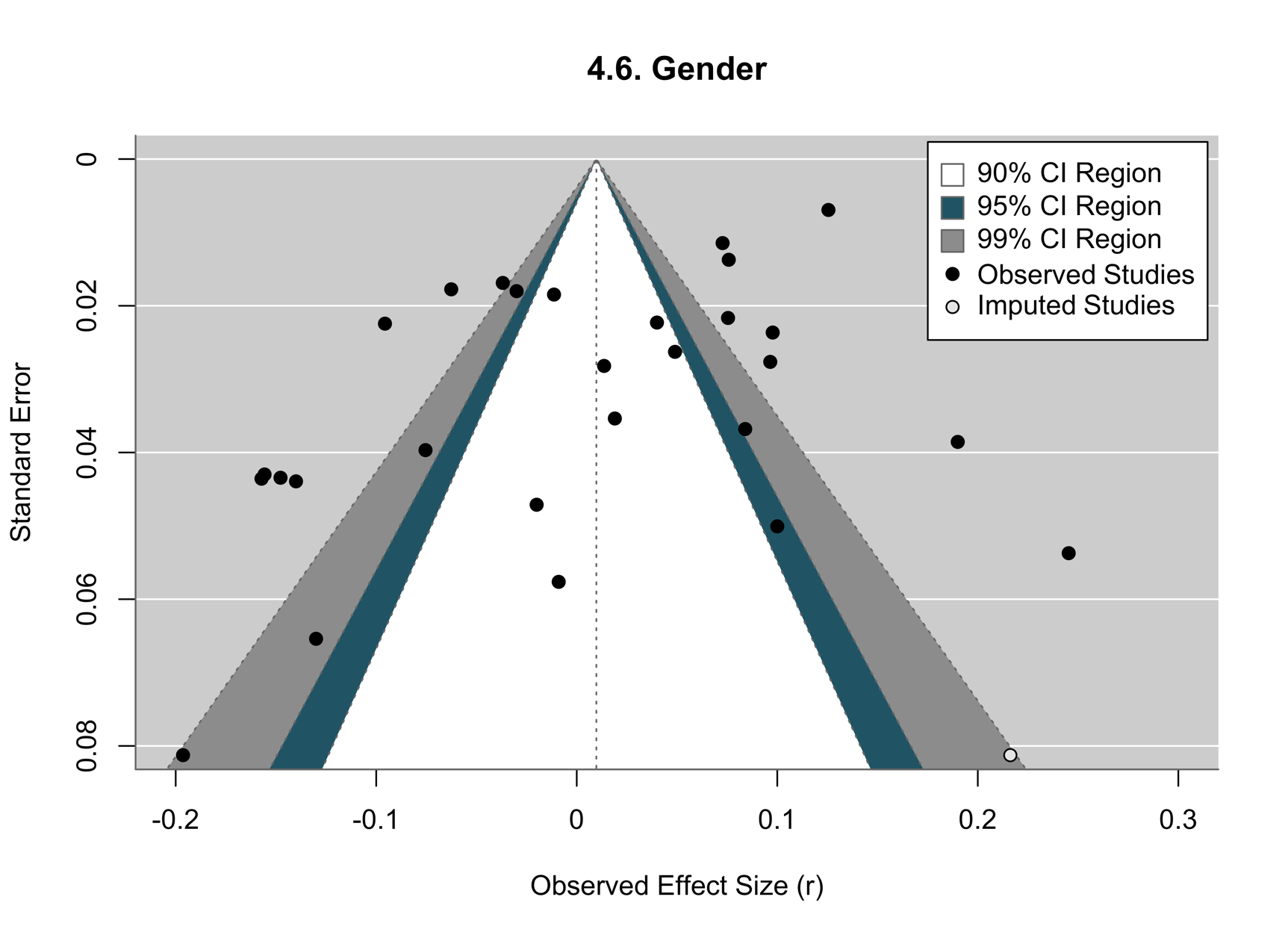


**eFigure 37.** Funnel plot for gender as a predictor of medical information avoidance, after applying Duval and Tweedie’s trim and fill method, with 90%, 95% and 99% confidence interval (CI) regions.


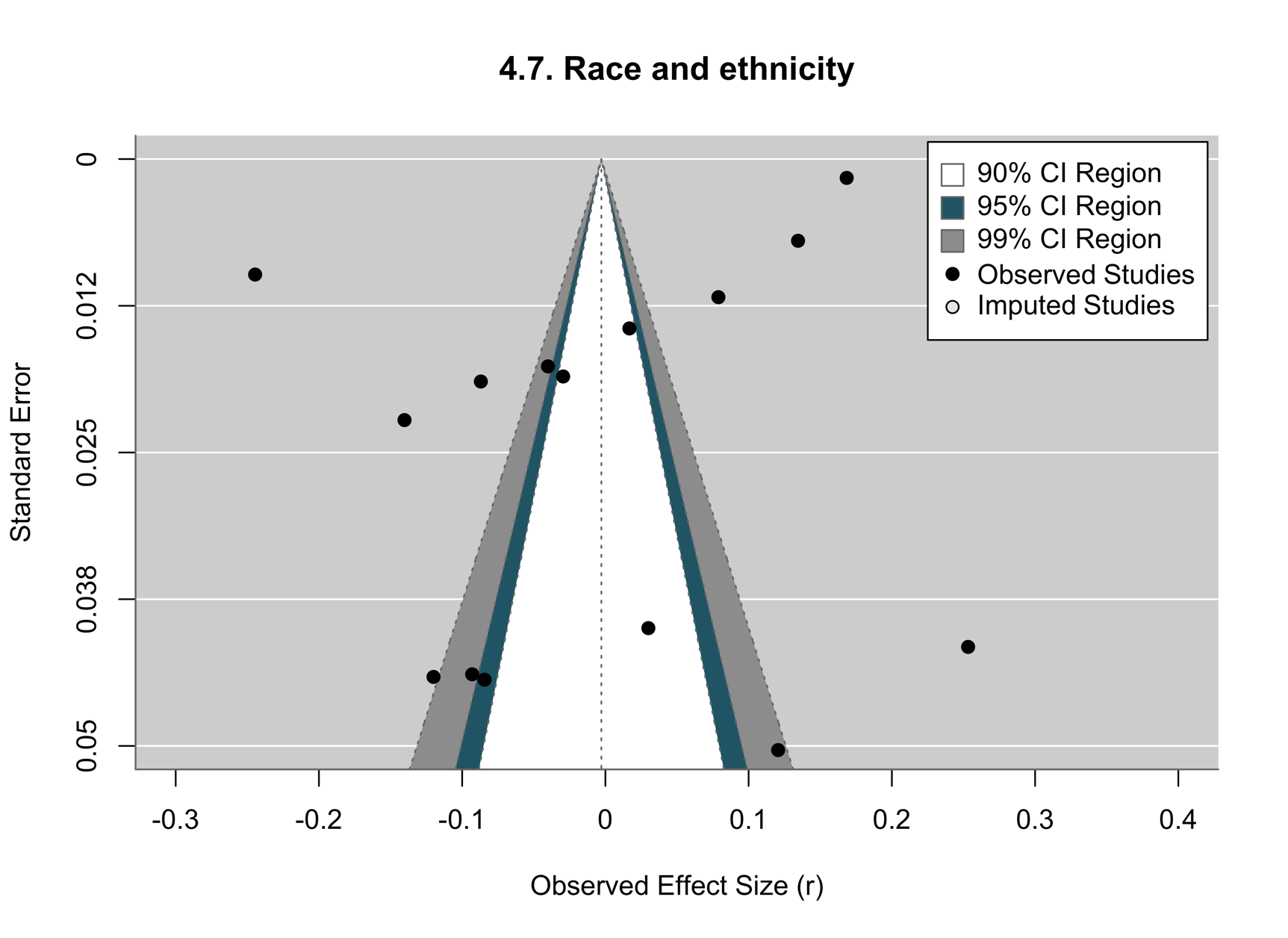


**eFigure 38.** Funnel plot for race and ethnicity as a predictor of medical information avoidance, after applying Duval and Tweedie’s trim and fill method, with 90%, 95% and 99% confidence interval (CI) regions.

**References**

1. Thielmann I, Spadaro G, Balliet D. Personality and prosocial behavior: A theoretical framework and meta-analysis. *Psychol Bull*. 2020;146(1):30-90. doi:10.1037/bul0000217

2. De Winter JCF, Gosling SD, Potter J. Comparing the Pearson and Spearman correlation coefficients across distributions and sample sizes: A tutorial using simulations and empirical data. *Psychol Methods*. 2016;21(3):273-290. doi:10.1037/met0000079

3. Higgins JPT. Measuring inconsistency in meta-analyses. *BMJ*. 2003;327(7414):557-560. doi:10.1136/bmj.327.7414.557

4. Cochran WG. The comparison of percentages in matched samples. *Biometrika*. 1950;37(3/4):256-266. doi:10.2307/2332378

5. Rosenthal R. The file drawer problem and tolerance for null results. *Psychol Bull*. 1979;86(3):638-641. doi:10.1037/0033-2909.86.3.638

6. Egger M, Smith GD, Schneider M, Minder C. Bias in meta-analysis detected by a simple, graphical test. *BMJ*. 1997;315(7109):629-634. doi:10.1136/bmj.315.7109.629

7. Duval S, Tweedie R. Trim and fill: A simple funnel‐plot–based method of testing and adjusting for publication bias in meta‐analysis. *Biometrics*. 2000;56(2):455-463. doi:10.1111/j.0006-341X.2000.00455.x

8. Duval S, Tweedie R. A Nonparametric “trim and fill” method of accounting for publication bias in meta-analysis. *J Am Stat Assoc*. 2000;95(449):89-98. doi:10.1080/01621459.2000.10473905

9. Boyd P, Murray AB, Hyams T, et al. Neuroticism, cancer mortality salience, and physician avoidance in cancer survivors: Proximity of treatment matters. *Psychooncology*. 2022;31(4):641-648. doi:10.1002/pon.5850

10. Chae J. A three-factor cancer-related mental condition model and its relationship with cancer information use, cancer information avoidance, and screening intention. *J Health Commun*. 2015;20(10):1133-1142. doi:10.1080/10810730.2015.1018633

11. Chae J. Who avoids cancer information? Examining a psychological process leading to cancer information avoidance. *J Health Commun*. 2016;21(7):837-844. doi:10.1080/10810730.2016.1177144

12. Chae J, Lee CJ, Kim K. Prevalence, predictors, and psychosocial mechanism of cancer information avoidance: Findings from a national survey of US adults. *Health Commun*. 2020;35(3):322-330. doi:10.1080/10410236.2018.1563028

13. Chen X, Li M, Kreps GL. Double burden of COVID-19 knowledge deficit: Low health literacy and high information avoidance. *BMC Res Notes*. 2022;15(1):1-7. doi:10.1186/s13104-022-05913-8

14. Dahl V, Mellhammar L, Bajunirwe F, Bjorkman P. Acceptance of HIV testing among women attending antenatal care in south-western Uganda: Risk factors and reasons for test refusal. *AIDS Care*. 2008;(6):746-752. doi:10.1080/09540120701693990

15. Dinh T, Detels R, Nguyen M. Factors associated with declining HIV testing and failure to return for results among pregnant women in Vietnam. *AIDS*. 2005;19(11):1234-1236. doi:10.1097/01.aids.0000176228.09474.59

16. Dong Y, Zhang L, Lam C, Huang Z. Counteracting sexual and reproductive health misperceptions: Investigating the roles of stigma, misinformation exposure, and information overload. *Patient Educ Couns*. 2024;120:1-8. doi:10.1016/j.pec.2023.108098

17. Dwyer LA, Shepperd JA, Stock ML. Predicting avoidance of skin damage feedback among college students. *Ann Behav Med*. 2015;49(5):685-695. doi:10.1007/s12160-015-9703-6

18. Emanuel AS, Kiviniemi MT, Howell JL, et al. Avoiding cancer risk information. *Soc Sci Med*. 2015;147:113-120. doi:10.1016/j.socscimed.2015.10.058

19. Fernandez-Balbuena S, Hoyos J, Rosales-Statkus ME, et al. Low HIV testing uptake following diagnosis of a sexually transmitted infection in Spain: Implications for the implementation of efficient strategies to reduce the undiagnosed HIV epidemic. *AIDS Care*. 2016;28(6):677-683. doi:10.1080/09540121.2015.1123808

20. Gustafson CR, Brooks KR, Meerza SIA, Yiannaka A. Emotional responses to COVID-19 stressors increase information avoidance about an important unrelated health threat. *PLoS One*. 2023;18(9). doi:10.1371/journal.pone.0286712

21. Gutierrez E, Candela Iglesias M, Javier Quezada-Juarez F, Rodriguez-Estrada E, Reyes-Teran G, Patricia Caballero-Suarez N. Why individuals fail to collect HIV-test results: an exploratory study at a testing and counseling center in Mexico City. *Rev Panam Salud Publica*. 2018;42:1-7. doi:10.26633/RPSP.2018.14

22. [dataset] National Cancer Institute. 2003. Health Information National Trends Survey (HINTS). Updated May 2023. https://hints.cancer.gov/

23. [dataset] National Cancer Institute. 2009. Health Information National Trends Survey (HINTS), Puerto Rico. https://hints.cancer.gov/

24. [dataset] National Cancer Institute. 2012. Health Information National Trends Survey (HINTS) 4, Cycle 2. Updated October 2020. https://hints.cancer.gov/

25. [dataset] National Cancer Institute. 2014. Health Information National Trends Survey (HINTS) 4, Cycle 4. Updated June 2021. https://hints.cancer.gov/

26. [dataset] National Cancer Institute. 2017. Health Information National Trends Survey (HINTS) 5, Cycle 1. Updated May 2024. https://hints.cancer.gov/

27. [dataset] National Cancer Institute. 2019. Health Information National Trends Survey (HINTS) 5, Cycle 3. Updated May 2024. https://hints.cancer.gov/

28. Hightow L, Miller W, Leone P, Wohl D, Smurzynski M, Kaplan A. Failure to return for HIV posttest counseling in an STD clinic population. *AIDS Educ Prev*. 2003;15(3):282-290. doi:10.1521/aeap.15.4.282.23826

29. Howell JL, Crosier BS, Shepperd JA. Does lacking threat-management resources increase information avoidance? A multi-sample, multi-method investigation. *J Res Pers*. 2014;50:102-109. doi:10.1016/j.jrp.2014.03.003

30. Hvidberg L, Virgilsen LF, Pedersen AF, Vedsted P. Cancer beliefs and participation in screening for colorectal cancer: A Danish cohort study based on data from the International Cancer Benchmarking Partnership and national registers. *Prev Med*. 2019;121:11-17. doi:10.1016/j.ypmed.2019.01.018

31. Ivanova A, Kvalem IL. Psychological predictors of intention and avoidance of attending organized mammography screening in Norway: Applying the Extended Parallel Process Model. *BMC Womens Health*. 2021;21(1):1-14. doi:10.1186/s12905-021-01201-y

32. Jean B, Jindal G, Liao Y. Is ignorance really bliss?: Exploring the interrelationships among information avoidance, health literacy and health justice. *Proc Assoc Info Sci Tech*. 2017;54(1):394-404. doi:10.1002/pra2.2017.14505401043

33. Jung M. Associations of self-rated health and socioeconomic status with information seeking and avoiding behavior among post-treatment cancer patients. *Asian Pac J Cancer Prev*. 2014;15(5):2231-2238. doi:10.7314/APJCP.2014.15.5.2231

34. Kannan VD, Veazie PJ. Predictors of avoiding medical care and reasons for avoidance behavior. *Med Care*. 2014;(4):336-345. doi:10.1097/MLR.0000000000000100

35. Laanani M, Dozol A, Meyer L, et al. Factors associated with failure to return for HIV test results in a free and anonymous screening centre. *Int J STD AIDS*. 2015;26(8):549-555. doi:10.1177/0956462414545795

36. Ladner J, Leroy V, Msellati P, et al. A cohort study of factors associated with failure to return for HIV post-test counselling in pregnant women: Kigali, Rwanda, 1992-1993. *AIDS*. 1996;10(1):69-75. doi:10.1097/00002030-199601000-00010

37. Lee J. Responses to media coverage of the COVID-19 pandemic and information behaviour in the Japanese context. *J Media Commun Res*. 2021;13(1):111-126.

38. Liao Y, Jindal G, St Jean B. The role of self-efficacy in cancer information avoidance. In: Chowdhury G, McLeod J, Gillet V, Willett P, eds. *Transforming Digital Worlds. iConference 2018*. Vol 10766. Lecture Notes in Computer Science. 2018:498-508. doi:10.1007/978-3-319-78105-1_54

39. Liddicoat RV, Losina E, Kang M, Freedberg KA, Walensky RP. Refusing HIV testing in an urgent care setting: Results from the “Think HIV” program. *AIDS Patient Care STDS*. 2006;20(2):84-92. doi:10.1089/apc.2006.20.84

40. Lipsey NP, Shepperd JA. Powerful audiences are linked to health information avoidance: Results from two surveys. *Soc Sci Med*. 2019;225:51-59. doi:10.1016/j.socscimed.2019.01.046

41. Lipsey NP, Shepperd JA. The role of powerful audiences in health information avoidance. *Soc Sci Med*. 2019;220:430-439. doi:10.1016/j.socscimed.2018.11.037

42. Liu M, Chen Y, Shi D, Yan T. The public’s risk information seeking and avoidance in China during early stages of the COVID-19 outbreak. *Front Psychol*. 2021;12:1-12. doi:10.3389/fpsyg.2021.649180

43. Liu J, Li H, Shen W, He Y, Zhu L. How to cope with the negative health information avoidance behavior in a pandemic: the role of resilience. *Behav Inf Technol*. Published online 2024:1-17. doi:10.1080/0144929X.2024.2314746

44. Loiselle CG. Cancer information-seeking preferences linked to distinct patient experiences and differential satisfaction with cancer care. *Patient Educ Couns*. 2019;102(6):1187-1193. doi:10.1016/j.pec.2019.01.009

45. Lu L, Liu J, Yuan YC. Cultural differences in cancer information acquisition: Cancer risk perceptions, fatalistic beliefs, and worry as predictors of cancer information seeking and avoidance in the US and China. *Health Commun*. 2022;37(11):1442-1451. doi:10.1080/10410236.2021.1901422

46. Marlow LAV, Ferrer RA, Chorley AJ, Haddrell JB, Waller J. Variation in health beliefs across different types of cervical screening non-participants. *Prev Med*. 2018;111:204-209. doi:10.1016/j.ypmed.2018.03.014

47. McCloud RF, Jung M, Gray SW, Viswanath K. Class, race and ethnicity and information avoidance among cancer survivors. *Br J Cancer*. 2013;108(10):1949-1956. doi:10.1038/bjc.2013.182

48. McQueen A, Swank PR, Vernon SW. Examining patterns of association with defensive information processing about colorectal cancer screening. *J Health Psychol*. 2014;(11):1443-1458. doi:10.1177/1359105313493649

49. Melnyk D. *When We Do Not Want to Know: The Information Avoidance Model* [dissertation]. University of Florida; 2010.

50. Miles A, Voorwinden S, Chapman S, Wardle J. Psychologic predictors of cancer information avoidance among older adults: The role of cancer fear and fatalism. *Cancer Epidemiol Biomarkers Prev*. 2008;17(8):1872-1879. doi:10.1158/1055-9965.EPI-08-0074

51. Mmbaga EJ, Leyna GH, Mnyika KS, Hussain A, Klepp KI. Prevalence and predictors of failure to return for HIV-1 post-test counseling in the era of antiretroviral therapy in rural Kilimanjaro, Tanzania: Challenges and opportunities. *AIDS Care*. 2009;21(2):160-167. doi:10.1080/09540120801982905

52. Molitor F, Bell R, Truax S, Ruiz J, Sun R. Predictors of failure to return for HIV test result and counseling by test site type. *AIDS Educ Prev*. 1999;11(1):1-13.

53. Moreira Vasconcelos CT, Fernandes Cunha D de F, Coelho CF, Bezerra Pinheire AK, Sawada NO. Factors related to failure to attend the consultation to receive the results of the Pap smear test. *Rev Lat Am Enfermagem*. 2014;22(3):401-407. doi:10.1590/0104-1169.3132.2430

54. Moser RP, Arndt J, Han PK, Waters EA, Amsellem M, Hesse BW. Perceptions of cancer as a death sentence: Prevalence and consequences. *J Health Psychol*. 2014;19(12):1518-1524. doi:10.1177/1359105313494924

55. Msuya SE, Mbizvo E, Uriyo J, Stray-Pedersen B, Sam NE, Hussain A. Predictors of failure to return for HIV test results among pregnant women in Moshi, Tanzania. *J Acquir Immune Defic Syndr*. 2006;43(1):85-90. doi:10.1097/01.qai.0000225016.50890.7e

56. Nelissen S, Beullens K, Lemal M, Van den Bulck J. Fear of cancer is associated with cancer information seeking, scanning and avoiding: a cross-sectional study among cancer diagnosed and non-diagnosed individuals. *Health Info Libr J*. 2015;32(2):107-119. doi:10.1111/hir.12100

57. Nolte J, Deng SL, Löckenhoff CE. Age differences in media consumption and avoidance with respect to COVID-19. Gutchess A, ed. *J Gerontol B*. 2022;77(4):e76-e82. doi:10.1093/geronb/gbab123

58. Orom H, Stanar S, Allard NC, et al. Reasons people avoid colorectal cancer information: A mixed-methods study. *Psychol Health*. Published online 2023. doi:10.1080/08870446.2023.2280177

59. Peng W, Carcioppolo N, Occa A, Ali K, Yang Q, Yang F. Feel worried, overloaded, or fatalistic? The determinants of cancer uncertainty management preferences. *Health Commun*. 2021;36(3):347-360. doi:10.1080/10410236.2019.1692489

60. Persoskie A, Ferrer RA, Klein WMP. Association of cancer worry and perceived risk with doctor avoidance: an analysis of information avoidance in a nationally representative US sample. *J Behav Med*. 2014;37(5):977-987. doi:10.1007/s10865-013-9537-2

61. Pisculli ML, Reichmann WM, Losina E, et al. Factors associated with refusal of rapid HIV testing in an emergency department. *AIDS Behav*. 2011;(4):734-742. doi:10.1007/s10461-010-9837-2

62. Price DM, Howell JL, Gesselman AN, Finneran S, Quinn DM, Eaton LA. Psychological threat avoidance as a barrier to HIV testing in gay/bisexual men. *J Behav Med*. 2019;42(3):534-544. doi:10.1007/s10865-018-0003-z

63. Rao A, Kennedy C, Mda P, Quinn TC, Stead D, Hansoti B. Patient acceptance of HIV testing services in rural emergency departments in South Africa. *South Afr J HIV Med*. 2020;21(1). doi:10.4102/sajhivmed.v21i1.1105

64. Rolland C, de La Rochebrochard E, Piron P, Shelly M, Segouin C, Troude P. Who fails to return within 30 days after being tested positive for HIV/STI in a free testing centre? *BMC Infect Dis*. 2020;20(1):1-9. doi:10.1186/s12879-020-05520-7

65. Sesay C, Chien LY. Analysis of factors associated with failure to return for an HIV-test result in The Gambia. *Afr J AIDS Res*. 2012;11(2):83-89. doi:10.2989/16085906.2012.698053

66. Shepperd JA, Emanuel AS, Howell JL, Logan HL. Predicting scheduling and attending for an oral cancer examination. *Ann Behav Med*. 2015;49(6):828-838. doi:10.1007/s12160-015-9717-0

67. Siebenhaar KU, Koether AK, Alpers GW. Dealing with the COVID-19 infodemic: Distress by information, information avoidance, and compliance with preventive measures. *Front Psychol*. 2020;11. doi:10.3389/fpsyg.2020.567905

68. Simon PA, Weber M, Ford WL, Cheng F, Kerndt PR. Reasons for HIV antibody test refusal in a heterosexual sexually transmitted disease clinic population. *AIDS*. 1996;(13):1549-1553. doi:10.1097/00002030-199611000-00014

69. Simon KA, Driver R, Rathus T, et al. HIV information avoidance, HIV stigma, and medical mistrust among Black sexual minority men in the southern United States: Associations with HIV testing. *AIDS Behav*. 2024;28(1):12-18. doi:10.1007/s10461-023-04218-6

70. Suan MAM. Return for postpartum oral glucose tolerance test following gestational diabetes mellitus. *Asia Pac J Public Health*. 2015;27(6):601-609. doi:10.1177/1010539515588943

71. Tucker JA, Chandler SD, Cheong J. Predicting HIV testing in low threshold community contexts among young African American women living in the Southern United States. *AIDS Care*. 2020;(2):175-181. doi:10.1080/09540121.2019.1668522

72. Vrinten C, Boniface D, Lo SH, Kobayashi LC, von Wagner C, Waller J. Does psychosocial stress exacerbate avoidant responses to cancer information in those who are afraid of cancer? A population-based survey among older adults in England. *Psychol Health*. 2018;33(1):117-129. doi:10.1080/08870446.2017.1314475

73. Zhang K, Zhang N, Wang J, Jiang J, Xu S. Exploring the roles of fear and powerlessness in the relationship between perceived risk of the COVID-19 pandemic and information-avoidance behavior. *Front Psychol*. 2022;13. doi:10.3389/fpsyg.2022.1005142

74. Zhao S, Liu Y. The more insufficient, the more avoidance? Cognitive and affective factors that relates to information behaviours in acute risks. *Front Psychol*. 2021;12:1-11. doi:10.3389/fpsyg.2021.730068

**References:**^9^,^10^,^11^,^12^,^13^,^14^,^15^,^16^,^17^,^18^,^19^,^20^,^21^,^22^,^23^,^24^,^25^,^26^,^27^,^28^,^29^,^30^,^31^,^32^,^33^,^34^,^35^,^36^,^37^,^38^,^39^,^40^,^41^,^42^,^43^,^44^,^45^,^46^,^47^,^48^,^49^,^50^,^51^,^52^,^53^,^54^,^55^,^56^,^57^,^58^,^59^,^60^,^61^,^62^,^63^,^64^,^65^,^66^,^67^,^68^,^69^,^70^,^71^,^72^,^73^,^74^

1. Correspondence: Max Planck Institute for Human Development, Center for Adaptive Rationality (ARC), Lentzeallee 94, 14195 Berlin, Germany, Phone: +49-30-82406-496, Email: offer@mpib-berlin.mpg.de [↑](#footnote-ref-2)
2. The R script by Thielmann et al.,^1^ 2020, is publicly available on the Open Science Framework (<https://osf.io/dbuk6/>). We thank Isabel Thielmann for her helpful suggestions and comments on the transformations and aggregations of different effect sizes for meta-analytic estimates. [↑](#footnote-ref-3)
